# *In situ* CO_2_ capture: a general strategy for valorizing waste polycarbonate into high-value chemicals

**DOI:** 10.1093/nsr/nwag153

**Published:** 2026-03-10

**Authors:** Minghao Zhang, Shaoyu Zhang, Siming Zhu, Siyu Zhang, Zexiang Wu, Weixiang Wu, Qingqing Mei

**Affiliations:** State Key Laboratory of Soil Pollution Control and Safety, Zhejiang University, Hangzhou 310058, China; College of Environmental and Resource Sciences, Zhejiang University, Hangzhou 310058, China; State Key Laboratory of Soil Pollution Control and Safety, Zhejiang University, Hangzhou 310058, China; College of Environmental and Resource Sciences, Zhejiang University, Hangzhou 310058, China; State Key Laboratory of Soil Pollution Control and Safety, Zhejiang University, Hangzhou 310058, China; College of Environmental and Resource Sciences, Zhejiang University, Hangzhou 310058, China; State Key Laboratory of Soil Pollution Control and Safety, Zhejiang University, Hangzhou 310058, China; College of Environmental and Resource Sciences, Zhejiang University, Hangzhou 310058, China; State Key Laboratory of Soil Pollution Control and Safety, Zhejiang University, Hangzhou 310058, China; College of Environmental and Resource Sciences, Zhejiang University, Hangzhou 310058, China; State Key Laboratory of Soil Pollution Control and Safety, Zhejiang University, Hangzhou 310058, China; College of Environmental and Resource Sciences, Zhejiang University, Hangzhou 310058, China; State Key Laboratory of Soil Pollution Control and Safety, Zhejiang University, Hangzhou 310058, China; College of Environmental and Resource Sciences, Zhejiang University, Hangzhou 310058, China

**Keywords:** waste plastics, polycarbonate, valuable chemicals, CO_2_ capture, bisphenol A

## Abstract

Efficiently utilizing carbon resources from waste plastics and expanding pathways for high-value chemical production are crucial for sustainable development and advancing the circular economy. However, the challenge of inert and hard-to-reuse CO_2_ released during plastic management remains a significant hurdle, resulting in the loss of valuable carbon resources. Herein, we propose a versatile *in situ* CO_2_-capturing strategy for the efficient upcycling of polycarbonate (PC) to produce bisphenol A (BPA) and diverse CO_2_-upgraded products. Utilizing a variety of CO_2_-capturing reagents, including nitriles, epoxies, amines, alkynes and alcohols, we achieve complete depolymerization of PC under mild, atmospheric conditions and efficiently co-produce value-added chemicals such as nitrogen-containing heterocycles, cyclic carbonates and acetylenic carboxylic acids. Mechanistic studies reveal that the process involves a more thermodynamically favorable *in situ* CO_2_ capture pathway compared to conventional gaseous CO_2_. This strategy offers a new route to unlock carbon resources from carbonate-based polymers for diversified chemical production and sustainable waste management.

## Teaser

This strategy valorizes polycarbonate waste into various high-value chemicals by in-situ capturing released CO2, creating a sustainable upcycling route that reduces environmental footprints and global plastic pollution.

## INTRODUCTION

**Figure 1.**
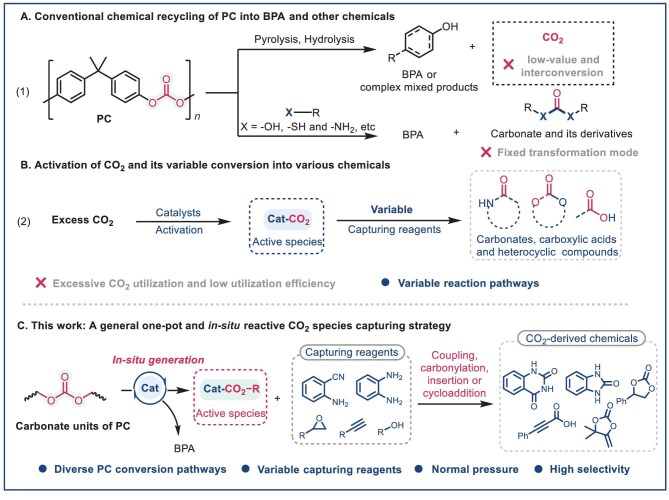
*In situ* capture of CO_2_ released during PC depolymerization. (A) Conventional chemical recycling of PC. (B) Conversion of CO_2_ into diverse chemicals. (C) *In situ* CO_2_ capture strategy in PC upcycling.

The overwhelming production and improper disposal of plastics have led to a significant environmental crisis and resource wastage [1–5]. Poly(bisphenol A carbonate) (PC) is one of the most extensively used plastics with a global annual production reaching approximately 6 million tons [6–8]. Its disposal exacerbates environmental issues through greenhouse gas emissions and toxic substance leakage [6]. Primary mechanical disposal results in lower-grade polymers with reduced properties [9–12]. In contrast, chemical recycling presents a promising alternative by transforming PC into valuable monomers and chemicals such as phenols, alcohols and alkanes [13–19]. Importantly, these recovered chemicals can be further upgraded into advanced functional materials, thereby extending the material life cycle and enhancing overall resource efficiency [20–22]. However, unlike other polyester recycling processes like pyrolysis or hydrolysis, PC degradation often results in CO_2_ release due to the cleavage of its carbonate units (–O–CO_2_–), leading to carbon resource loss and direct emissions (Fig. 1A, Table S1) [23–26]. To mitigate CO_2_ emissions, methods like alcoholysis and ammonolysis target carbonate units by depolymerizing ester bonds with various nucleophilic reagents [7,8,27–29]. These methods, however, are limited by narrow reaction pathways and a restricted variety of derivative chemicals. A paradigm shift towards *in situ* capture of carbonate units within PC structures is desired for more versatile and sustainable approaches.

CO_2_ is increasingly recognized as an abundant and renewable C1 building block with diverse conversion patterns [30,31], enabling the production of a wide range of high-value chemicals (Fig. 1B) [32–35]. Among these CO_2_-derived products, nitrogen-containing heterocyclic compounds, particularly quinazoline-2,4(1*H*,3*H*)-dione (QDO) derivatives, have attracted considerable interest owing to their distinctive molecular scaffolds and broad biological activities. However, the practical synthesis of these compounds remains challenging, largely due to the thermodynamic and kinetic inertness of CO_2_, which typically necessitates elevated pressures and temperatures for effective activation [30,36–38]. Consequently, many existing protocols rely on large excesses of CO_2_, resulting in poor carbon utilization efficiency (Fig. 1B, Figs S1 and S2, Table S2). Recent advances have explored the formation of reactive catalyst–CO_2_ complexes to enhance CO_2_ activation [32,39–41]. Interestingly, catalyst-promoted nucleophilic activation of ester or carbonate bonds also generates highly active R–CO_2_-derived species *in situ* [42–45]. We hypothesize that during catalytic hydrolysis of PC, the catalyst activates the carbonate bond, facilitating simultaneous C–O bond cleavage and formation of reactive R–CO_2_–catalyst complexes (Fig. 1C). These active species may be favorable to being captured *in situ* by trapping agents, enabling synthesis of high-value chemicals while effectively mitigating CO_2_ emissions and addressing the low reactivity of conventional CO_2_ conversion methods with gaseous CO_2_.

Herein, we propose an *in situ* CO_2_ capture strategy for the targeted conversion of PC into bisphenol A (BPA) and a series of CO_2_-derived chemicals. By employing various functionalized CO_2_-capturing reagents (amines, alkynes, alcohols, epoxides and nitrile compounds), we efficiently valorized PC under ambient pressure, addressing intrinsic CO_2_ utilization limitations in conventional PC recycling. The strategy offers several key advantages: (i) it enables diversified transformation modes of the carbonate units in PC, facilitating high-value reactions such as coupling, insertion, carbonylation and cycloaddition, beyond the simple nucleophilic cleavage typical of traditional alcoholysis and ammonolysis; (ii) it exhibits broad compatibility with structurally diverse CO_2_ capture reagents, allowing efficient valorization of CO_2_ as a versatile C1 building block; and (iii) it favorably shifts reaction thermodynamics toward product formation, promoting complete PC depolymerization while reducing overall reagent consumption. This strategy demonstrated that PC could replace CO_2_ in chemical synthesis as an upper-level reactant, exemplified by the complete depolymerization of PC into BPA and QDO, with yields of 99% and 96%, respectively. The method successfully upcycled various commercial PC wastes while producing a series of nitrogen-containing heterocycles, cyclic carbonates and acetylenic carboxylic acids. Overall, this strategy establishes a novel pathway for PC disposal that simultaneously addresses CO_2_ release challenges during PC recycling and valorizing CO_2_ through diversified transformation of PC waste into BPA and a broad range of value-added chemicals.

## RESULTS AND DISCUSSION

### Exploration of depolymerization conditions and scope of substrates

Initially, a series of control experiments were conducted to investigate the reaction parameters using 2-aminobenzonitrile (**2a**, ABN) as the capture reagent (Fig. S3). The results confirmed that both the catalyst and water are essential for the reaction. The optimal reaction conditions were as follows: PC (0.254 g, 1 mmol structural unit), ABN (**2a**, 1 mmol), 1,8-diazabicyclo[5.4.0]undec-7-ene (DBU) (0.4 mmol) and *N*-methyl-2-pyrrolidone (NMP) (2 mL) at 130°C for 4 h, affording BPA and QDO (**4a**) in 99% and 96% yields, respectively (Figs S4 and S5). The obtained product, QDO, could be isolated by the addition of ethyl acetate (Fig. S6). The reaction process was systematically monitored over time using both ^1^H nuclear magnetic resonance (NMR) and Fourier-transform infrared (FT-IR) spectroscopy (Fig. S7).

**Figure 2.**
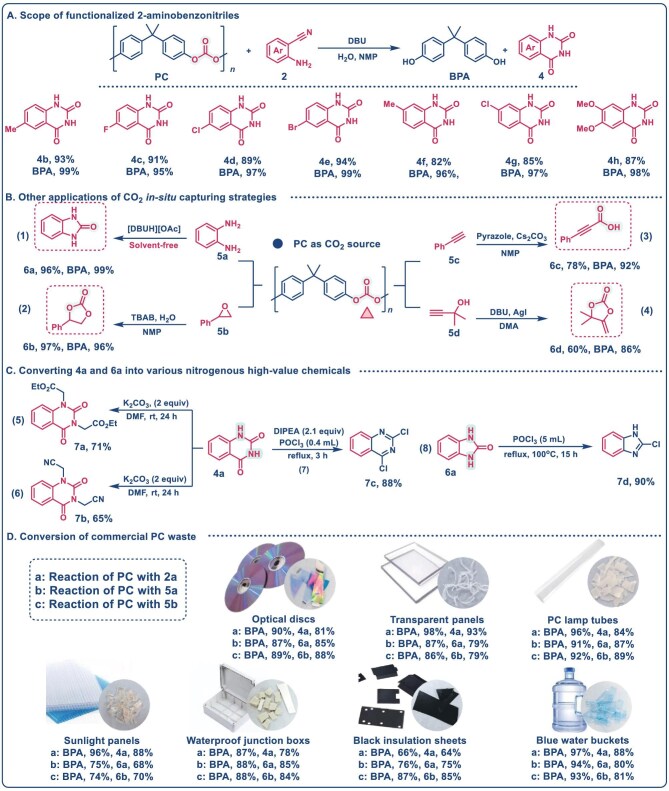
Scope of substrates. (A) Standard reaction conditions: PC (0.254 g, 1 mmol, 1 mmol structural unit), DBU (0.4 mmol), H_2_O (1 mmol), aminobenzonitrile (2 mmol) and NMP (2 mL) at 130°C for 4 h. (B) Standard reaction conditions: (1) PC (0.254 g), [DBUH][OAc] (0.4 mmol) and **5a** (2 mmol) at 130°C for 4 h; (2) PC (0.254 g), tetrabutylammonium bromide (TBAB) (0.1 mmol), H_2_O (2 mmol) and styrene oxide (2 mmol) at 120°C for 6 h; (3) PC (0.254 g), pyrazole (0.1 mmol), Cs_2_CO_3_ (3 mmol) and phenylacetylene (3 mmol) and NMP (2 mL) at 130°C for 6 h; (4) PC (0.254 g), DBU (0.4 mmol), AgI (0.1 mmol) and 2-methylbut-3-yn-2-ol (3 mmol) at 90°C for 10 h. Yield detected by ^1^H NMR. (C) Conversion of **4a** and **6a** into other valuable chemicals. (D) Scope of PC waste.

To synthesize diverse QDO derivatives, a range of functionalized 2-aminobenzonitriles with varying substitutions on the benzene ring were employed to upcycle waste PC, as shown in Fig. 2A. ABN derivatives substituted with electron-donating groups (e.g. 4-methyl, 5-methyl and 4,5-dimethoxy) and electron-withdrawing groups (e.g. 4-Cl, 5-Cl, 5-Br and 5-F) react efficiently with PC, producing the corresponding QDO derivatives with yields ranging from 85% to 94%. Concurrently, BPA was obtained with yields ranging from 95% to 99%. These results demonstrate that the *in situ* CO_2_ capture strategy exhibits excellent substrate adaptability, showing minimal influence from electronic or steric effects of the functional groups. We further optimized the conversion of PC with 1,2-diaminobenzene (**5a**) to yield BPA and 2-benzimidazolone (BMO, **6a**), as depicted in Table S3. By optimizing the reaction parameters, we identified the optimal conditions as follows: PC (0.254 g, 1 mmol structural unit), 1,2-diaminobenzene (**5a**) (2 mmol), and [DBUH][OAc] (30 mol%) at 130°C for 4 h (Fig. 2B, Fig. S8, Table S3). Under optimal solvent-free conditions, complete degradation of PC was achieved, affording BPA and BMO in yields of 99% and 97%, respectively.

The versatility of this *in situ* CO_2_ capture strategy was extended to other transformation reactions, including epoxy-CO_2_ coupling, alkyne-CO_2_ carboxylation and alcohol-CO_2_ cycloaddition (Fig. 2B) [46–48].

These strategies efficiently convert PC into BPA with yields of 86%–99%, along with the corresponding CO_2_-derived chemicals, including 4-phenyl-1,3-dioxolan-2-one, 3-phenylpropiolic acid and 4,4-dimethyl-5-methylene-1,3-dioxolan-2-one, achieving yields of 60%–97%. These results highlight the potential of replacing CO_2_ with PC plastic waste as a feedstock for synthesizing high-value CO_2_-derived chemicals. We also explored the functionalization of the obtained QDO through N–H bond reactions (Fig. 2C). Using N-alkylation reagents such as ethyl chloroacetate and chloroacetonitrile, QDO was successfully converted into diethyl 2,2′-(2,4-dioxoquinazoline-1,3(2*H*,4*H*)-diyl)diacetate and 2,2′-(2,4-dioxoquinazoline-1,3(2*H*,4*H*)-diyl)diacetonitrile, with yields of 71% and 65%, respectively. Additionally, chlorination of QDO and BMO with POCl_3_ produced 2,4-dichloroquinazoline and 2-chloro-1*H*-benzo[*d*]imidazole, with yields of 88% and 90%, respectively. These transformations of the obtained heterocyclic products (QDO and BMO) further validated the application potential of this strategy. Collectively, these results demonstrate the efficiency and versatility of the proposed *in situ* CO_2_ capture strategy for upcycling waste PC into high-value chemicals. Moreover, preliminary techno-economic analysis (TEA) and cradle-to-gate life cycle assessment (LCA) indicate that the proposed waste-PC *in situ* CO_2_ capture route is economically promising and environmentally advantageous for QDO production compared with the conventional CO_2_-based synthesis pathway (global warming potential, GWP: 2.21 vs 4.39 kg CO_2_-eq per kg QDO; Figs S22–S26, Tables S5–S8).

### Kinetic studies and control experiments

Recycling of various types of commercial PC waste was further conducted (Fig. 2D). PC waste samples, including hollow sheets, optical disks, transparent plates, lamp tubes, electric enclosures, black insulation sheets and blue water buckets, were subjected to the *in situ* CO_2_ capture strategy. These plastics were effectively dissolved in NMP, as confirmed through solubility analysis and scanning electron microscopy (SEM, Figs S9 and S10). Under standard conditions, all types of PC plastics were efficiently converted into BPA with yields ranging from 66% to 98%. Simultaneously, CO_2_ was effectively captured *in situ* by reagents such as ABN, 1,2-diaminobenzene and styrene oxide, yielding CO_2_-upgraded chemicals (QDO, BMO and 4-phenyl-1,3-dioxolan-2-one) with yields ranging from 64% to 93%. These results underscore the broad applicability of this *in situ* CO_2_ capture strategy for upcycling diverse PC waste without requiring high-purity raw materials.

**Figure 3.**
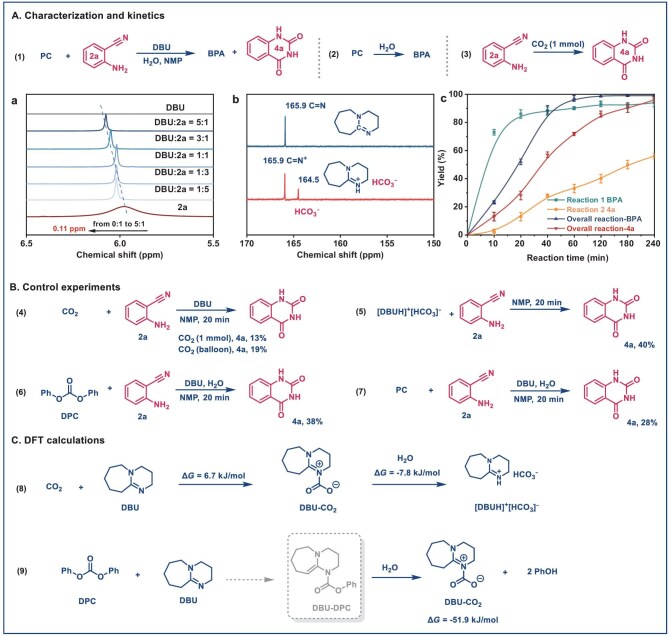
Kinetic studies and control experiments. (A) (a) ^1^H NMR spectra (600 MHz, dimethyl sulfoxide (DMSO)-*d*6, 298 K) of the DBU and **2a** with different molar ratios. (b) ^13^C NMR spectra of DBU and DBU–CO_2_ adduct in D_2_O. (c) Kinetic studies. (B) Control experiments. (C) DFT calculations.

To gain deeper insight into the reaction process and the associated interactions, we conducted a series of controlled experiments (Fig. 3, Figs S11–S13). Initially, we tested the reaction between model carbonate compounds and ABN in both the presence and absence of water. Results revealed that, without water, neither dimethyl carbonate (DMC) nor diphenyl carbonate (DPC) reacted with ABN to produce QDO, suggesting that the direct nucleophilic attack of the amine group in ABN on the carbonate group of PC does not occur during PC depolymerization (Figs S11 and S12). We analyzed the gaseous products at different reaction times and confirmed the presence of CO_2_, which was ultimately captured and fully converted into QDO (Fig. S13). This result suggested that the reaction proceeds via hydrolysis of PC. Further control experiments demonstrated that water significantly enhances the reaction between carbonate and 1,2-diaminobenzene, confirming that PC depolymerization with 1,2-diaminobenzene primarily follows a hydrolysis pathway, as illustrated in Fig. S14.

The interactions between DBU and the reactants (CO_2_ and ABN) during the CO_2_ valorization process were corroborated by NMR and FT-IR analyses (Fig. 3A-a and 3A-b, Fig. S15). The ^1^H NMR spectra demonstrated a high-field chemical shift for the hydrogen in the amine group of ABN, shifting from 5.98 to 6.09 ppm as the molar ratio of DBU to ABN decreased from 1:0 to 5:1 (Fig. 3A-a). FT-IR analysis showed attenuation in the amine peak intensity and a red shift in the C=N of DBU (Fig. S15). These results revealed the formation of a robust hydrogen bond between DBU and the amine group of ABN, which facilitates the subsequent CO_2_ capture reaction.

To further probe DBU–CO_2_ interactions, DBU was exposed to CO_2_ at room temperature (Fig. 3A-b). A new ^13^C NMR resonance at *δ* = 164.5 ppm emerges, suggesting the formation of a bicarbonate species, tentatively assigned to [DBUH]⁺[HCO_3_]^−^ in the presence of trace water [42,45,49]. These observations are in good agreement with previous reports demonstrating that DBU can effectively capture and activate CO_2_ to generate a [DBUH]⁺[HCO_3_]^−^ intermediate, which subsequently reacts with ABN to afford QDO.

Kinetic studies were performed to elucidate the time-dependent reaction behavior (Fig. 3A-c). For clarity, the system was analyzed along three pathways: (i) the overall integrated reaction; (ii) PC hydrolysis; and (iii) CO_2_ capture reaction. The results showed that PC hydrolysis occurred rapidly, achieving a 73% yield of BPA within 10 min. In contrast, under a CO_2_ dosage of 1 mmol (∼25 mL at room temperature), the yield of QDO reached only 13% after 20 min, which is markedly lower than that obtained in both the PC hydrolysis pathway and the overall reaction system (28% QDO yield). Upon increasing the CO_2_ pressure from 0.2 to 5 MPa, the QDO yield was substantially enhanced from 33% to 85%, clearly demonstrating that efficient CO_2_ conversion is highly sensitive to reactant concentration (or CO_2_ partial pressure) and thus follows a concentration-limited reaction regime (Table S4). This comparison suggests that the limited reactivity of externally supplied CO_2_ is largely associated with limited effective CO_2_ availability in the liquid phase, likely due to gas–liquid mass-transfer constraints. In contrast, employing PC as the carbon source can enhance effective local CO_2_ availability via *in situ* generation of carbonate-related species, thereby facilitating more efficient capture and conversion.

Notably, the introduction of **2a** initially suppressed the depolymerization rate of PC, yet after 60 min, it led to a higher yield than hydrolysis alone, ultimately achieving 99% yield of BPA, compared to 94% for hydrolysis. This outcome highlights that CO_2_ capture effectively shifted the reaction equilibrium toward complete depolymerization. Structurally, hydrolysis of PC (1 mmol, based on monomer units) releases an equivalent amount of CO_2_. However, introducing an equimolar amount of gaseous CO_2_ led to substantially lower ABN conversion throughout the reaction compared with using PC as the CO_2_ source. This discrepancy is further evidenced by the low yields of **4a** (13% and 19%) obtained when approximately 1 mmol of CO_2_ gas or a CO_2_ balloon was employed, respectively (Fig. 3B). In contrast, significantly higher yields were achieved using pre-formed [DBUH]⁺[HCO_3_]^−^ adducts or carbonate substrates. These observations point to the existence of a more efficient CO_2_ conversion pathway that bypasses direct activation of inert CO_2_ and instead proceeds via *in situ* interactions between carbonate substrates and DBU, generating highly reactive DBU–CO_2_ adducts or [DBUH]⁺[HCO_3_]^−^ species (e.g. DBU + DPC → DBU–DPC + phenol (PhOH) → DBU–CO_2_ + 2PhOH → [DBUH]⁺[HCO_3_]^−^).

### Mechanistic studies

**Figure 4.**
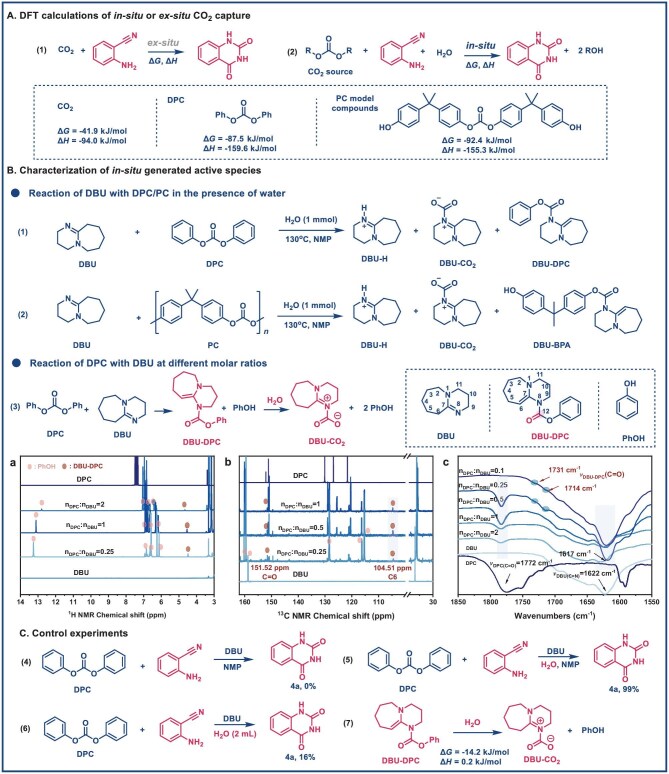
Mechanistic studies. (A) DFT calculations of reaction of **2a** with various CO_2_ sources. (B) Characterization of *in situ*-generated DBU–DPC complexes via LC-MS (see LC–MS spectra in the Supporting Information), FT-IR and NMR analysis (600 MHz, DMSO-d6, 298 K), including (a) ^1^H NMR, (b) ^13^C NMR, and (c) FT-IR spectra of the mixture of DBU and DPC. (C) Control experiments.

To validate this hypothesis, we performed density functional theory (DFT) calculations on the conversion of CO_2_ and DPC to DBU–CO_2_ or [DBUH]⁺[HCO_3_]^−^ in the presence of water. The results indicate that the interaction between CO_2_ and DBU is thermodynamically reversible, whereas the subsequent incorporation of DPC is significantly more thermodynamically favorable (Fig. 3C). We further compare the thermodynamic favorability of different CO_2_ sources by evaluating the Gibbs free energy change (Δ*G*) and enthalpy change (Δ*H*) associated with reactions involving each CO_2_ source (Fig. 4A). When CO_2_ directly participated in the reaction, Δ*G* was −41.9 kJ/mol, and Δ*H* was −94.0 kJ/mol. Substituting CO_2_ with DPC as the source reduced Δ*G* to −87.5 kJ/mol and Δ*H* to −159.6 kJ/mol. Further replacing DPC with a bisphenol A-based PC model compound yielded Δ*G* = −92.4 kJ/mol and Δ*H* = −155.3 kJ/mol. These findings indicate that *in situ* CO_2_ is thermodynamically more favorable than *ex situ* CO_2_ capture. Moreover, the BPA-based PC structure demonstrated a thermodynamic conversion advantage compared to DPC. Once initiated, the strong exothermic reaction sustains a continuous, spontaneous process, effectively reducing energy consumption. These results underscore the significant role of carbonated feedstocks in improving reaction efficiency and highlight the potential of PC as a viable CO_2_ surrogate for chemical production. This further suggests the involvement of distinct mechanisms that utilize carbonate moieties in pathways resembling *in situ* CO_2_ activation.

The formation of DBU–DPC-derived species during the reaction was systematically investigated in the presence of water. LC–MS analysis [time of flight (TOF), ES^+^ mode] conducted after 20 min under standard conditions revealed several key intermediates. Specifically, [DBUH]^+^ and DBU–CO_2_ were detected in both the DBU/DPC and DBU/PC systems, whereas DBU–DPC and DBU–BPA were observed in the DBU/DPC and DBU/PC systems, respectively (Fig. 4B, entries 1 and 2, Figs S16–S19). These results confirmed the direct interaction of DBU with carbonate-derived species and its involvement in carbonate bond activation and subsequent polymer chain transformation. The interaction between DPC and DBU at varying molar ratios was further confirmed by FT-IR and NMR analyses (Fig. 4B). New signals appeared in the ^1^H NMR spectrum at *δ* = 4.48 ppm and in the ^13^C NMR spectrum at *δ* = 104.51 ppm (C6) and *δ* = 151.52 ppm (C=O) (Fig. 4A and B, Fig. S20). In addition, FT-IR analysis revealed the appearance of distinct vibrational peaks at 1714 and 1731 cm⁻^1^, which were attributed to the C=O stretching vibrations of *V*_DBU-DPC_(C=O) (Fig. 4B-c) [43]. Distinct shifts in both DPC (C=O) and DBU (C=N) were also observed, with a blue shift from 1772 to 1782 cm⁻^1^ and a red shift from 1622 to 1617 cm⁻^1^, respectively, as the DPC:DBU ratio varied from 1:0 to 1:0.25. These results demonstrated that the DBU–DPC species could form at room temperature.

In addition, control experiments revealed that the addition of a small amount of water facilitates product formation, possibly due to the strong hydrogen-bonding ability of water, which promotes proton transfer [18]; however, excessive water hinders the effective formation of the DBU–DPC complex (Fig. 4C). Thermodynamic calculations suggested DBU–DPC to DBU–CO_2_ conversion is favorable in the presence of water, and that H_2_O can interact with DBU–CO_2_ to stabilize the intermediate (Fig. S30), indicating water not only acts as a nucleophile to initiate depolymerization but also serves as a crucial medium that facilitates the formation of DBU–CO_2_ during PC conversion. Based on these findings, we propose a DBU-mediated carbonate activation pathway involving *in situ* CO_2_ capture for the DBU-catalyzed conversion of PC into BPA and QDO (Fig. S21).

## CONCLUSIONS

This work presents a versatile *in situ* CO_2_ capture strategy for the efficient upcycling of PC to produce BPA and value-added CO_2_-upgraded products, addressing critical challenges in carbon resource loss during PC recycling. Through the reaction of PC with amine, alcohol, epoxy and alkyne as CO_2_-capturing agents, we effectively harnessed PC as a CO_2_ source, converting it into BPA and CO_2_-upgraded products (nitrogen-containing heterocyclic compounds, alkynoic acids and carbonates) via *in situ* capture. The reaction between the carbonate and 2-anthranilonitrile outperforms the direct use of an equivalent amount of CO_2_, thereby confirming the effectiveness of this strategy. Mechanistic studies suggest that the process involves a more thermodynamically favorable *in situ* CO_2_ capture pathway to generate the active DBU–CO_2_ adduct. While this strategy demonstrates broad compatibility with diverse CO_2_-trapping reagents, further progress will depend on the development of more efficient and recyclable catalytic systems to enhance turnover efficiency, long-term stability and overall process scalability. In summary, this approach offers a sustainable and economically feasible solution by transforming PC into a precursor for CO_2_-upgraded products, thereby supporting the advancement of circular economy principles and contributing to tackling the global issue of plastic waste management.

## Supplementary Material

nwag153_Supplemental_File
