# Supplementary material for: In situ CO2 capture: a general strategy for valorizing waste polycarbonate into high-value chemicals
Source: Natl Sci Rev. 2026 Mar 10;13(10):nwag153. doi: 10.1093/nsr/nwag153 (PMC13237873; doi:10.1093/nsr/nwag153)
Supplement: nwag153_Supplemental_File [file nwag153_supplemental_file.pdf]

Supporting Information

In-situ CO_2 Capture: A General Strategy to Valorize Waste Polycarbonate into High-Value Chemicals

Minghao Zhang[ab#], Shaoyu Zhang[ab#], Siming Zhu[ab#], Siyu Zhang[ab], Zexiang Wu[ab], Weixiang Wu[ab], Qingqing Mei[ab]*

[a]State Key Laboratory of Soil Pollution Control and Safety, Zhejiang University, 866 Yuhangtang Road, Hangzhou 310058, Zhejiang, China.

[b]College of Environmental and Resource Sciences, Zhejiang University, 866 Yuhangtang Road, Hangzhou 310058, Zhejiang, China.

*Corresponding author: Qingqing Mei.

[#]These authors contributed equally to this work.

Address: 866 Yuhangtang Road, Hangzhou 310058, China.

E-mail address: meiqq@zju.edu.cn

Table of Contents

1. Materials and methods 3

2. Related studies 6

3. Exploration of catalytic systems for the *in-situ* CO_2 capture strategy 12

4. Conversion of PC into BPA and benzimidazolone under solvent-free conditions 19

5. General Procedure for the Synthesis of [DBUH][OAc] and other N-containing chemicals 25

6. Control experiments and characterization 28

7. LCA and TEA analyses 41

8. ^{1}H NMR and ^{13}C NMR data of all compounds 51

9. ^{1}H NMR and ^{13}C NMR spectra of all compounds 54

10. DFT calculations 67

References 105

1. Materials and methods

1.1 Materials

PC pellets were supplied by Crestron Polymers Ltd. PC pellets were supplied by Crestron Polymers Ltd. The average size of PC was approximately 200 mesh. Analytical grade solvents and commercially available reagents were purchased from commercial sources and used directly without further purification unless otherwise stated. 2-Aminobenzonitrile (≥98%, Shang hai D&B), 2,3,4,6,7,8,9,10-octahydropyrimido(1,2-alpha)azepine (98%, Bidepharm), 2-amino-4-methylbenzonitrile (98%, Energy Chemical), 2-amino-5-methyl-benzonitrile (≥98%, Damas-beta), 2-amino-4,5-dimethoxybenzonitrile (≥98%, Damas-beta), 2-amino-5-fluorobenzonitrile (97%, Shang hai D&B), 2-amino-4-chlorobenzonitrile (99.99%, Bidepharm), 2-amino-5-chlorobenzonitrile (≥98%, Shang hai D&B), 2-amino-5-bromobenzonitrile (97%, Shang hai D&B), 1-methyl-2-pyrrolidone (99.9%, Macklin), 2,3,4,6,7,8-hexahydro-1h-pyrimido[1,2-*a*]pyrimidine (99.55%, Bidepharm), 4-dimethylaminopyridine (99%, Innochem), 1,5-diazabicyclo[4.3.0]non-5-ene (98%, Heowns), Cs_2CO_3 (99%, Innochem), K_2CO_3 (99%, Energy Chemical), Na_2CO_3 (≥99.5%, Macklin), *N*,*N*-dimethylformamide (AR, Sinopharm), toluene (AR, Sinopharm), 2-methyltetrahydrofuran (99%, Macklin), 1,4-dioxane (AR, Sinopharm), acetonitrile (AR, Sinopharm), diphenyl carbonate (99%, Meryer), 1,2-diaminobenzene (CP, Sinopharm), glacial acetic acid (AR, Sinopharm), ethyl acetate (AR, Sinopharm), 1,3,5-trioxane (＞99.5%，Macklin), DMSO-*d*6 (99%, Adamas), and deionized water were used herein.

1.2 General procedure for conversion of PC into BPA and quinazoline-2,4(1*H*,3*H*)-dione

The conversion of PC into BPA and 2-aminobenzonitrile **2a** took place in a 10 mL Schlenk tube. The PC degradation standard reaction was carried out with PC (0.254g, 1 mmol structural unit), DBU (0.4 mmol), 2-aminobenzonitrile **2a** (2 mmol) and NMP (2 mL). The Schlenk tube was heated to 130 °C (oil bath temperature) with stirring for 4 h. After completion of the reaction, the Schlenk tube was transferred to an ice water bath. After cooling to room temperature, both the yield of BPA and quinazoline-2,4(1*H*,3*H*)-dione (4a, QDO) were analyzed by ^{1}H NMR using trioxane (1 mmol) as an internal standard. If GC is employed for quantitative analysis, the acidification of the reaction mixture is required to eliminate the interference of DBU with BPA, thereby ensuring accurate quantification. The yield of BPA and **4a** were calculated using the following equation:

$$\text{Theoretically produced product amount (mol)} = \frac{\text{Quality of PC material (g)}}{0.254\ \text{g/mol}}$$

$$\text{BPA Yield (\%)} = \frac{\text{BPA amount quantified by H NMR (mol)}}{\text{Theoretically produced BPA amount (mol)}} \times 100\%$$

$$\mathbf{4a}\ \text{Yield (\%)} = \frac{\mathbf{4a}\ \text{amount quantified by H NMR (mol)}}{\text{Theoretically produced}\ \mathbf{4a}\ \text{amount (mol)}} \times 100\%$$

The depolymerization process was extended to various other commercial PC wastes (0.254 g, with impurities in the plastic itself ignored), using the same method as for the depolymerization of PC.

The separation procedures for BPA and QDO are illustrated in Figure S22.

1.3 General procedure for conversion of PC into BPA and 2-benzimidazolone

The conversion of PC into BPA and 2-benzimidazolone **6a** took place in a 10 mL Schlenk tube. The PC degradation standard reaction was carried out with PC (0.254g, 1 mmol structural unit), [DBUH][OAc] (0.4 mmol) and 1,2-diaminobenzene **5a** (2 mmol). The Schlenk tube was heated to 130 ℃ (oil bath temperature) with stirring for 4 h. After completion of the reaction, the Schlenk tube was transferred to an ice water bath. After cooling to room temperature, both the yield of BPA and 2-benzimidazolone **6a** were analyzed by ^{1}H NMR using trioxane (1 mmol) as an internal standard. The yield of BPA and **6a** were calculated using the following equation:

$$\text{Theoretically produced product amount (mol)} = \frac{\text{Quality of PC material (g)}}{0.254\ \text{g/mol}}$$

$$\text{BPA Yield (\%)} = \frac{\text{BPA amount quantified by H NMR (mol)}}{\text{Theoretically Produced BPA amount (mol)}} \times 100\%$$

$$\textbf{6a}\ \text{Yield (\%)} = \frac{\textbf{6a}\ \text{amount quantified by H NMR (mol)}}{\text{Theoretically Produced}\ \textbf{6a}\ \text{amount (mol)}} \times 100\%$$

The depolymerization process was extended to various other waste PC, employing the same method used for the depolymerization of PC pellets. Different CO_2 conversion strategies are described in Figure 2.

The separation procedures for BPA and 6a are illustrated in Figure S23.

1.4 Carbon Balance Calculation

The carbon balance of the main reaction was quantified based on the molecular compositions and experimentally measured molar amounts of all detected species. The Carbon balance of the reaction was calculated using the following equation:

$$\text{Carbon balance} = \frac{\text{n(carbon in gas products)} + \text{n(carbon in liquid products)}}{\text{n(carbon in PC input)}} \times 100\%$$

Specifically:

Catalyst	Substrate	Conversion of PC	Yield			Carbon balance
			BPA	QDO	CO_2	
DBU	PC, ABN	99%	99	96	<1%	97%

The reaction was initiated with 1.00 mmol of PC (repeating unit $(C_{16}H_{14}O_3)_n$,16 C per unit), corresponding to a total carbon input of 16.0 mmol C. After the reaction, the liquid phase contained 0.99

mmol of bisphenol A (BPA, $C_{15}H_{16}O_2$, 15 C) and 0.96 mmol of quinazoline-2,4(1H,3H)-dione (QDO, $C_8H_6N_2O_2$), in which one carbon atom originates from PC. In total, 15.81 mmol of carbon was accounted for in the liquid products. Gas-phase analysis detected only CO_2.

1.5 Characterization

^{1}H NMR and ^{13}C NMR spectra were recorded at room temperature using a Bruker Avance-600 instruments (^{1}H NMR at 600 MHz and ^{13}C NMR at 151 MHz), NMR spectra of all products were reported in ppm with reference to solvent signals [^{1}H NMR: DMSO-*d*6 (2.50 ppm), ^{13}C NMR: DMSO-*d*6 (39.52 ppm)]. Signal patterns are indicated as s, singlet; d, doublet; dd, doublets of doublet; t, triplet, and m, multiplet. The DRIFT spectra were recorded at a spectral resolution of 2 cm^{-1} using a Nicolet iS20 FT-IR spectrometer (Thermo Fisher Scientific). The surface morphology of PC bucket was characterized using cold-field scanning electron microscopy (SEM) with a Hitachi SU8010 (Japan). SEM was conducted using a Hitachi SU8010 microscope at an acceleration voltage of 3 kV. Images were captured in secondary electron mode at 500x magnifications under high vacuum conditions. The gas is detected by a SP-6800A gas chromatograph equipped with a FID detector.

2. Related studies

2.1 Related studies on hydrolysis of PC

Table S1. Comparison of PC hydrolysis strategies with different catalysts.

Entry	Catalysts	Sol.	Temp. (℃)	T (h)	Yield (%)	Ref.
1	CTAB+NaOH	-	160	10 min	Conv. > 80%	[1]
4	NaOH	1,4-dioxane	100	8	100%	[2]
5	$Fe_3O_4/SiO_2/NH_2$	DEG	160	93 min	100%	[3]
6	ZnO-NPs/Nbu	THF	100	7	99%	[4]
7	[Bmim][Cl]	-	165	3	95%	[5]
8	[Bmim][Ac]	-	140	3	96%	[6]
9	CeO_2	-	200	5	90%	[7]
10	$La(O_3SCF_3)_3$	THF	160	6	97%	[8]
11	[HDBU][LAc]	-	140	4	97%	[9]
12	Natural chlorite	THF	140	6	98%	[10]
13	Concentrated Sulfuric Acid	-	150	10	90%	[11]
14	TiO_2+NaOH	DEG	reflux	83 min	70%	[12]
15	Sub-critical water	-	280	46 min	100%	[13]
16	DBU	NMP	130	4	99	This work

The chemical recycling of PC waste has garnered significant attention as a promising approach for addressing waste accumulation. Among them, PC hydrolysis is an effective disposal method that can convert PC into BPA and CO_2. To elucidate the current challenges in hydrolysis of PC, we have conducted a comprehensive review of the relevant literature. Detailed parameters are summarized in Table S1. Our primary focus is on the catalytic systems employed in hydrolysis, revealing that most catalytic systems rely on strong acids or bases to enhance the efficiency of PC conversion. These approaches may result in the generation of acidic/alkaline wastewater during the subsequent treatment process. Notably, during the hydrolysis of PC, the CO_2 generated is often challenging to capture and convert into valuable derivatives due to its inert nature in these catalytic systems. As a result, the valorization of CO_2 is frequently overlooked in most reactions, with the primary focus on recycling BPA. This neglect leads to a significant loss of carbon resources within the plastic circular economy. However, CO_2, as a valuable C1 building block, has been successfully utilized in the production of various high-value chemicals, underscoring its potential for upcycling. Therefore, the development of efficient, recyclable, and environmentally friendly

hydrolysis systems for the complete recycling of carbon resources from PC waste, targeting both BPA production and CO_2 valorization, are urgently needed.

2.2 Typical quinazoline-2,4(1*H*,3*H*)-dione derivatives with medicinal activities

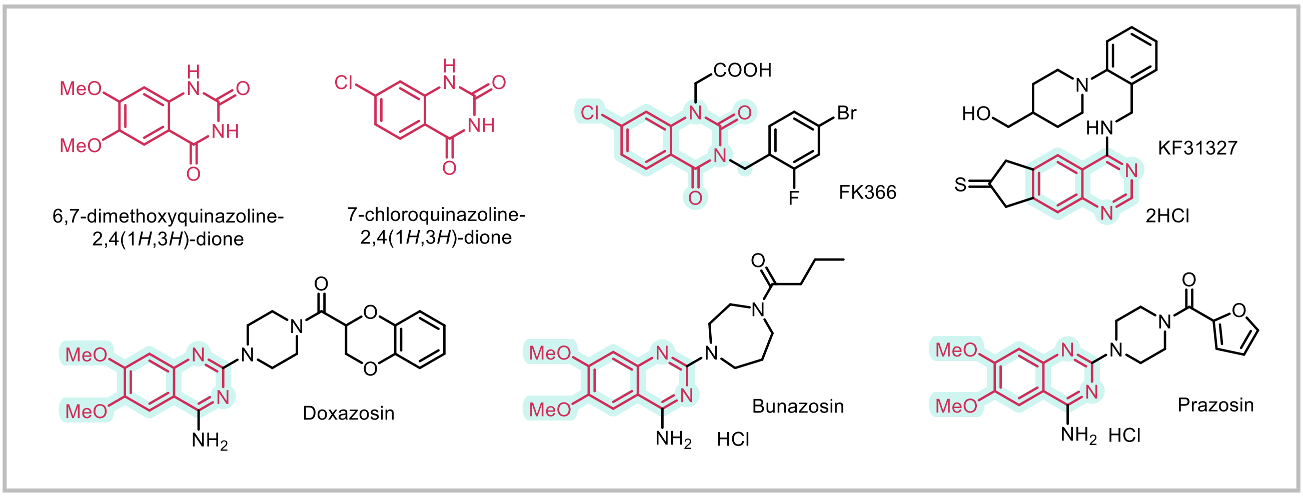

Figure S1. High-value medicinal molecules derived from 6,7-dimethoxyquinazoline-2,4(1*H*,3*H*)-dione and 7-chloroquinazoline-2,4(1*H*,3*H*)-dione.

Quinazoline-2,4(1*H*,3*H*)-dione (QDO) derivatives are a class of compounds with broad application potential across various fields due to their unique chemical structure and biological activities [14]. These valuable nitrogen-containing heterocyclic compounds have been widely used in the development of pharmaceutical agents, such as anticancer drugs and enzyme inhibitors. For example, 6,7-dimethoxyquinazoline-2,4(1*H*,3*H*)-dione and 7-chloroquinazoline-2,4(1*H*,3*H*)-dione have been employed as substrates to synthesize pharmaceutical compounds like FK366, KF31327, Doxazosin, Bunazosin, and Prazosin, each demonstrating significant activity and application potential [15, 16].

2.3 Different strategies for synthesizing QDO and its derivatives

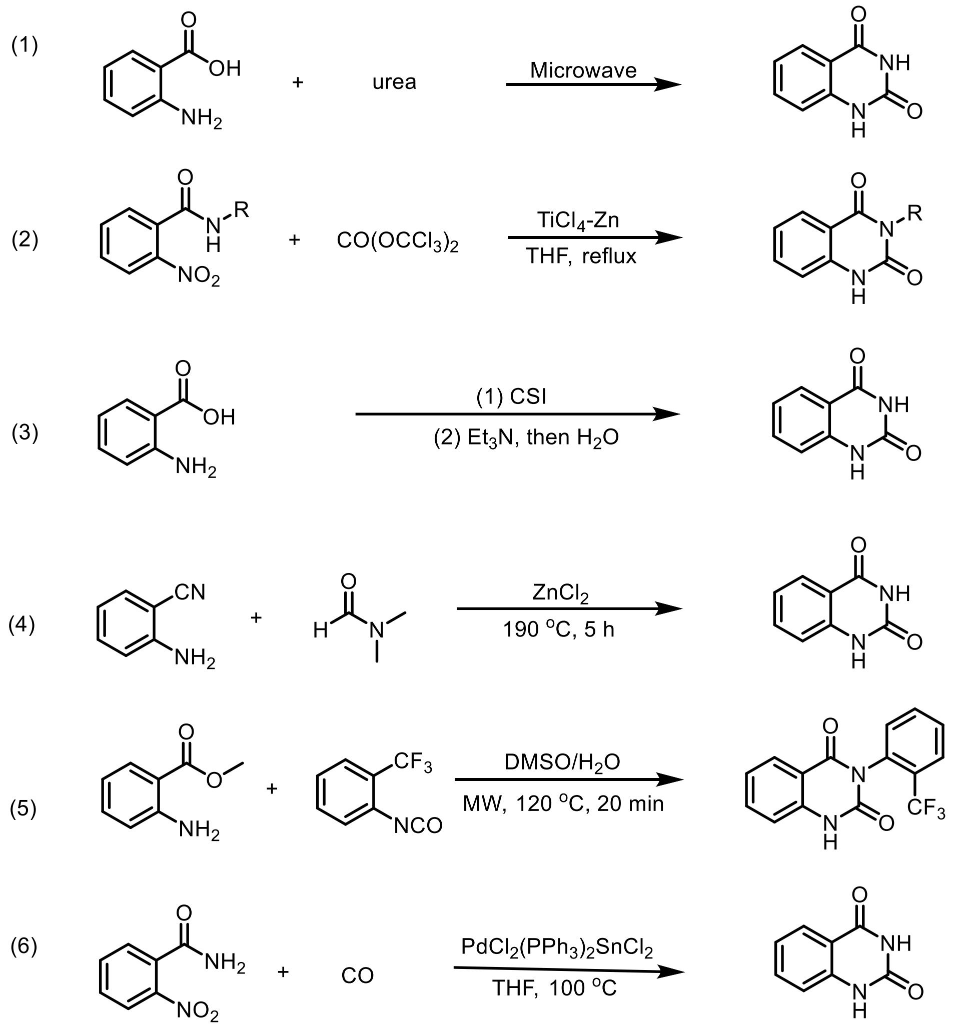

Figure S2. Synthetic route to quinazoline-2,4-dione derivatives.

Over the years, various methods for synthesizing QDO and its derivatives have been developed, typically using aromatic amines or amides as substrates in combination with other carbon sources (such as urea, phosgene, cyanate, diethylformamide, iso(thio)cyanates, and CO) to form the desired products [14, 17-22]. However, these synthetic strategies often rely on specialized and/or toxic reagents (such as phosgene), which pose significant limitations for their wider application [14]. Therefore, it is necessary to develop simple and safe methods for the synthesis of QDO and its derivatives.

2.4 Related studies on converting 2-aminobenzonitrile with CO_2 into QDO

Table S2. Comparison of the reaction of 2-aminobenzonitrile with CO_2.

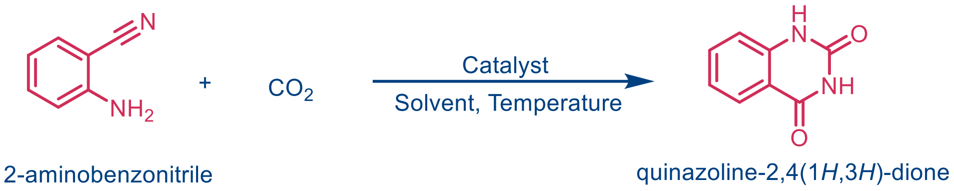

Entry	Catalysts	2-ABN: CO_2	Sol.	Temp. (°C)	T (h)	Yield (%)	Ref.
1	Cs_2CO_3	20mmol:1.3 MPa	DMF	100	4	94	[23]
2	MgO/ZrO_2	2.5mmol:3.7 MPa		130	12	92	[24]
3	$TBA_2[WO_4]$	1mmol:0.1 MPa	NMP	140	24	91	[25]
4	TBD@Fe_3O_4	0.5mmol:4 MPa	Toluene	120	16	66	[26]
5	-	5mmol:14 MPa	H_2O	160	21	92	[14]
6	DBU	10mmol:1atm	DMF	20	24	97	[27]
7	[Bmim]OH	20mmol:3 MPa	-	120	18	91	[28]
8	TMG	2mmol:10 MPa	-	120	4	89	[29]
9	NHC+K_2CO_3	2mmol:0.1 MPa	DMSO	120	8	95	[30]
10	KCC-1/HPG/Au	1mmol:1.5 MPa	H_2O	100	4min	96	[31]
11	-	10mmol:5 MPa	H_2O+DMF	150	5	99	[32]
12	[$HDBU^+$][TFE]	1mmol:0.1 MPa	-	30	24	97	[33]
13	CNFs-ADMP	5mmol:2 MPa	H_2O	100	10	91	[34]
14	Choline hydroxide	5mmol:2 MPa	H_2O	90	24	92	[35]
15	[Hmim]OH/SiO_2	1mmol:3 MPa	DMF	120	18	85	[36]
16	[Ch][Triz]	1mmol:0.1 MPa	DMF	50	24	95	[37]
17	DBU	0	NMP	130	4	96	This work

The utilization of CO_2 in combination with 2-aminobenzonitrile as a feedstock for constructing intramolecular C-O and C-N bonds in the synthesis of QDO offers favorable atom economy and environmentally friendly benefits. In recent years, various bases, ionic liquids, and solvents have been employed as facilitators to promote this conversion. However, most of these reactions suffer from the drawback of requiring increased CO_2 pressure to ensure efficient conversion. In our strategy, we propose

using PC as a CO_2 source derived from the hydrolysis of PC and employing 2-aminobenzonitrile as a trapping agent to in situ capture the CO_2 to produce QDO with high yield. This approach enables the simultaneous treatment of waste PC and the generation of both high-value BPA and nitrogen-containing products, all under ambient pressure.

3. Exploration of catalytic systems for the *in-situ* CO_2 capture strategy

3.1 Initial controlled experiments on the conversion of PC.

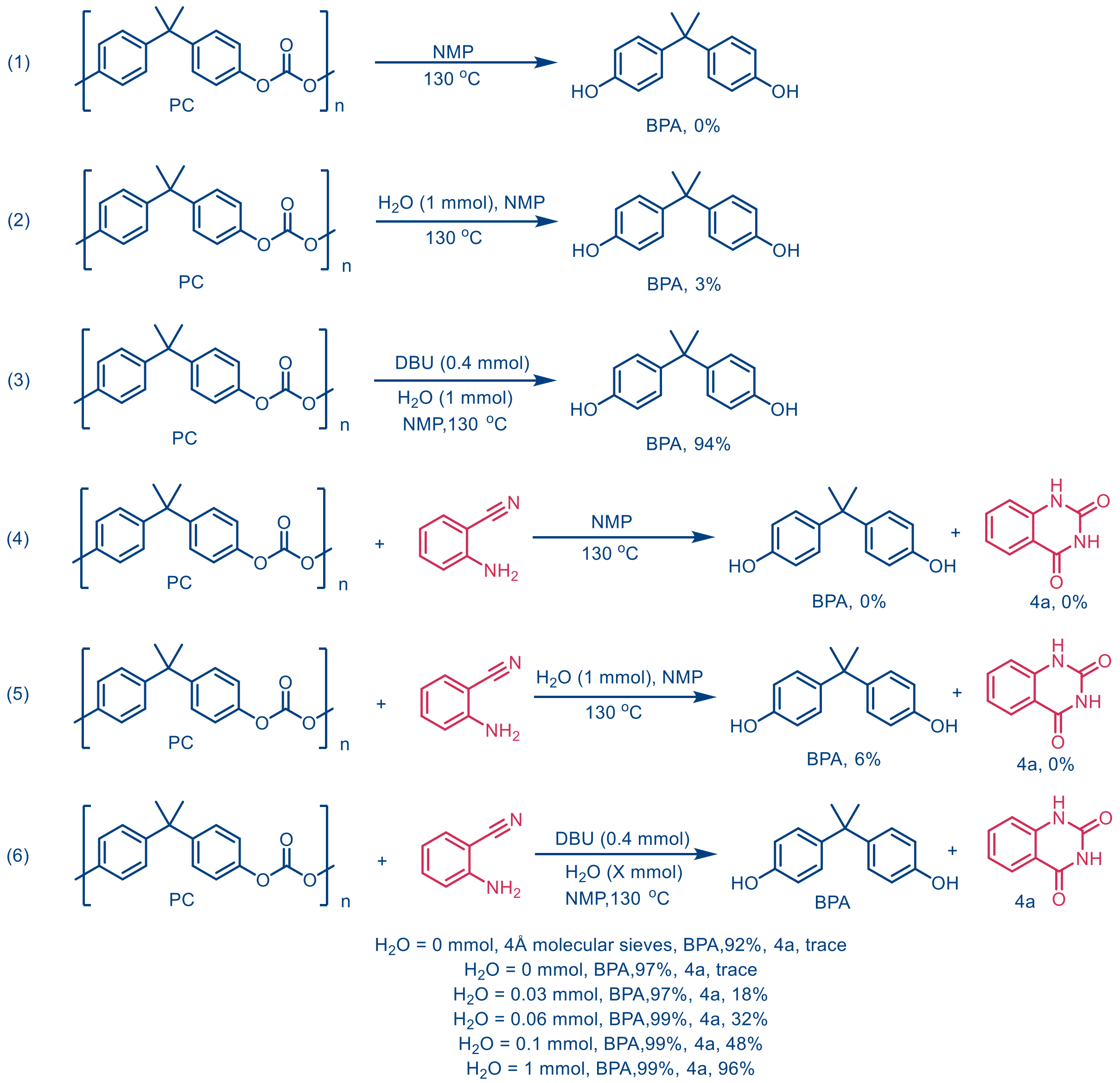

Figure S3. Controlled experiments on the conversion of PC.

We conducted a series of controlled experiments to examine the importance of various reaction parameters. Initially, PC depolymerization did not occur in the absence of both a catalyst and water, and no BPA was detected. When water was introduced into the reaction system, PC hydrolysis was minimal, yielding only trace amounts of BPA. With the addition of DBU as a catalyst, DBU demonstrated excellent catalytic activity for PC hydrolysis, achieving a BPA yield of 94%. When 2-aminobenzonitrile was included in the reaction system, the depolymerization of PC similarly did not occur in the absence of both

water and catalyst. Although the addition of water slightly induced PC hydrolysis, the process remained incomplete and inefficient.

Subsequently, control experiments were performed to elucidate the role of trace water in the reaction system. Both the dehydrated system using 4 Å molecular sieves and the untreated reaction mixture enabled efficient depolymerization of PC into BPA, which was isolated after post-reaction acidification with hydrochloric acid. In sharp contrast, QDO was scarcely detected under these water-deficient conditions. Notably, upon gradually increasing the water content from 0.03 to 1 mmol by reintroducing deionized water into the dehydrated solvent, the yield of QDO increased dramatically from 18% to 96%. These results demonstrate that water plays a decisive role in steering the reaction pathway toward QDO formation. Together, these findings reveal that the cooperative presence of both the catalyst and trace water is essential for enabling the *in-situ* CO_2 capture strategy.

3.2 Screening of experimental parameters of the conversion of PC.

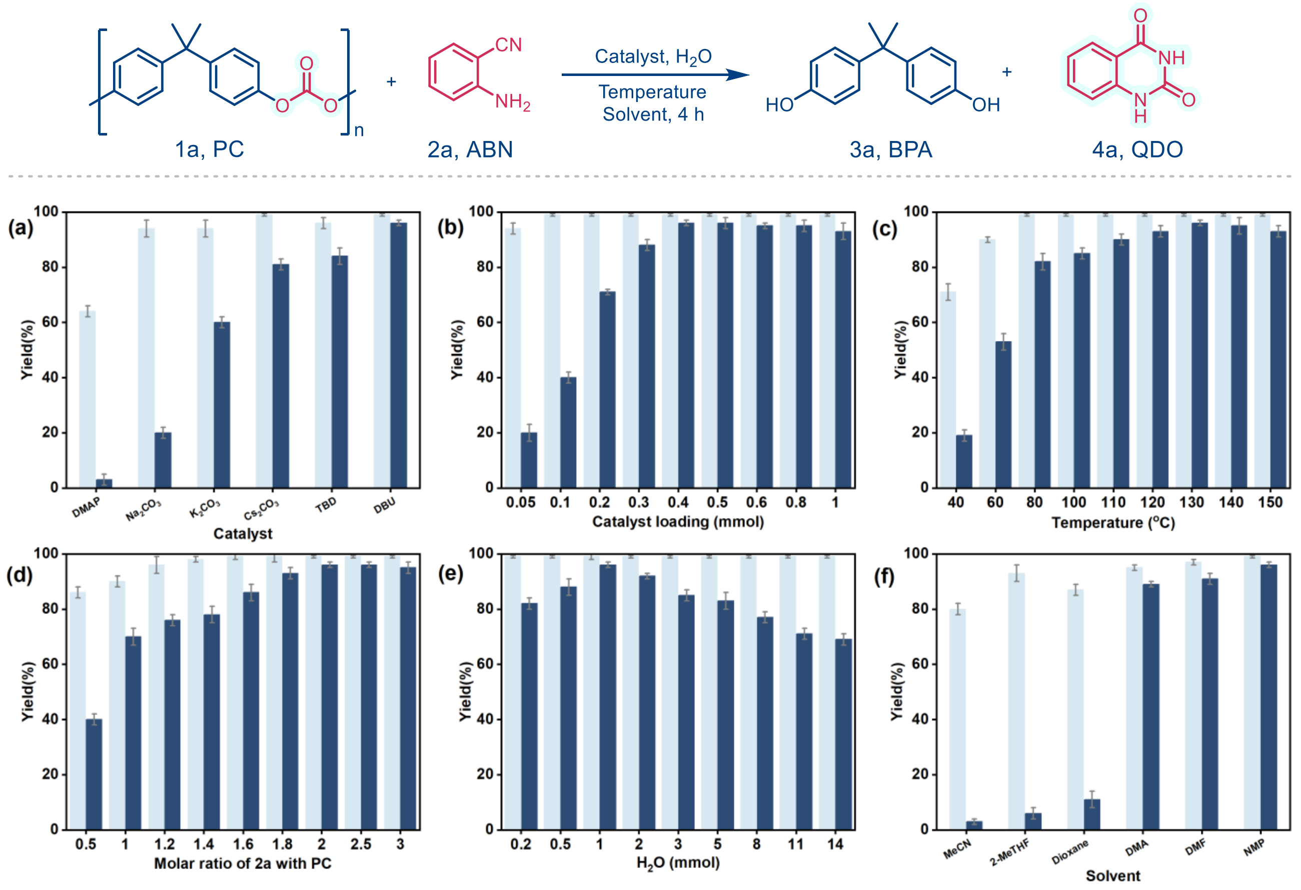

Figure S4. Screening of experimental parameters. Standard reaction conditions: PC (1 mmol, based on monomer), DBU (0.4 mmol), ABN (2 mmol) and NMP (2 mL) at 130 °C for 4 h. Yield was analyzed by ^{1}H NMR (600 MHz, DMSO-*d*6, 298 K) with trioxane as internal standard.

Various organic and inorganic bases were tested as catalysts for the *in-situ* capturing of CO_2 with ABN during the hydrolysis of PC. The addition of basic catalysts facilitated both the hydrolysis of PC and the subsequent CO_2 valorization, achieving yields ranging from 64-99% for BPA and 3-98% for QDO. Notably, only DBU and TBD exhibit superior catalytic efficiencies, likely due to their ability to readily form a stable adduct structure with CO_2 [38-41]. The effect of DBU dosage was further investigated, revealing that increasing the catalyst amount had a minimal impact on the reaction. Conversely, insufficient catalyst quantities led to incomplete hydrolysis of PC and inefficient CO_2 valorization, thereby reducing the yields of both BPA and QDO. Reaction temperature was identified as another critical factor influencing PC depolymerization. At temperatures above 80 °C, the conversion of PC was largely

unaffected, whereas lowering the temperature below 80 °C resulted in incomplete depolymerization and diminished yields of BPA and QDO. The amount of water used in the hydrolysis process was also examined. Insufficient water led to incomplete hydrolysis of PC, while excessive water negatively impacted QDO yield. Additionally, the quantity of ABN significantly influenced the efficiency of *in-situ* CO_2 capture and subsequent conversion to QDO. Increasing the ABN amount to 2 equivalents markedly improved QDO production, achieving complete conversion of PC into BPA and QDO with yields of 99% and 96%, respectively (Figure 2 and S5). Finally, the effect of solvents (MeCN, DMF, DMA, 2-MeTHF, and dioxane) was evaluated. While these solvents had minimal impact on PC hydrolysis, they proved inadequate for efficient CO_2 valorization, particularly when non-amide solvents were used. Based on the above optimization, the optimal reaction conditions were determined as follows: PC (0.254 g, 1 mmol structural unit), ABN (2a, 1 mmol), DBU (0.4 mmol), H_2O (1 mmol), and NMP (2 mL), reacted at 130 °C for 4 h.

3.3 ^{1}H NMR analysis of reaction mixture of PC with 2-aminobenzonitrile

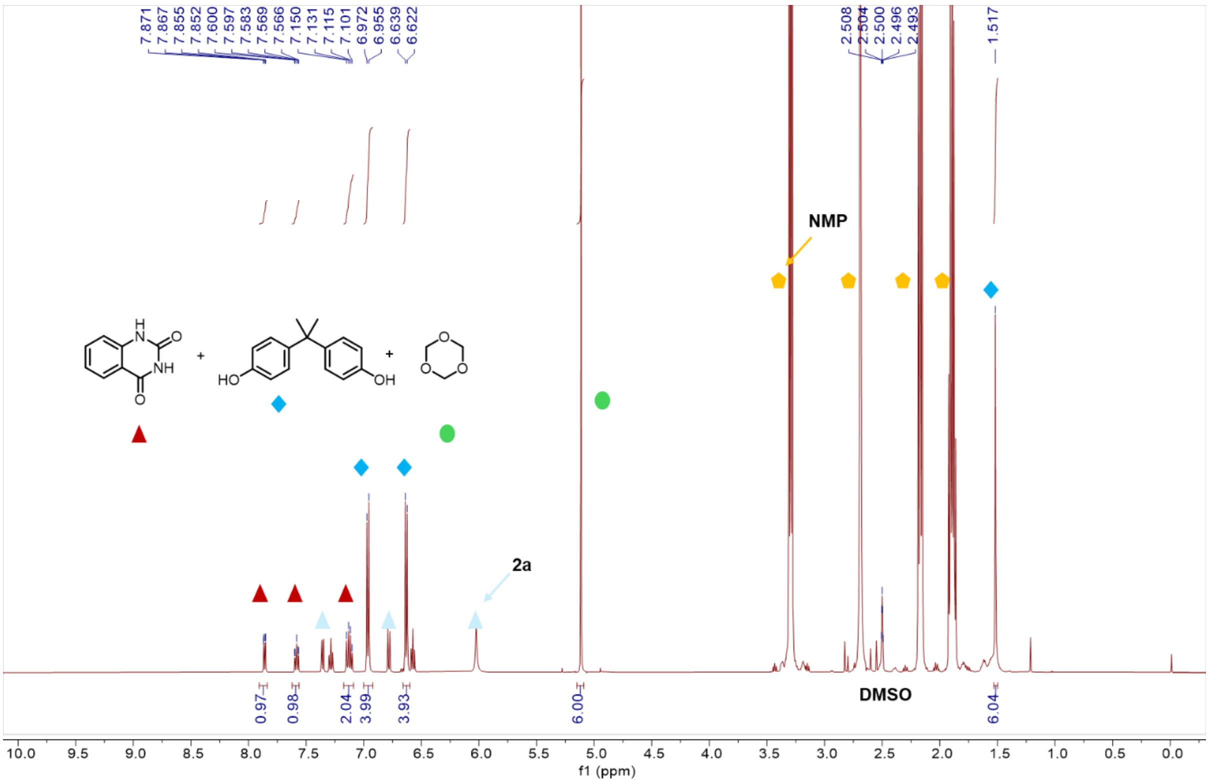

Figure S5. ^{1}H NMR spectrum (600 MHz, DMSO-*d*6, 298 K) of reaction mixture with trioxane as internal standard.

After the reaction was completed, we analyzed the product yields via ^{1}H NMR. The ^{1}H NMR spectrum indicated that PC (0.258 g, 1.01 mmol, based on monomer) was fully converted into BPA and QDO, with yields of 99%±1% and 96±2%, respectively. During the reaction, the produced CO_2 effectively reacted in situ with 2-aminobenzonitrile to produce QDO. The product mixture contained only unreacted 2-aminobenzonitrile and the high-value products (BPA and QDO), without any by-products derived from 2-aminobenzonitrile.

3.4 Experimental procedure for the separation of QDO

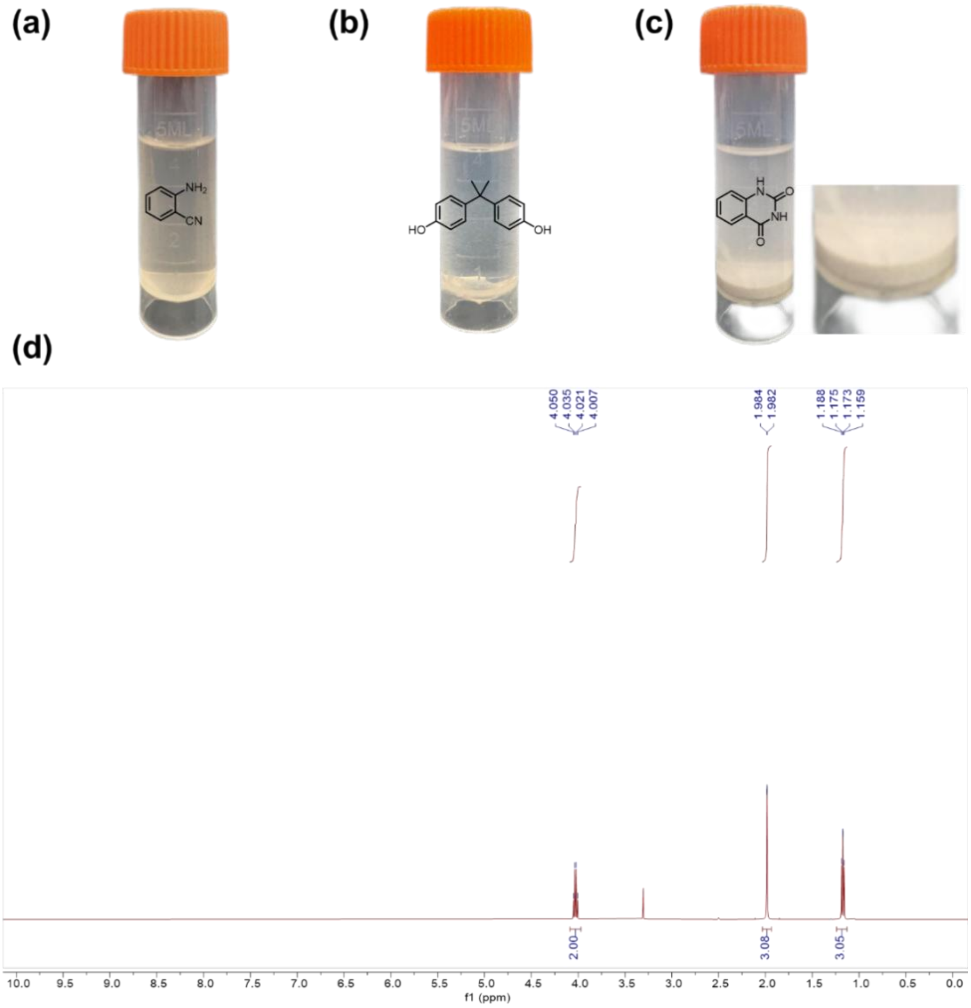

Figure S6. Solubility testing of products and raw materials in ethyl acetate: (a) 2-anthranilonitrile (b) BPA (c) QDO (d) ^{1}H NMR analysis of the reaction mixture of QDO with ethyl acetate.

To isolate and purify QDO, we conducted solubility tests on 0.2 g of the starting material (2-aminobenzonitrile) and products (BPA and QDO) in ethyl acetate. The results showed that 2-aminobenzonitrile and BPA were completely soluble in ethyl acetate, while QDO remained nearly insoluble. The added grayish-white solid, QDO, precipitated at the bottom of the plastic test tube. Further analysis by ^{1}H NMR confirmed that only ethyl acetate and DMSO-*d*6 were present in the mixed solution, with QDO remaining undissolved. This method can therefore be used to effectively separate QDO.

3.5 Characterization of BPA and QDO via monitoring of their characteristic peaks during the reaction

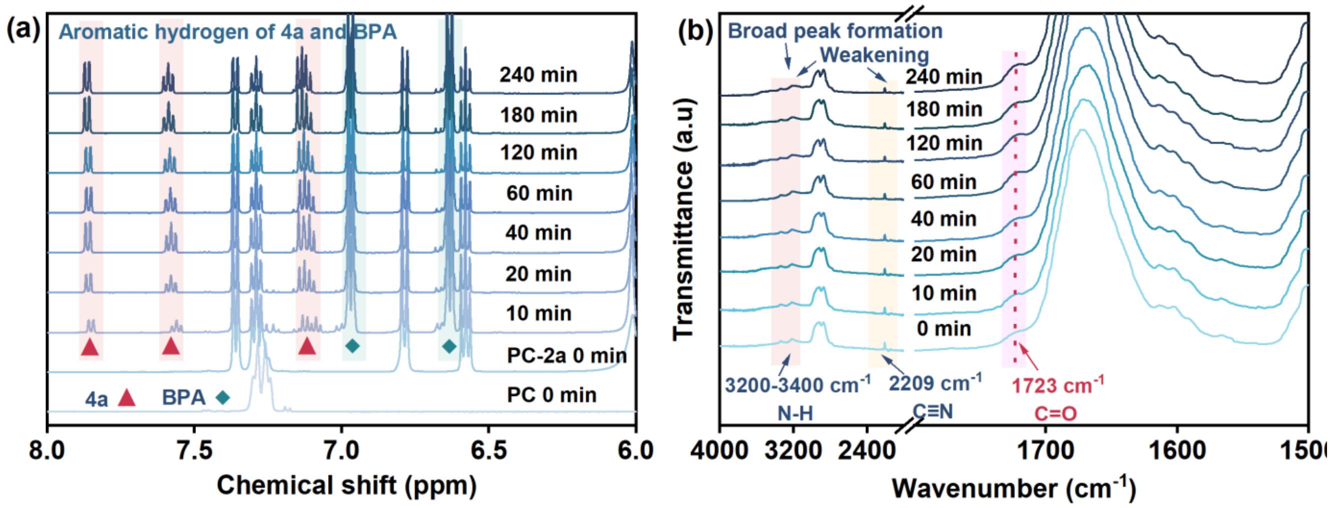

Figure S7. The changes in product peak signals during the reaction were characterized by (a) ^{1}H NMR and (b) FT-IR analysis.

The ^{1}H NMR results indicated that the aromatic characteristic peaks of both BPA and QDO gradually intensified, accompanied by a weakening of the aromatic characteristic peak of PC (Figure S7a). As shown in Figure 4b, the peaks at 3200-3400 cm^{-1} and 2209 cm^{-1} are attributed to the stretching vibrations of -NH_2 and -CN groups, respectively [42]. As the reaction proceeds, these peaks gradually broaden, and their intensities progressively weaken, indicating that the amino groups of ABN are being progressively converted into amide groups in QDO. The peak at 1723 cm^{-1} is attributed to the stretching vibration of the C=O ketone carbonyl group in QDO [43, 44] (Figure S7b). The gradual increase in peak intensity further confirmed the substantial formation of QDO as the reaction progresses.

4. Conversion of PC into BPA and benzimidazolone under solvent-free conditions

4.1. Exploration of catalytic systems and reaction conditions

Table S3. Optimization of the conversion of PC into BPA and 2-benzimidazolone

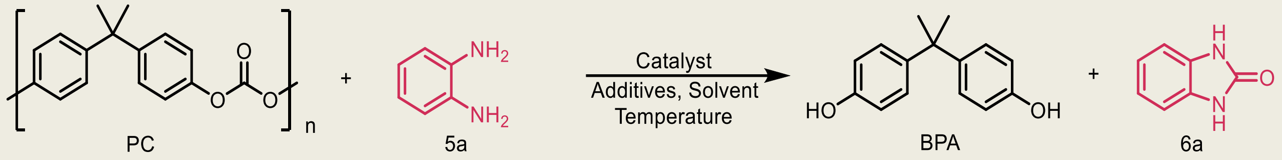

Entry	Catalyst	loading	H_2O (mmol)	Solvent	BPA Yield (%)[a]	6a Yield (%)[a]
1	-		1	NMP	28	26
2	DBU	40 mol%	1	NMP	99	48
3	[DBUH][OAc]	40 mol%	1	NMP	99	58
4	[DBUH][OAc]	40 mol%	-	NMP	99	54
5	[DBUH][OAc]	40 mol%	1	-	99	90
6	[DBUH][OAc]	40 mol%	0.5	-	99	91
7	[DBUH][OAc]	40 mol%			99	97
8	[DBUH][OAc]	50 mol%	-	-	99	95
9	[DBUH][OAc]	30 mol%	-	-	99	97
10	[DBUH][OAc]	20 mol%	-	-	99	90
11	[DBUH][OAc]	10 mol%	-	-	90	70
12	[DBUH][OAc]	5 mol%	-	-	60	55
13	[DBUH][OAc]	20 mol%	-	-	72[b]	65[b]
16	[DBUH][OAc]	20 mol%	-	-	99[c]	97[c]
17	[DBUH][OAc]	20 mol%	-	-	99[d]	94[d]
18	[DBUH][OAc]	20 mol%	-	-	99[e]	96[e]

Catalytic system exploration. [a] Standard reaction conditions: PC (1 mmol, based on monomer), 1,2-diaminobenzene (2 mmol), and [DBUH][OAc] (40 mol%) at 130 °C for 4 h. Yields were determined by ^{1}H NMR analysis with trioxane as internal standard. [b] 2a (1 mmol). [c] 2a (3 mmol). [d] at 120 °C. [e] at 140 °C.

To further obtain different types of high-value nitrogen-containing heterocyclic compounds through the *in-situ* CO_2 capture strategy, we selected 1,2-diaminobenzene **5a** as the CO_2 capture reagent to test

the conversion of PC into BPA and 2-benzimidazolone (**6a**, BMO). Initially, in the absence of a catalyst, the conversion of PC was incomplete, resulting in yields of 28% and 26% for BPA and BMO, respectively. When DBU was used as a catalyst, PC underwent efficient hydrolysis, resulting in a 97% yield of BPA, but the yield of 6a was only 48%. To enhance the yield of BMO while ensuring efficient hydrolysis of PC, we synthesized DBU-based ionic liquids ([DBUH][OAc]) to increase the reactivity of 1,2-diaminobenzene [45, 46]. Under the catalysis of [DBUH][OAc], the yield of BMO further increased to 58%. We then removed solvents or additives to ensure effective contact between [DBUH][OAc] and **5a**. After the removal of additives and solvents, PC underwent efficient conversion, with yields of BPA and BMO reaching 99% and 95%, respectively. Subsequently, catalyst loading adjustments were also conducted. In this study, a reduction in catalyst loading did not significantly impact the conversion rate of PC to BPA; however, it did result in a lower yield of BMO. Temperature adjustments revealed that while an increase had minimal influence on the reaction, reducing the temperature led to incomplete conversion of PC. Through systematic optimization, we identified the ideal 1,2-diaminobenzene-involved PC-CO_2 valorization reaction parameters: PC (1 mmol), **5a** (2 mmol), and [DBUH][OAc] (0.4 mmol) at 130 °C for 4 hours, performed under solvent-free catalytic conditions.

4.2 ^{1}H NMR analysis of reaction mixture of PC with 1,2-diaminobenzene

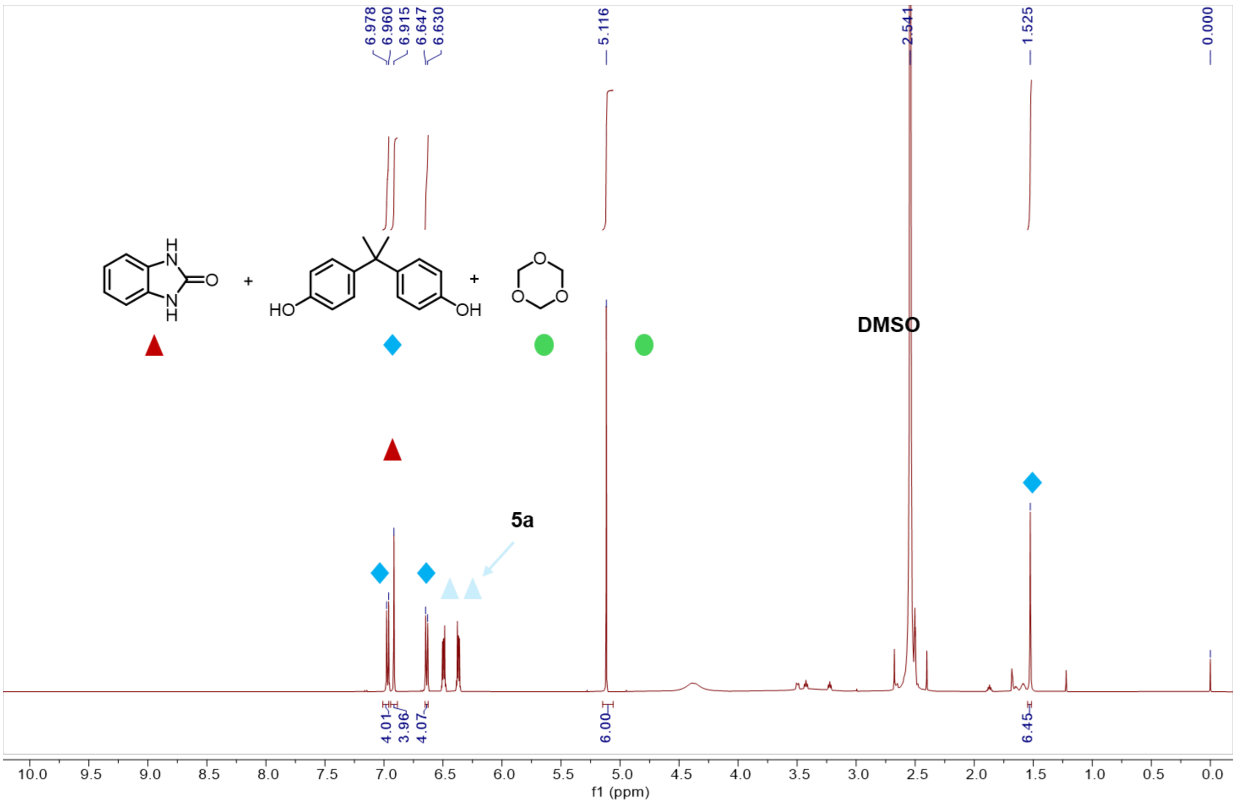

Figure S8. ^{1}H NMR spectrum (600 MHz, DMSO-*d*6, 298 K) of reaction mixture with trioxane as internal standard.

After the reaction was completed, we analyzed the product yields via ^{1}H NMR. As shown in Figure S8, the ^{1}H NMR spectrum indicated that PC (0.259 g, 1.02 mmol, based on monomer) was fully converted into BPA and 2-benzimidazolone, with yields of 99%±1% and 97%±1%, respectively. These results demonstrated the excellent catalytic performance of [DBUH][OAc] in the solvent-free reaction of PC with 1,2-diaminobenzene. The product mixture contained only unreacted 1,2-diaminobenzene and the high-value products (BPA and BMO), with no by-products derived from 1,2-diaminobenzene.

4.3 Converting commercial PC waste into BPA and QDO

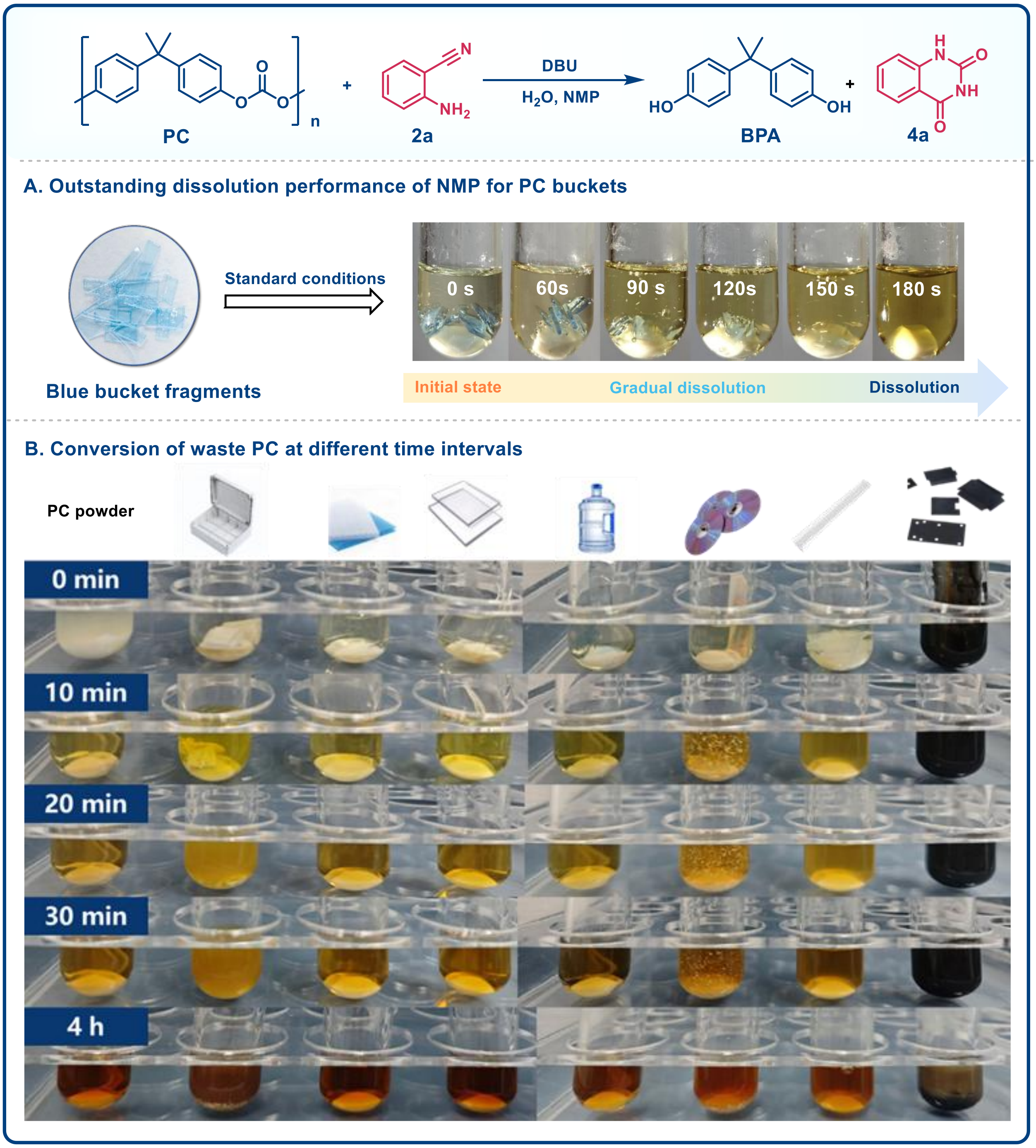

Figure S9. Images depicting the conversion of PC to BPA and QDO at different time intervals.

To evaluate the applicability of this strategy for the treatment of commercial waste PC plastics, we have depicted the changes in various types of PC plastics (hollow sheets, optical disks, transparent plates, lamp tubes, electric enclosures, black insulation sheets, and blue water buckets) within the reaction system at different time intervals in the images. During the reaction, all types of PC waste plastics were effectively

dissolved within 10-30 minutes. After four hours of reaction, some of the reaction mixtures exhibited insoluble solids (electric enclosures and black insulation sheets), which may be additives or impurities from the plastics themselves. Experimental results confirmed that the catalytic system effectively converted PC waste plastics into BPA and QDO, demonstrating its potential for handling various types of PC waste.

4.4 SEM characterization of the change process of plastic disks in NMP.

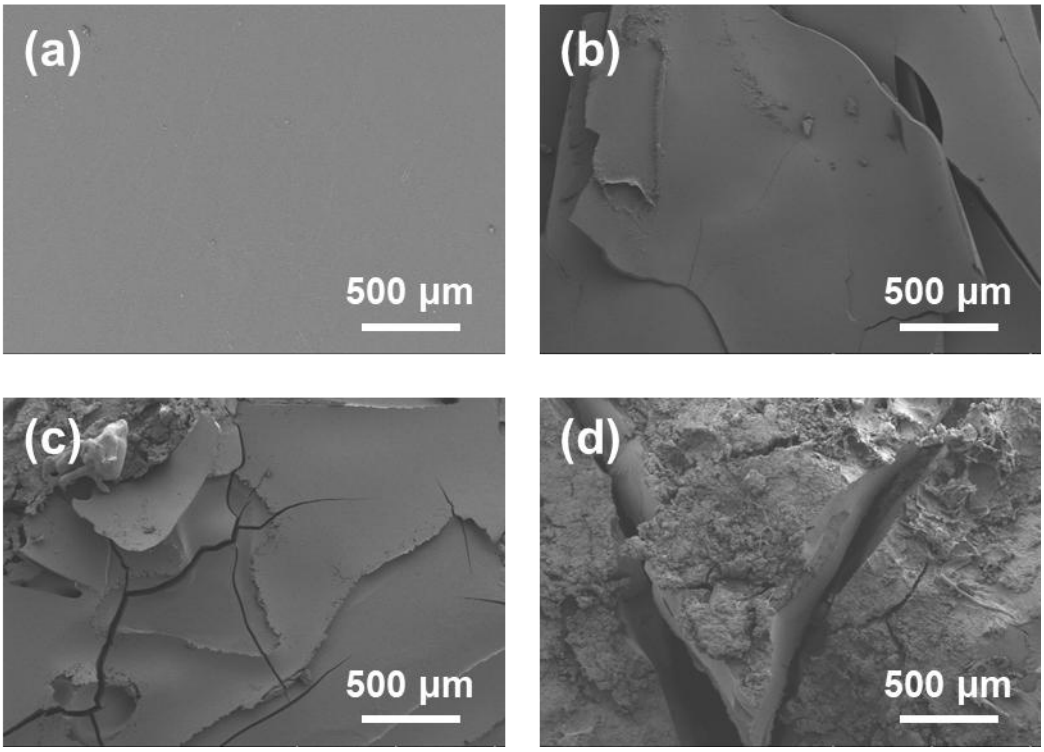

Figure S10. SEM images of various PC disks treated with NMP for different durations. (a) raw PC disks (b) 5 min (c) 10 min (d) 20 min.

Scanning electron microscopy (SEM) analysis was performed on the raw PC disks and their counterparts treated at 130 °C for different times (5 min, 10 min and 20min). The SEM image showed that the raw PET bottle sample has a smooth and flat structure. After NMP treatment, the PC disks exhibit significant changes: the previously smooth and flat structure is replaced by a concave and convex morphology characterized by cracks. This phenomenon gradually became obvious over time. This structural change can be attributed to the good solubility of PC in NMP with increasing temperature, facilitating the PC depolymerization.

5. General Procedure for the Synthesis of [DBUH][OAc] and other N-containing chemicals

5.1 General procedure for the synthesis of [DBUH][OAc]

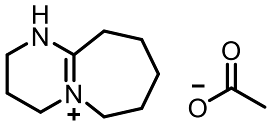

[DBUH][OAc] was synthesized according to the relevant. DBU (5 mmol) was placed in a 50 mL two-neck flask equipped with an ice-water bath under nitrogen atmosphere. Acetic acid (5 mmol) was then added dropwise with continuous stirring. The mixture was stirred at 50 °C for 24 hours, resulting in a light yellow, viscous oily liquid. The product was subsequently dried under vacuum at 80 °C for 24 hours. The product was analyzed by ^{1}H NMR and the data were agreement with the reported spectra data of [DBUH][OAc].

[DBUH][OAc]: little yellow liquid; ^{1}H NMR (600 MHz, DMSO-*d*6) δ = 3.51-3.49 (m, 2H), 3.43 (t, *J* = 8.4 Hz, 2H), 3.22 (t, *J* = 7.2 Hz, 2H), 2.72 (t, *J* = 6.6 Hz, 2H), 1.89-1.84 (m, 2H), 1.68-1.63 (m, 5H), 1.60-1.57 (m, 4H); ^{13}C NMR (151 MHz, $CDCl_3$) δ = 173.9, 165.2, 53.1, 47.8, 37.6, 31.2, 28.4, 26.3, 24.8, 23.7, 19.2.

5.2 *N*-alkylation reaction of quinazoline-2,4(1*H*,3*H*)-dione

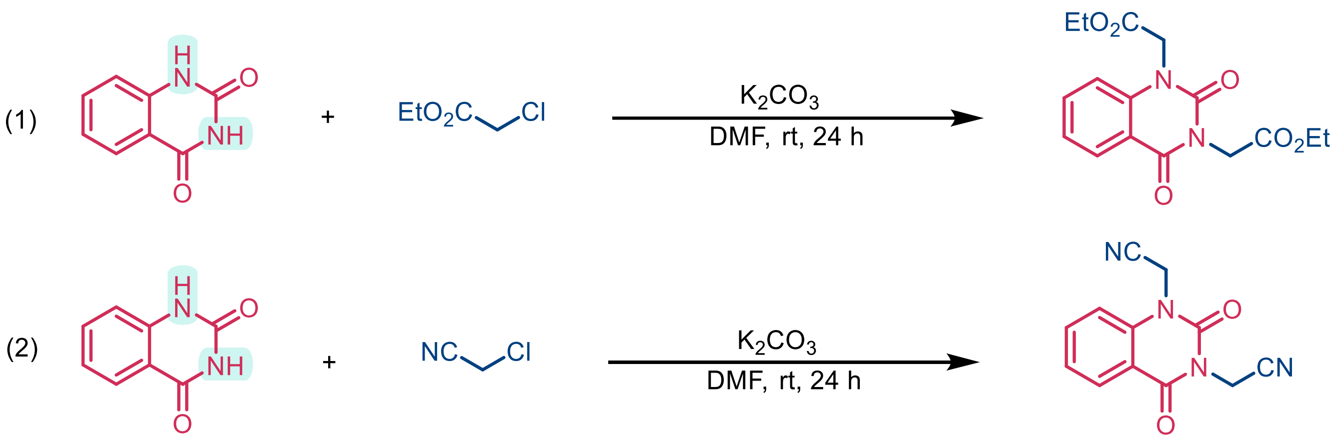

N-alkylated quinolinedione derivatives were synthesized according to the relevant. A mixture of quinazoline-2,4(1*H*,3*H*)-dione (6.2 mmol), K_2CO_3 (12.4 mmol) and DMF (10 mL) were added to a round bottom flask and stirred for 15 minutes at room temperature. Then *N*-alkylation regents (ethyl chloroacetate or chloroacetonitrile, 12.4 mmol) were added to the reaction mixture and stirred for 24 hours at room temperature. Upon completion of the reaction, the reaction mixture poured into ice water, and the crude solid product was obtained by filtration. Subsequently, the crude product was washed with deionized water and dried at 80 °C to give the corresponding *N*-alkylated quinolinedione derivatives.

5.3 Chlorination of quinazoline-2,4(1*H*,3*H*)-dione and 2-benzimidazolone with $POCl_3$.

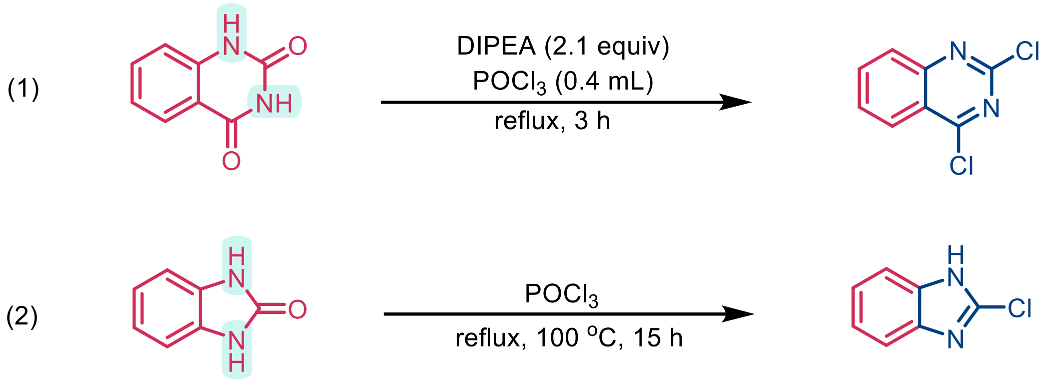

2,4-Dichloroquinazoline was synthesized according to the relevant. literature. A mixture of quinazoline-2,4(1*H*,3*H*)-dione (6.17 mmol), DIPEA (12.9 mmol) and $POCl_3$ (0.4 mL) were added to a polytetrafluoroethylene rotor (25 mL), which was then hermetically sealed within a stainless-steel reactor. The reactor was stirred at reflux for 3 hours with a rotational speed of 500 r/min on the heating plate. Upon completion of the reaction, the reaction mixture was carefully poured over crushed ice and stirred vigorously. This aqueous mixture was then extracted with dichloromethane (DCM). Subsequently, the combined organic phases were washed with brine, dried over anhydrous Na_2SO_4, and concentrated under reduced pressure. The crystalline solid obtained was further dissolved in DCM, after which it was filtered through a silica pad using DCM as the eluent. The removal of the organic phase yielded 2,4-dichloroquinazoline.

2-Chloro-1*H*-benzo[*d*]imidazole was synthesized according to the relevant. A mixture of 1,3-dihydro-2*H*-benzo[*d*]imidazol-2-one (3 mmol) and $POCl_3$ (3 mL) were added to a polytetrafluoroethylene rotor (25 mL), which was then hermetically sealed within a stainless-steel reactor. The reactor was stirred at 100 °C for 3 hours with a rotational speed of 500 r/min on the heating plate. Upon completion of the reaction, the reaction mixture was carefully poured over crushed ice and stirred vigorously. And the pH of the mixture was adjusted to pH = 7 using NaOH (6M). Subsequently, 2-chloro-1H-benzo[*d*]imidazole was obtained after filtration and washing with deionized water.

6. Control experiments and characterization

6.1 The effect of water on the conversion of carbonate and 2-aminobenzonitrile to QDO

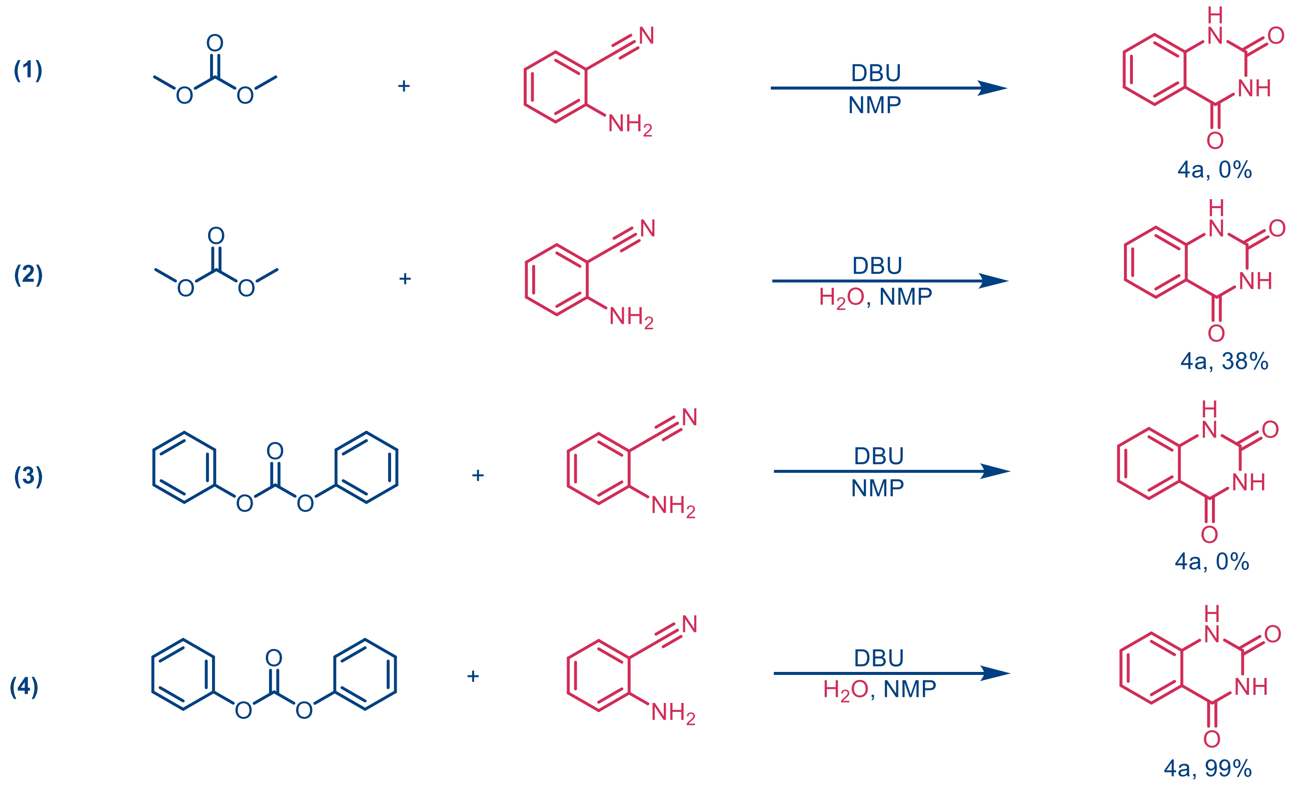

Figure S11. Control experiments on the conversion of carbonate and 2-aminobenzonitrile.

To verify whether the initial depolymerization step of PC occurs via nucleophilic attack by the amine group in 2-aminobenzonitrile on the carbonate group of PC or through hydrolysis of PC releasing CO_2, we used carbonate compounds (DMC and DPC) as model compounds to study their conversion. The results, shown in Figure S11, revealed that in the absence of water, no conversion of DMC and DPC occurred, and no QDO was detected. However, upon the introduction of water into the reaction system, the yield of QDO significantly increased, with dimethyl carbonate and diphenyl carbonate converting to QDO at yields of 38% and 99%, respectively. These results suggested that the presence of water may trigger the hydrolysis of carbonate compounds, producing CO_2, which is then captured in-situ by 2-aminobenzonitrile, rather than through nucleophilic attack by the amine group on the carbonate. Therefore, it can be inferred that the conversion of PC to BPA and QDO is triggered by hydrolysis.

6.2 Collection of the generated gas through a gas bag

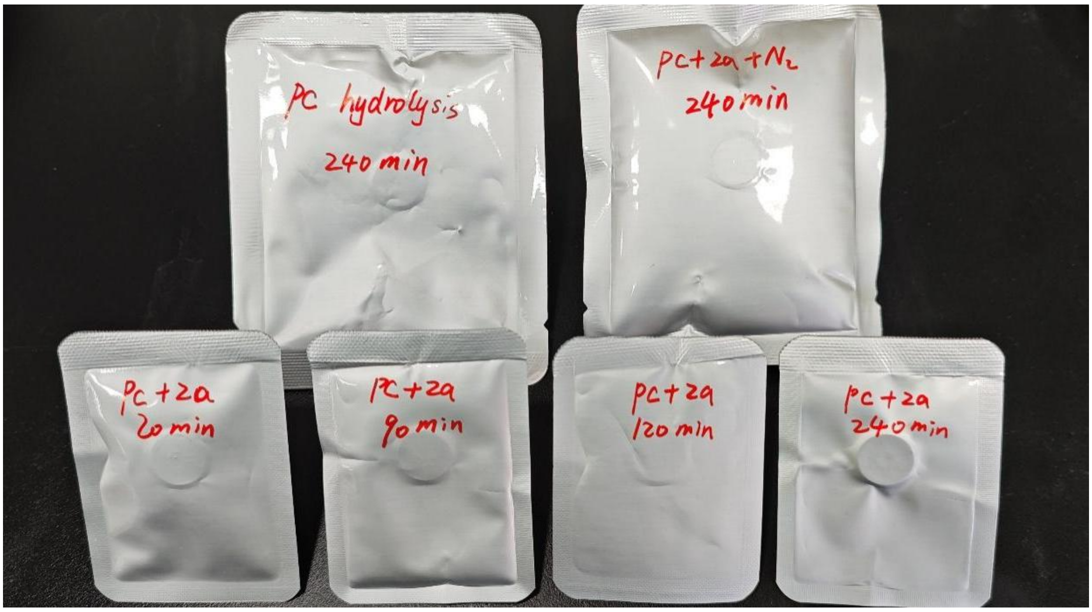

Figure S12. Gas collection during the hydrolysis of PC and the reaction of PC with 2-aminobenzonitrile.

We performed gas collection during the hydrolysis of PC and the reaction of PC with 2-aminobenzonitrile. After 240 minutes of PC hydrolysis, the gas bag was noticeably inflated, indicating the presence of a large volume of gas. In contrast, the gas collected during the reaction of PC with 2-aminobenzonitrile at different time intervals showed significantly less gas than during PC hydrolysis. Moreover, as the reaction time increased, the gas volume gradually decreased and eventually the gas bag collapsed.

6.3 Detection of released CO_2 during the reaction process

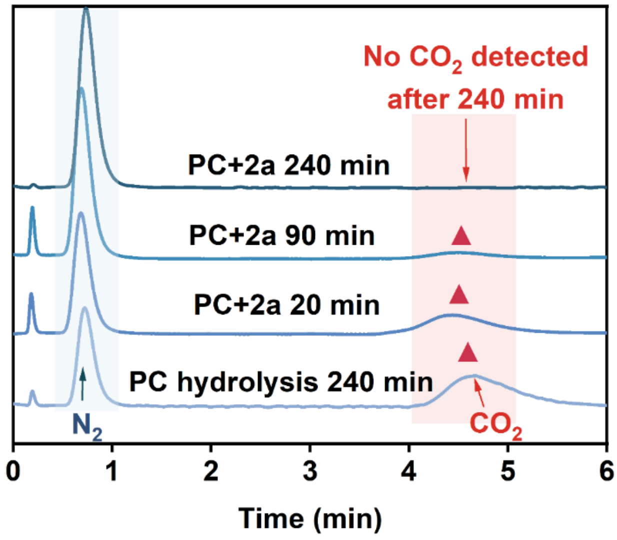

Figure S13. CO_2 detection during the PC hydrolysis and the reaction of PC with 2a.

We further performed CO_2 detection during the reaction process and found that the hydrolysis of PC under the promotion of NMP effectively generates CO_2. In contrast, during the reaction of PC with 2-aminobenzonitrile, the CO_2 peak generated is lower than that observed during the hydrolysis of PC, and it is rapidly consumed until it becomes undetectable. This suggests that the carbon resources from the PC carbonate units are efficiently utilized and undergo effective conversion within the reaction system.

6.4 The effect of water on the conversion of carbonate and 1,2-diaminobenzene to BMO

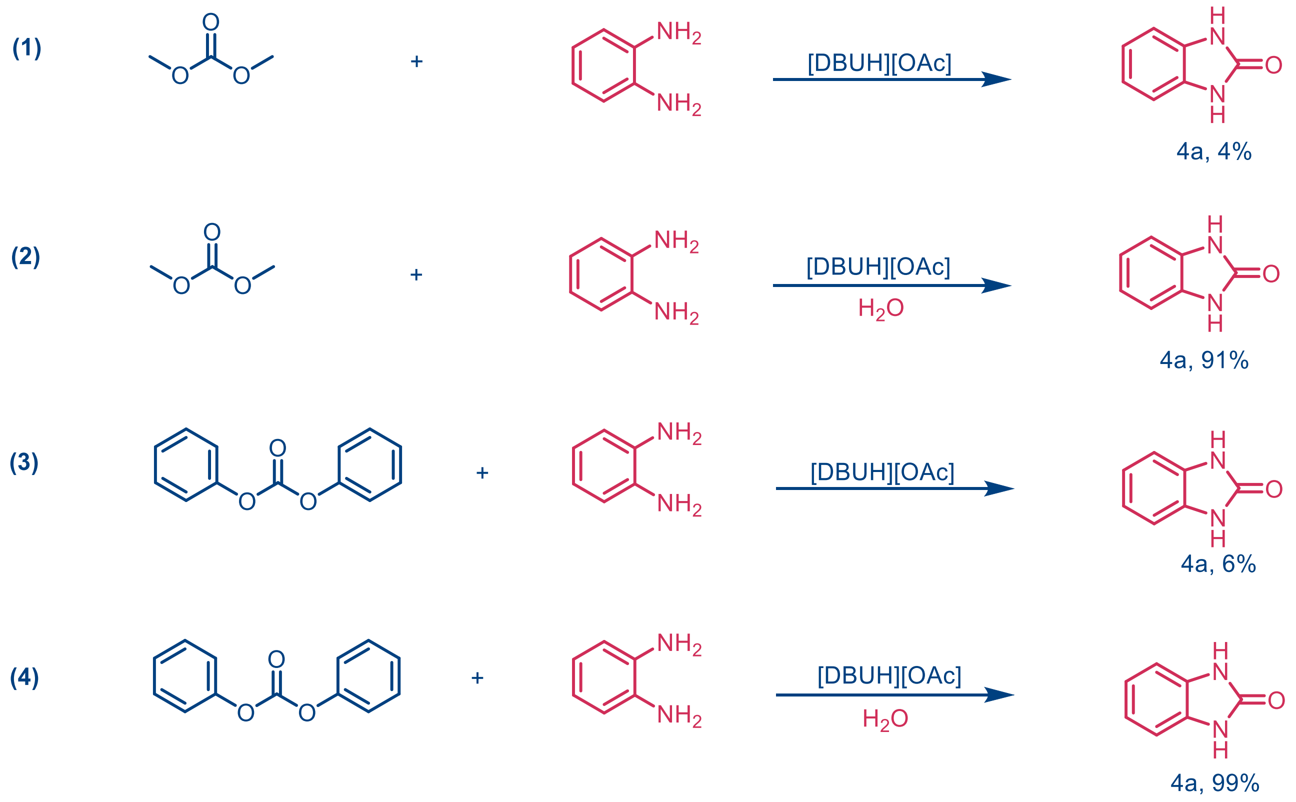

Figure S14. Control experiments on the conversion of carbonate and 1,2-diaminobenzene.

To verify whether the initial depolymerization step of PC occurs via nucleophilic attack by the amine group in 1,2-diaminobenzene on the carbonate group of PC or through hydrolysis of PC releasing CO_2, we also conducted control experiments using carbonate as feedstock. Unlike with 2-aminobenzonitrile as the reactant, in the absence of water, DMC and DPC were found to convert to small amounts of BMO with yields of 4% and 6%, respectively. This result indicated that direct nucleophilic attack by 1,2-diaminobenzene on the carbonate was inefficient. However, when water was introduced into the reaction system, the conversion of both carbonate compounds to 2-benzimidazolone was significantly enhanced, yielding 91% and 99%, respectively. This result suggested that the presence of water promoted the hydrolysis of carbonates, releasing CO_2, which is then captured by 1,2-diaminobenzene and efficiently converted to BMO under the catalysis of [DBUH][OAc]. Based on these results, it can be inferred that the reaction of PC with 1,2-diaminobenzene to form BPA and BMO may proceed via two possible pathways: (1) nucleophilic attack by the amine group in 1,2-diaminobenzene on the carbonate, or (2)

hydrolysis of PC, which triggers the initial depolymerization. The above results suggested that the reaction predominantly proceeds via the latter pathway, where the hydrolysis of PC is the primary mechanism.

6.5 Characterization of the interaction between DBU and 2-aminobenzonitrile by FT-IR analysis

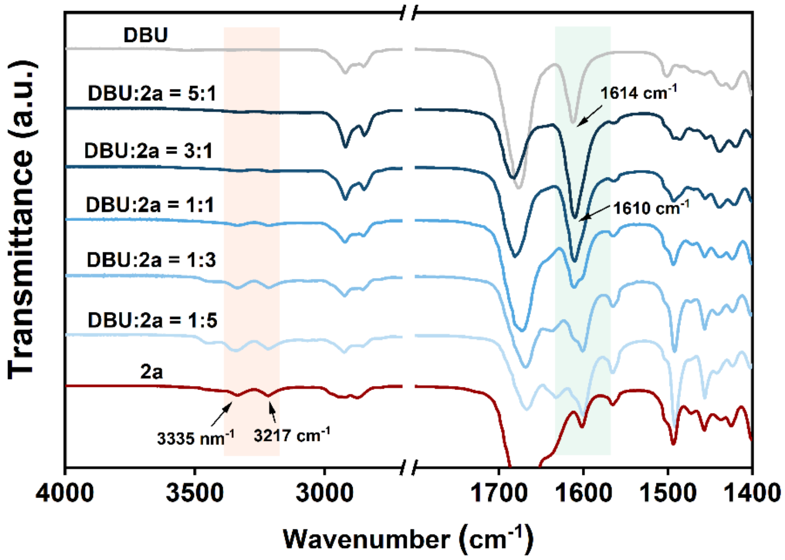

Figure S15. Control experiments on the conversion of carbonate and 2-aminobenzonitrile.

To further investigate the interaction between DBU and 2-aminobenzonitrile, FT-IR analyses were conducted on mixtures with varying molar ratios of DBU and 2a. This interaction was evidenced by a noticeable attenuation of the N–H stretching band of the amine group and a red shift of the C=N of DBU stretching vibrations from 1614 cm^{-1} to 1610 cm^{-1} [42, 47].

6.6 Reaction kinetics under different CO_2 pressures

Table S4. Conversion of 2a to QDO under different CO_2 pressures.

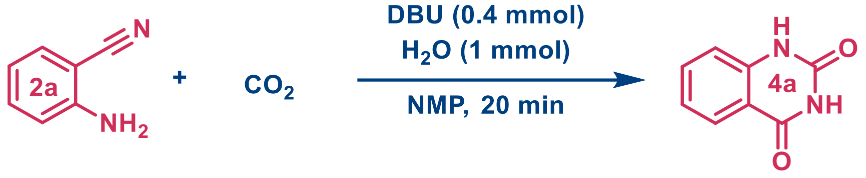

Entry	CO_2 pressure	catalyst	BPA Yield	QDO Yield
1	PC 1mmol	DBU	52%	28%
2	1 mmol	DBU	-	13%
3	0.2 MPa	DBU	-	33%
4	0.6 MPa	DBU	-	36%
5	1 MPa	DBU	-	50%
6	1.5 MPa	DBU	-	73%
7	2 MPa	DBU	-	80%
8	3 MPa	DBU	-	85%
9	4 MPa	DBU	-	87%
10	5 MPa	DBU	-	86%

Catalytic system exploration. Standard reaction conditions: 2a (1 mmol), DBU (0.4 mmol), H_20 (1 mmol) and NMP (3 mL) at 130 °C for 20 min. Yields were determined by ^1H NMR analysis with trioxane as internal standard.

Under standard reaction conditions, reactions of 2a were conducted under different CO_2 pressures. Using 1 mmol of PC as the CO_2 source, a QDO yield of 28% was obtained after 20 min, which is significantly higher than that achieved with 1 mmol of gaseous CO_2 (13%). Moreover, increasing the CO_2 pressure from 0.2 to 5 MPa led to a gradual increase in the QDO yield from 33% to 86%, demonstrating the pronounced impact of CO_2 concentration on the reaction outcome.

6.7 LC–MS characterization of active species formed during the reaction of DBU with DPC/PC in the presence of water.

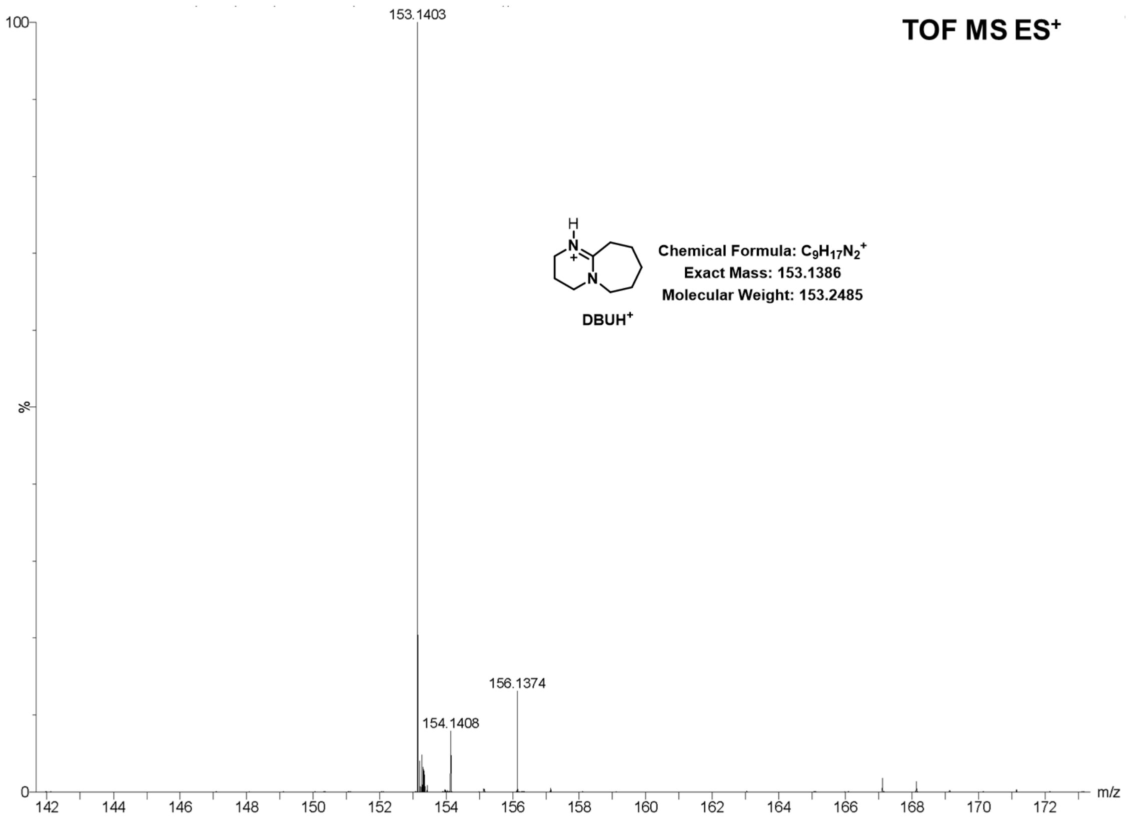

Figure S16. LC–MS characterization of $DBUH^+$.

LC–MS analysis (TOF, ES^+ mode) was performed for both the DBU/model compound DPC system and the DBU/real plastic PC system after reaction at 130 °C for 20 min in NMP with water as an additive. In both systems, several key intermediates were consistently detected, including $[DBUH]^+$, DBU–CO_2, DBU–DPC (DPC system), and DBU–BPA (PC system), providing direct evidence for the active involvement of DBU in carbonate bond activation and its interaction with both model and polymeric substrates.

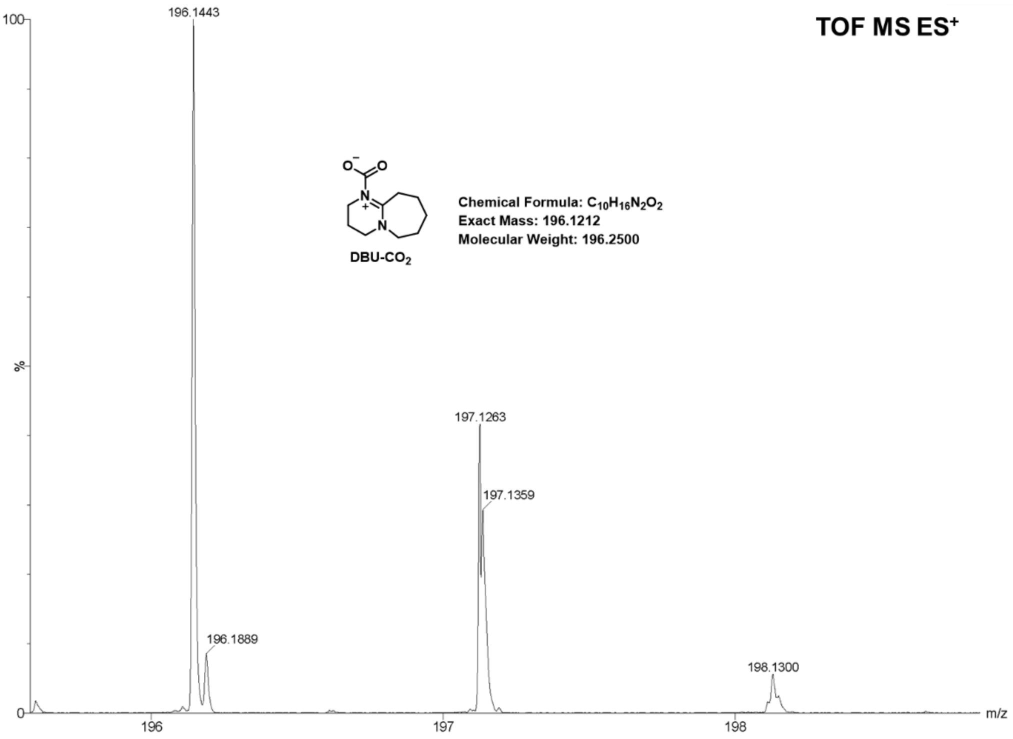

Figure S17. LC–MS characterization of DBU-CO_2.

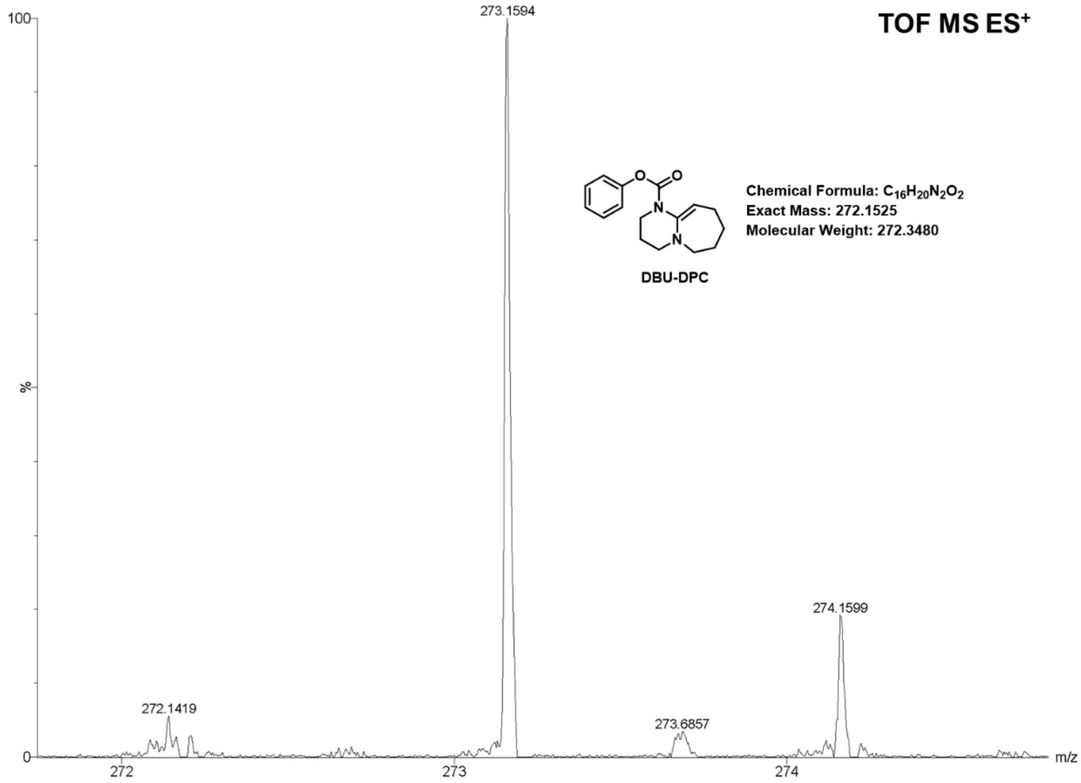

Figure S18. LC–MS characterization of DBU-DPC.

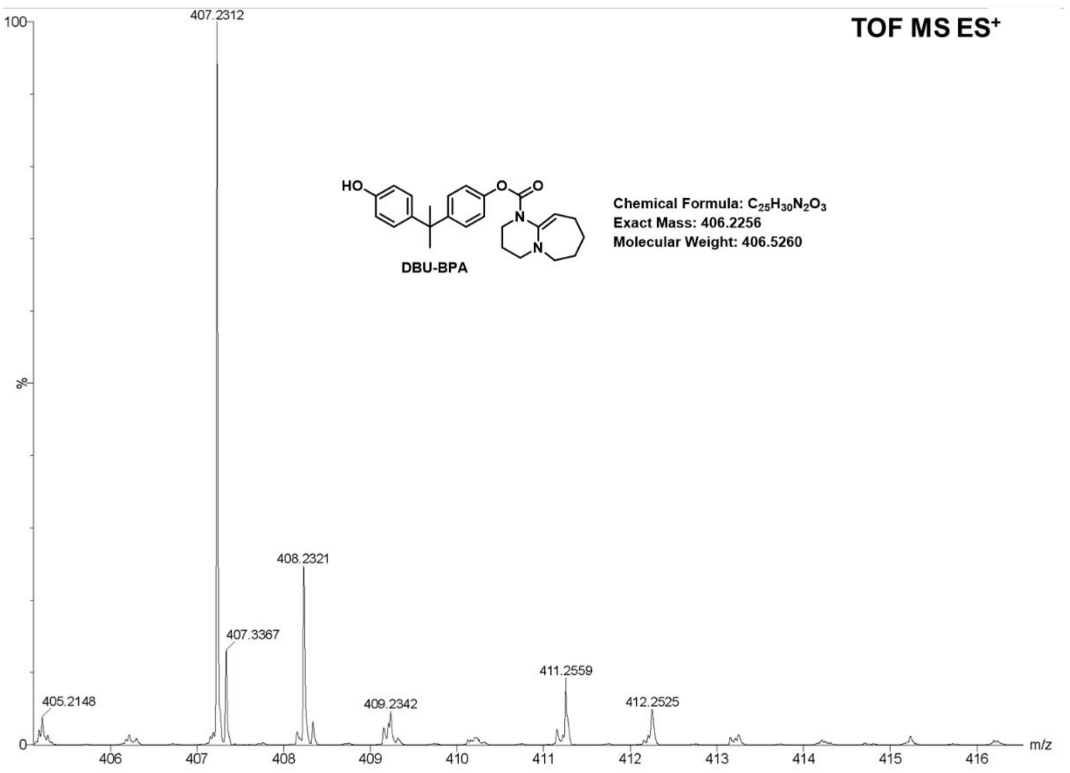

Figure S19. LC–MS characterization of DBU-BPA.

6.8 Characterization of effective interactions between DBU and DPC

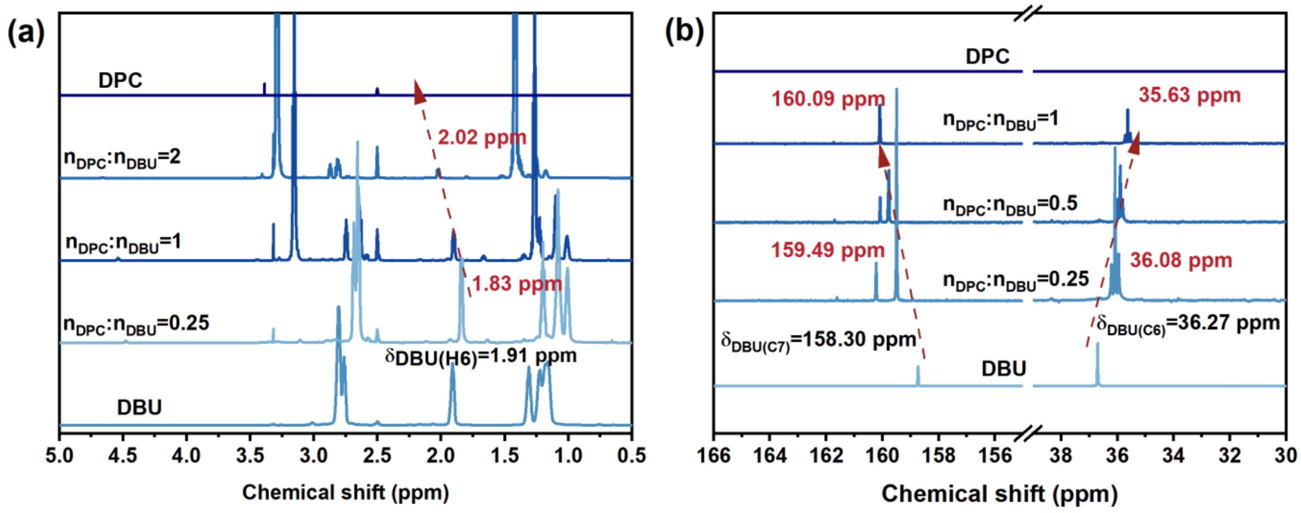

Figure S20. Effective interaction between DBU and DPC.

The interaction between DPC and DBU was confirmed through NMR analysis of the reaction mixture at different ratios after 6 hours at room temperature. The results confirmed that as the ratio of DPC to DBU increased from 0:1 to 2:1, the proton (H6) signal of DBU at shifted from 1.83 ppm to 2.02 ppm in ^{1}H NMR spectrum. ^{13}C NMR spectrum further validated the efficient interaction between DPC and DBU, with the C7 signal of DBU shifting from 158.30 ppm to 160.09 ppm, and the C6 signal shifting from 36.27 ppm to 35.63 ppm.

6.9 ^{1}H NMR characterization of hydrogen bond interactions between [DBUH][OAc] and 1,2-diaminobenzene

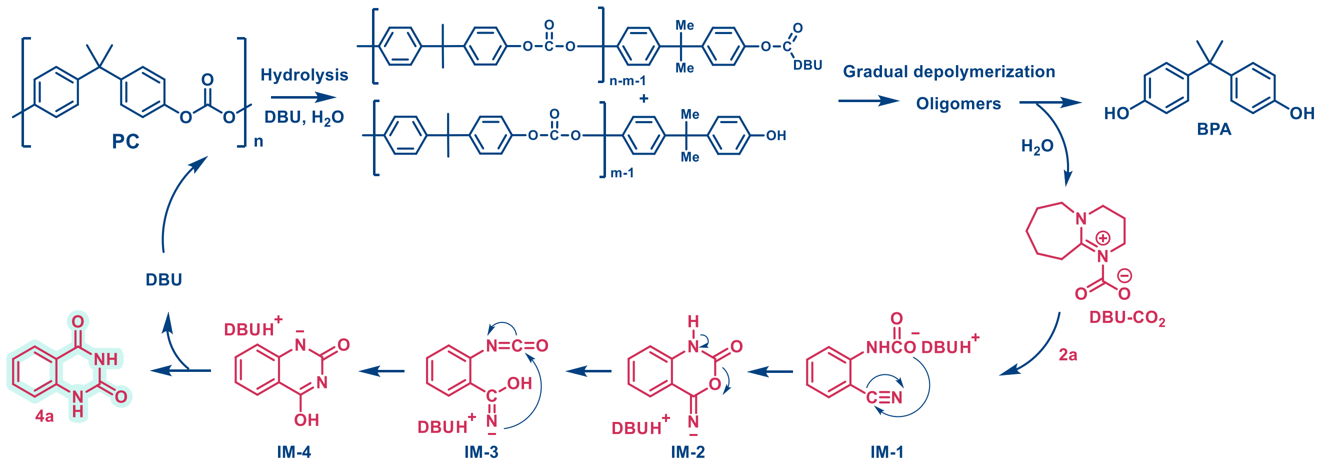

Figure S21. The possible reaction mechanism of converting PC into BPA and QDO.

Based on experimental results and relevant literature, a plausible reaction mechanism for the conversion of PC with 2-aminobenzonitrile into BPA and QDO is illustrated in Figure S17. Initially, DBU reacts with PC and is incorporated into the polymer chain, forming an R–CO_2–DBU intermediate. This process triggers the depolymerization of PC, leading to its gradual breakdown into smaller oligomers. Subsequently, under the influence of water, the R–CO_2–DBU intermediate undergoes hydrolysis, and the resulting DBU–CO_2 adduct is readily captured by 2-aminobenzonitrile with the assistance of DBU and H_2O, forming a carbamate salt intermediate (IM-1) via intermolecular carbonylation. This is followed by a feasible intramolecular nucleophilic cyclization, resulting in the formation of IM-2. Subsequently, IM-2 rearranges through an isocyanate intermediate (IM-3), yielding IM-4. Finally, IM-4 undergoes stabilization to produce the value-add nitrogenous QDO.

7. LCA and TEA analyses

7.1 Simple separation procedure for QDO and BPA

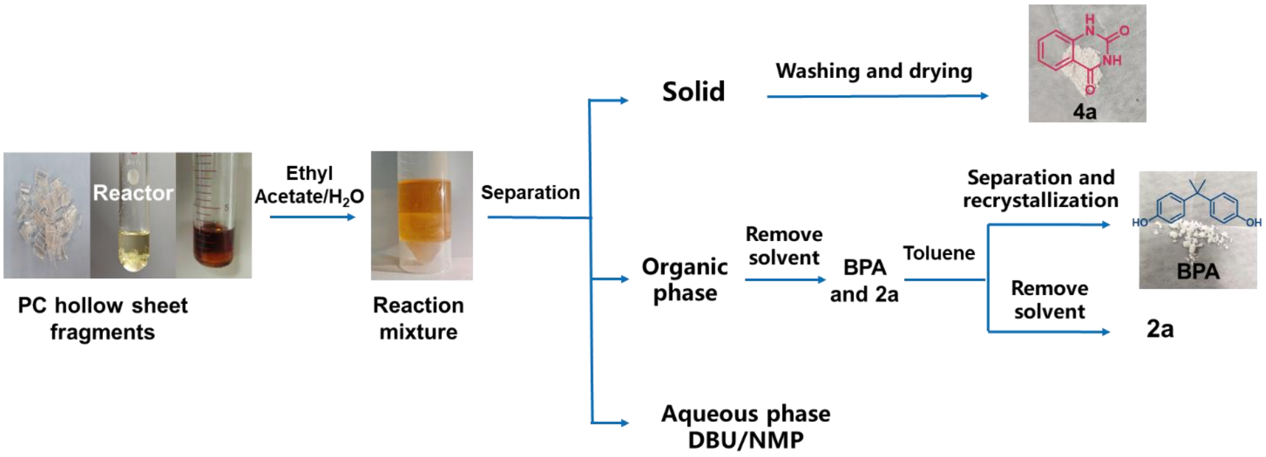

Figure S22. Simple separation procedure for QDO and BPA.

7.2 Simple separation procedure for BMO and BPA

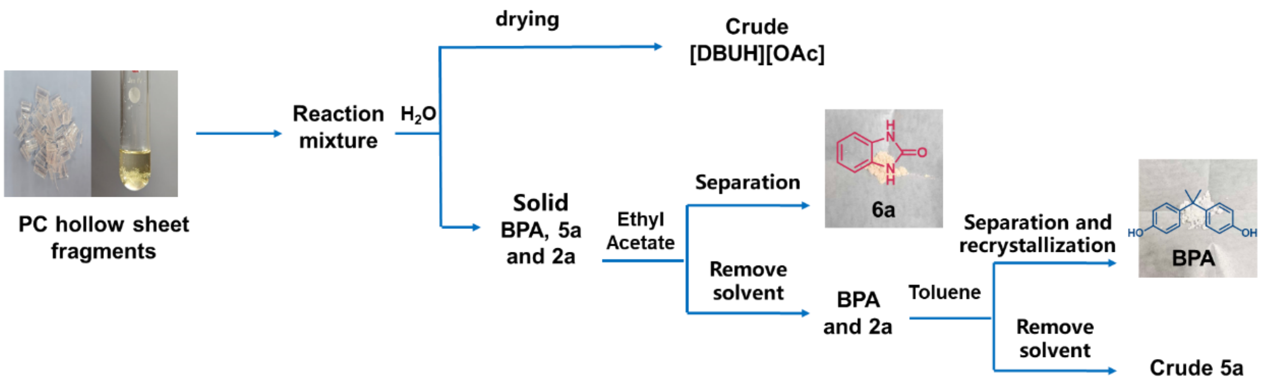

Figure S23. Simple separation procedure for BMO and BPA.

7.3 Techno-Economic Analysis

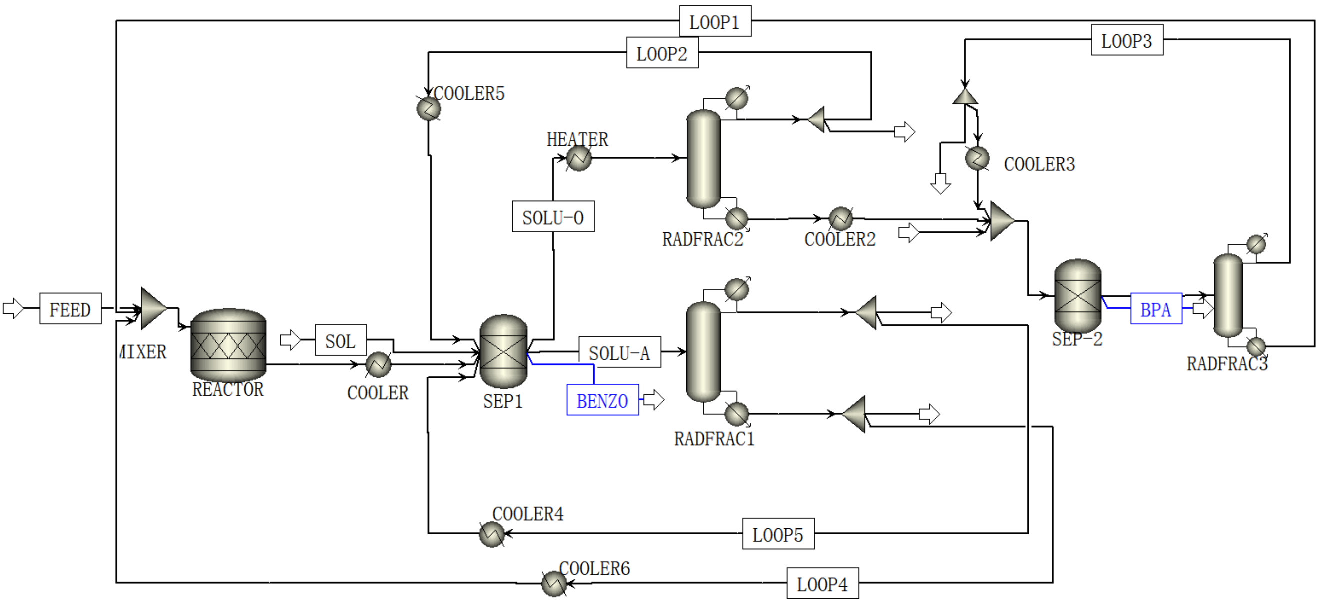

Figure S24. Process flow dia+gram for recycling waste PC to BPA and QDO, simulated using Aspen Plus.

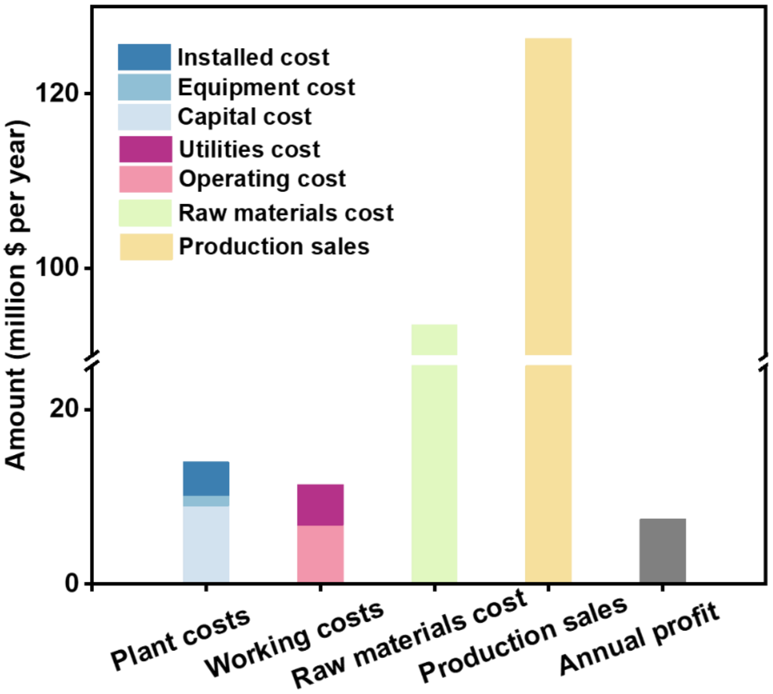

Figure S25. TEA analysis of recycling waste PC to BPA and QDO, including cost contribution, product sales, and annual profit.

Table S5. Techno-economic analysis of recycling waste PC to BPA and QDO via *in-situ* CO_2 capturing strategy.

Items		Unit Price ($ / ton)	Data Source	Quantity (million $ / year)
Plant costs	Capital cost	-	Aspen Plus V11	8.95
	Equipment cost	-	Aspen Plus V11	1.20
	Installed cost	-	Aspen Plus V11	3.82
	Total		-	13.97
Working costs	Operating cost	-	Aspen Plus V11	6.75
	Utilities cost	-	Aspen Plus V11	4.64
	Total		-	11.39
Total raw materials cost	Waste PC	714	https://www.recycle366.com	7.14
	ABN	18571	https://www.caigoubang.com.cn	86.38
	Total		-	93.52
Total production sales	BPA	1100	https://www.100ppi.com	9.89
	QDO	19000	https://www.caigoubang.com.cn	116.44
	Total		-	126.33
Annual profit				7.45

Table S6. Total equipment and installed cost of recycling waste PC to BPA and QDO via *in-situ* CO_2 capturing strategy.

Items	Equipment cost [$]	Installed cost [$]
HEATER	9100	70500
SEP1	19500	121900
COOLER6	11200	72800
COOLER5	10300	68500
REACTOR	68000	217700
RADFRAC3	147900	599200
RADFRAC1	487000	1213100
SEP2	16900	118100
COOLER4	9200	67100
COOLER2	10800	73000
RADFRAC2	391000	1057600
COOLER	11200	72300
COOLER3	10300	70900
Total	1202400	3822700

Mass and energy balances were established using Aspen Plus V11 based on an industrially relevant throughput of 10,000 $t \cdot yr^{-1}$ of post-consumer waste PC chips, and equipment sizing and cost estimation were conducted to quantify capital expenditure and operating expenditure. The process simulation and equipment sizing were built upon the separation procedure described in Section 7.1, and the corresponding Aspen flowsheet is provided in Figure S24. The complete economic inventory is summarized in Table S5, including equipment and installed costs (Table S6 for details) and an annual operating cost and profitability summary. The analysis indicates a total equipment cost of 1.20 million USD, a total installed cost of 3.82 million USD, and an estimated total capital investment of 13.97 million USD. The annual operating expenditure (excluding feedstock) is 11.39 million USD per year, of which utilities account for 4.64 million USD per year.

Moreover, product prices were collected from representative sales platforms at the time of analysis. We note that such market quotations can vary with supply–demand conditions and transaction volumes and may not strictly represent the realized prices at the exact production scale; nevertheless, they provide a reasonable reference for a screening-level assessment. Combining these price quotations with the simulated production rates, the TEA yields a total raw materials cost of 93.52 million USD per year, a total production sales revenue of 126.33 million USD per year, and an annual profit of 7.45 million USD per year (Figure S25). These results suggest that the proposed route shows promising profitability under the stated assumptions, supporting its techno-economic feasibility on a scale. Nevertheless, the present analysis does not constitute a fully rigorous economic evaluation, as reliable scale-consistent prices for certain products and some raw materials are difficult to determine accurately (public quotes are often sparse and strongly dependent on purity specifications, contract terms, and order size), and thus the absolute profitability metrics should be interpreted with caution and regarded as indicative rather than definitive.

7.4 Life cycle assessment

Table S7. Goal and scope of this LCA study

Goal	
Reason and scope	Comparing the different synthetic pathways for preparing QDO from recycled waste PC and CO_2.
Audience	Industrial stakeholders, the research community, and the public
Application	Support the quantification of carbon-emission reductions and the analysis of circular-economy policies relevant to chemical recycling of waste polycarbonate.
Intention to use results in comparative studies	Yes, the results are to be compared and disclosed to the public through this article's publication
Scope	
Product system	The upcycling of polycarbonate is based on GLO.
Functional unit	Production of 1 kg QDO
System boundary	Cradle-to-Gate
Allocation	All environmental effects are allocated to quinazoline-2,4(1H,3H)-dione (QDO).
Assumptions	(I) The pre-treatment discharge of waste PC is consistent with the mechanical method.<br>(II) This system produce 766 kg QDO/per hour over 8000 hours/per year.
Requirements on data and quality	Foreground material and energy consumption data were obtained from simulation in Aspen Plus V11 and the background processes were chosen based on OpenLCA 2.4.0.
LCIA methodology	IPCC 2013 GWP 100a for GWP;<br>CML-IA baseline for NREU;<br>Recipe 2016 Midpoint(H) for others.
Impact categories assessed	1. GWP, 100a, kg CO_2 equivalent;<br>2. NREU, MJ;<br>3. Qzone formation, kg NO_x eq<br>4. Terrestrial acidification, kg SO_2 eq<br>5. Human ecotoxicity, kg 1,4-DCB

	6. Land use, m^2a crop eq<br>7. Water consumption, m^3
limitations	In addition to the above-mentioned assumptions, the following aspects are not assessed: plant construction and equipment maintenance.
Report requirements	To present the outcome via journal publication which is openly accessible to everyone.

Note: A cradle-to-gate system boundary was applied in accordance with ISO 14040 and implemented using a cut-off approach. It includes: (i) raw-material acquisition, covering the collection, transport, and pretreatment of post-consumer PC as well as other process inputs; and (ii) the production and purification of and QDO, encompassing PC depolymerization and downstream separation/purification. The functional unit was defined as production of 1 kg QDO. Mass and energy balances were established via Aspen Plus V11 simulations based on an annual throughput of 10,000 t of waste PC chips.

Table S8. LCA results the different synthetic pathways for preparing QDO from recycled waste PC (this work) and CO_2.

Environmental impact category	Unit	CO_2 synthesis pathway	This work
GWP	kg CO_2 eq	4.39	2.21
NREU	MJ per kg	80.80	-1.66
Qzone formation	kg NO_x eq	0.0078	0.0002
Terrestrial acidification	kg SO_2 eq	0.0116	0.0048
Human ecotoxicity	kg 1,4-DCB	2.47	0.98
Land use	m^2a crop eq	0.039	0.003
Water consumption	m^3	0.091	0.021

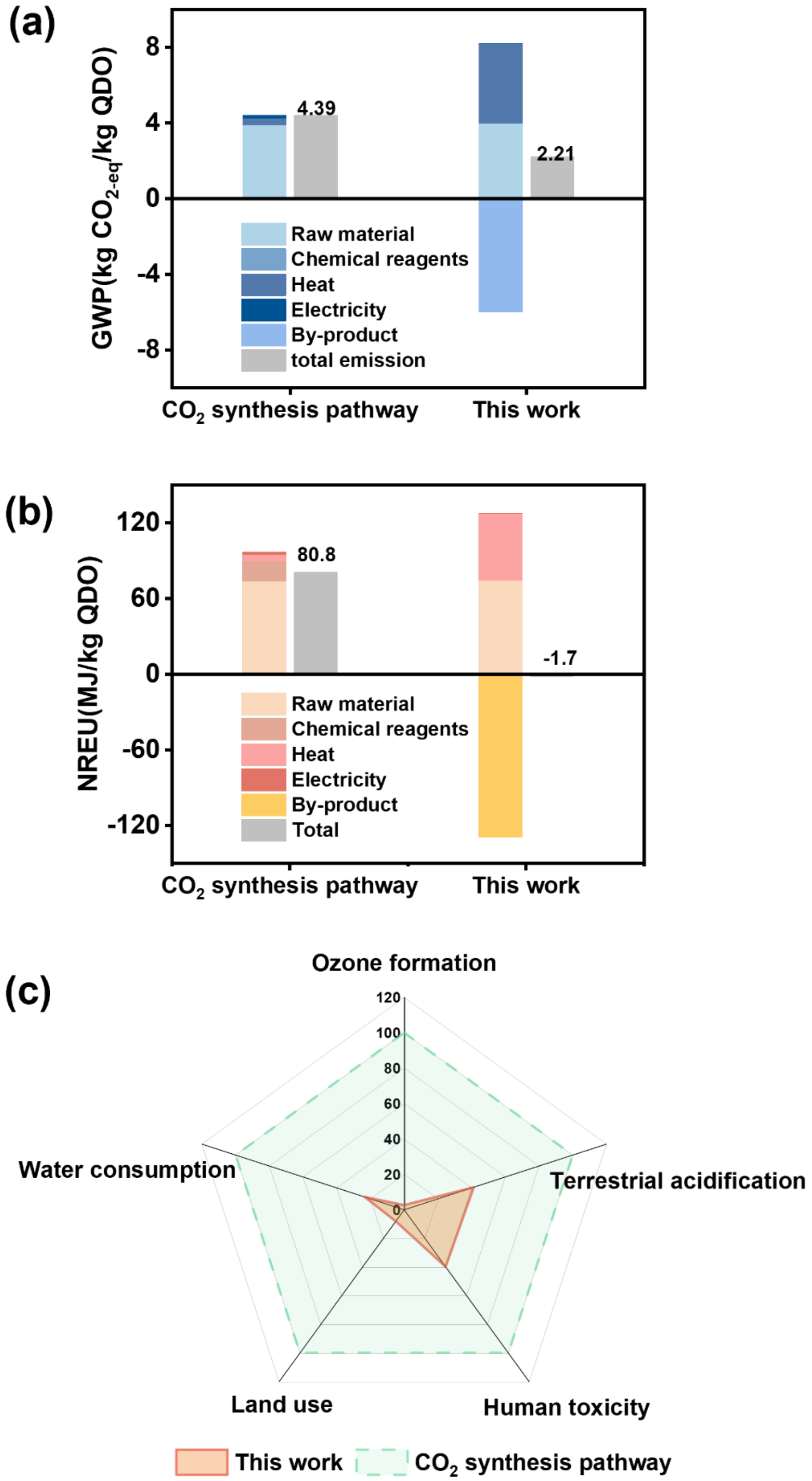

Figure S26. LCA comparison of the different synthetic pathways for preparing QDO from recycled waste PC (this work) and CO_2. (a-b) GWP and NREU. (c) Other categories of environmental impacts.

We conducted a cradle-to-gate life cycle assessment (LCA) in accordance with ISO 14040 and implemented a cut-off approach to compare two synthetic pathways for producing quinazoline-2,4(1H,3H)-dione (QDO): (i) a CO_2 synthesis pathway (CO_2 + o-aminobenzonitrile) and (ii) this work,

where QDO is produced from recycled post-consumer waste polycarbonate (PC) via *in-situ* CO_2 capture and upcycling (Table S7). The functional unit is defined as the production of 1 kg QDO, and the system boundary is cradle-to-gate, including: (i) raw-material acquisition (collection, transport, and pretreatment of post-consumer PC and other process inputs) and (ii) QDO production and purification (reaction, separation, and downstream processing). The product system is modeled using a global (GLO) background. Foreground mass and energy inventories were established from Aspen Plus V11 simulations, while background datasets were sourced from openLCA 2.4.0. The industrially relevant scale is modeled as 10,000 t/year waste PC throughput, corresponding to a plant output of 766 kg QDO per hour over 8000 hours per year. Allocation follows the defined goal of comparing QDO pathways: all environmental effects are allocated to QDO (Table S7).

The life cycle impact assessment applies IPCC 2013 GWP 100a for GWP (kg CO_2-eq), CML-IA baseline for NREU (MJ per kg QDO), and ReCiPe 2016 Midpoint (H) for additional midpoint indicators: ozone formation (kg NO_x-eq), terrestrial acidification (kg SO_2-eq), human ecotoxicity (kg 1,4-DCB-eq), land use (m^2a crop-eq), and water consumption (m^3) (Table S7). The comparative cradle-to-gate results are summarized in Figure S26 (a-c) and Table S8. Per 1 kg QDO, the CO_2 synthesis pathway exhibits higher burdens than this work across all reported categories: GWP decreases from 4.39 to 2.21 kg CO_2-eq, and NREU decreases from 80.80 to -1.66 MJ. Likewise, ozone formation decreases from 0.0078 to 0.0002 kg NO_x-eq, terrestrial acidification from 0.0116 to 0.0048 kg SO_2-eq, human ecotoxicity from 2.47 to 0.98 kg 1,4-DCB-eq, land use from 0.039 to 0.003 m^2a crop-eq, and water consumption from 0.091 to 0.021 m^3. As illustrated by the contribution analysis in Figure S26 (a) and (b), the improved performance of this work is strongly influenced by the by-product credits, which offset part of the upstream energy and emission burdens under the adopted cut-off modeling framework, yielding a slightly negative NREU. Overall, these results demonstrate that producing QDO via recycled-PC upcycling with *in-situ* CO_2 capture can deliver substantially lower cradle-to-gate environmental impacts than direct CO_2-based QDO synthesis within the defined scope and assumptions.

8. ^{1}H NMR and ^{13}C NMR data of all compounds

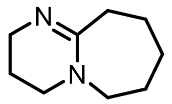

DBU **3b**: ^{1}H NMR (600 MHz, DMSO-*d*6) δ = 2.81-2.74 (m, 5H), 1.89-1.91 (m, 2H), 1.28-1.33 (m, 2H), 1.13-1.24 (s, 6H); ^{13}C NMR (151 MHz, DMSO-*d*6) δ = 158.4, 51.6, 43.6, 36.2, 29.0, 28.1, 25.6, 22.3.

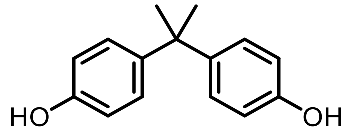

BPA **3b**: ^{1}H NMR yield 99%; ^{1}H NMR (600 MHz, DMSO-*d*6) δ = 9.13 (s, 2H), 6.98 (d, *J* = 10.8 Hz, 4H), 6.64 (d, *J* = 10.2 Hz, 4H), 1.52 (s, 6H); ^{13}C NMR (151 MHz, DMSO-*d*6) δ = 154.9, 141.1, 127.3, 114.6, 40.9, 30.9.

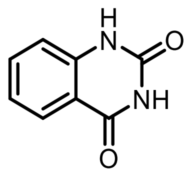

Quinazoline-2,4(1*H*,3*H*)-dione **4a**: ^{1}H NMR yield 96%; ^{1}H NMR (600 MHz, DMSO-*d*6) δ = 11.27 (s, 1H), 11.13 (s, 1H), 3.91 (s, 1H), 8.22 (d, *J* = 8.4 Hz, 2H), 7.62 (t, *J* = 10.2 Hz, 2H), 7.17 (t, *J* = 9.0 Hz, 3H); ^{13}C NMR (151 MHz, DMSO-*d*6) δ = 162.8, 150.3,140.9, 135.0, 127.0, 122.3, 115.3, 114.3.

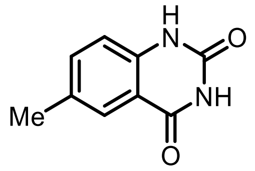

6-Methylquinazoline-2,4(1*H*,3*H*)-dione **4b**: ^{1}H NMR yield 90%; ^{1}H NMR (600 MHz, DMSO-*d*6) δ = 11.19 (s, 1H), 11.03 (s, 1H), 7.88 (d, *J* = 7.2 Hz, 1H), 7.42 (d, *J* = 7.2 Hz, 1H), 7.05 (d, *J* = 10.2 Hz, 1H), 2.30 (s, 3H); ^{13}C NMR (151 MHz, DMSO-*d*6) δ = 163.3, 150.8, 139.2, 136.4, 132.0, 126.9, 115.8, 114.6, 20.7.

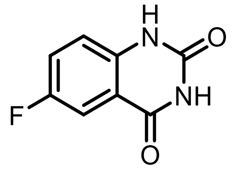

6-Fluoroquinazoline-2,4(1*H*,3*H*)-dione **4c**: ^{1}H NMR yield 92%; ^{1}H NMR (600 MHz, DMSO-*d*6) δ = 11.29 (s, 2H), 7.57-7.50 (m, 2H), 7.19-7.16 (m, 1H); ^{13}C NMR (151 MHz, DMSO-*d*6) δ = 162.1, 157.3, 150.1, 137.6, 122.9, 117.6, 115.4, 112.0.

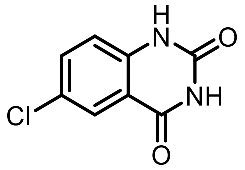

6-Chloroquinazoline-2,4(1*H*,3*H*)-dione **4d**: ^{1}H NMR yield 88%; ^{1}H NMR (600 MHz, DMSO-*d*6) δ = 11.33 (s, 2H), 7.78 (d, *J* = 3.0 Hz, 1H), 7.64 (dd, *J* = 3.0, 10.8 Hz, 1H), 7.78 (d, *J* = 10.8 Hz, 1H); ^{13}C NMR (151 MHz, DMSO-*d*6) δ = 162.3, 150.6, 140.3, 135.3, 126.8, 126.4, 118.0, 116.3.

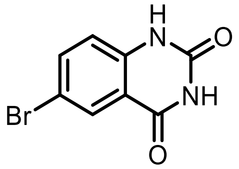

6-Bromoquinazoline-2,4(1*H*,3*H*)-dione **4e**: ^{1}H NMR yield 93%; ^{1}H NMR (600 MHz, DMSO-*d*6) δ = 11.34 (s, 2H), 7.92 (d, *J* = 3.0 Hz, 1H), 7.77 (dd, *J* = 2.4, 10.2 Hz, 1H), 7.11 (d, *J* = 10.8 Hz, 1H); ^{13}C NMR (151 MHz, DMSO-*d*6) δ = 161.7, 150.1, 140.1, 137.5, 128.9, 117.8, 116.2, 113.8.

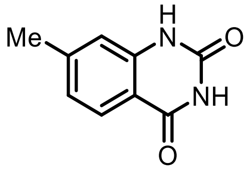

7-Methylquinazoline-2,4(1*H*,3*H*)-dione **4f**: ^{1}H NMR yield 83%; ^{1}H NMR (600 MHz, DMSO-*d*6) δ = 11.18 (s, 1H), 11.06 (s, 1H), 7.76 (d, *J* = 9.6 Hz, 1H), 7.77 (d, 11.4 Hz, 1H), 6.93 (s, 1H), 2.34 (s, 3H); ^{13}C NMR (151 MHz, DMSO-*d*6) δ = 162.7, 150.5, 145.6, 140.9, 126.9, 123.7, 115.1, 112.1, 21.4.

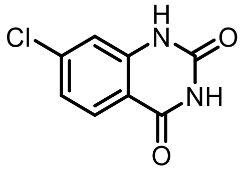

7-Chloroquinazoline-2,4(1*H*,3*H*)-dione **4g**: ^{1}H NMR yield 88%; ^{1}H NMR (600 MHz, DMSO-*d*6) δ = 11.31 (s, 2H), 7.86 (d, *J* = 10.2 Hz, 1H), 7.18 (dd, *J* = 2.4, 10.2 Hz, 1H), 7.11 (d, *J* = 2.4 Hz, 1H); ^{13}C NMR (151 MHz, DMSO-*d*6) δ = 162.1, 150.2, 142.0, 139.3, 129.0, 122.5, 114.7, 113.3.

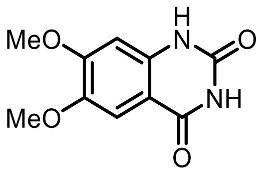

6,7-dimethoxyquinazoline-2,4(1*H*,3*H*)-dione **4h**: ^{1}H NMR yield 87%; ^{1}H NMR (600 MHz, DMSO-*d*6) δ = 11.61 (d, *J* = 6.9 Hz, 2H), 7.86 (s, 1H), 7.28 (s, 1H), 4.42 (s, 3H), 4.38 (s, 3H); ^{13}C NMR (151 MHz, DMSO-*d*6) δ = 162.5, 154.9, 150.4, 145.1, 136.6, 107.2, 106.2, 97.8, 55.8, 55.7.

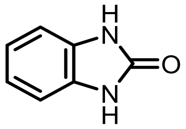

1,3-dihydro-2*H*-benzo[*d*]imidazol-2-one **6a**: ^{1}H NMR yield 97%; ^{1}H NMR (600 MHz, DMSO-*d*6) δ = 8.22 (d, *J* = 8.4 Hz, 2H), 7.55 (t, *J* = 9.0 Hz, 2H), 7.43 (t, *J* = 9.6 Hz, 3H), 3.91 (s, 3H); ^{13}C NMR (151 MHz, DMSO-*d*6) δ = 167.1, 132.9, 130.1, 129.5, 128.3, 52.0.

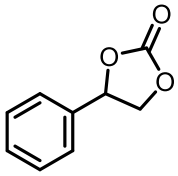

4-phenyl-1,3-dioxolan-2-one **6b**: ^{1}H NMR yield 97%; ^{1}H NMR (600 MHz, $CDCl_3$) δ = 7.39-7.41 (m, 3H), 7.35-7.33 (m, 2H), 5.65 (t, *J* = 8.4 Hz, 1H), 4.77 (t, *J* = 8.4 Hz, 1H), 4.30 (t, *J* = 7.8 Hz, 1H); ^{13}C NMR (151 MHz, DMSO-*d*6) δ = 154.8, 135.6, 129.4, 129.0, 125.7, 77.8, 71.0.

9. ^{1}H NMR and ^{13}C NMR spectra of all compounds

^{1}H NMR of DBU (600 MHz, DMSO-*d*6) and ^{13}C NMR of DBU (151 MHz, DMSO-*d*6)

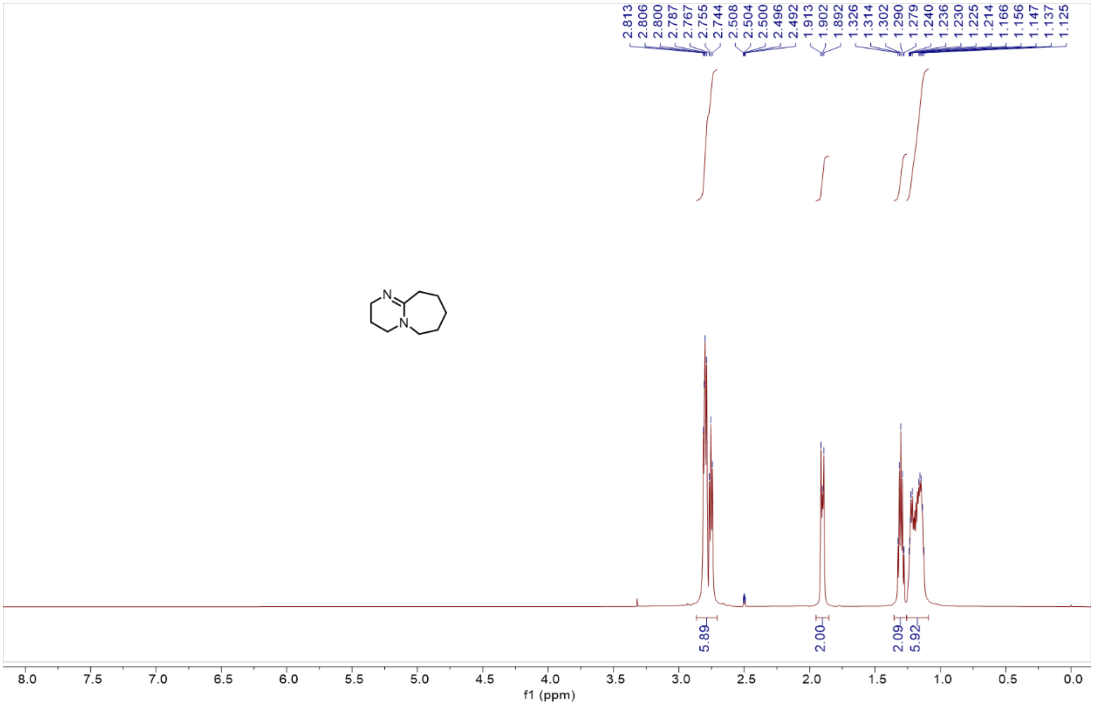

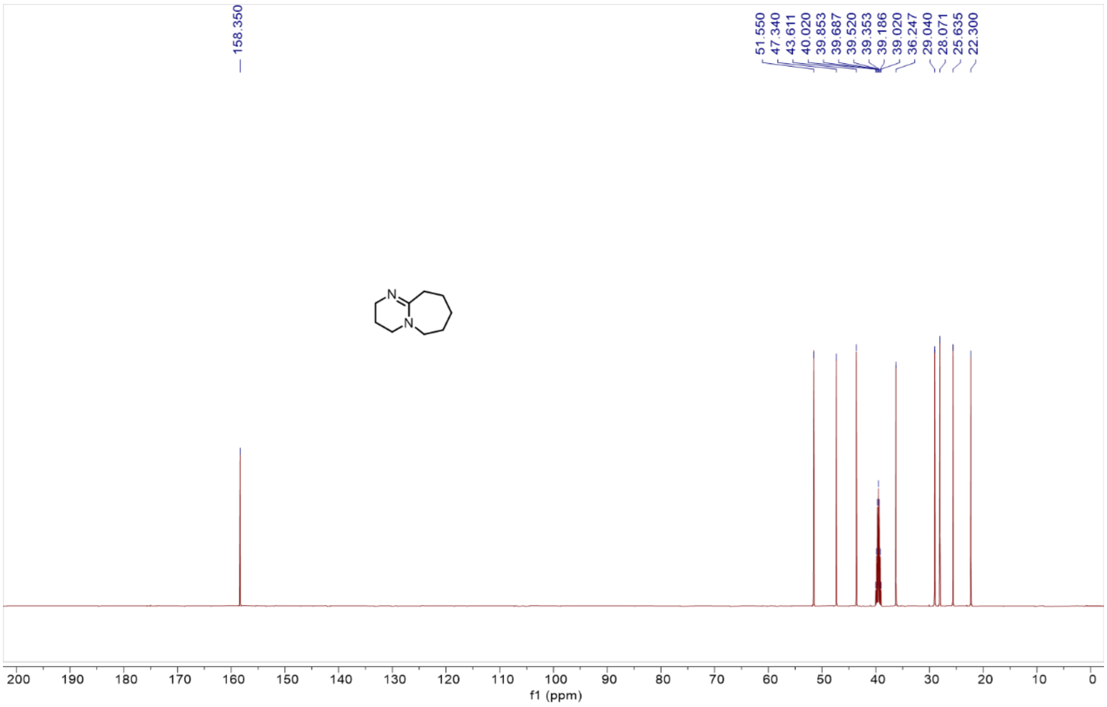

^{1}H NMR of [DBUH][OAc] (600 MHz, DMSO-*d*6) and ^{13}C NMR of [DBUH][OAc] (151 MHz, DMSO-*d*6)

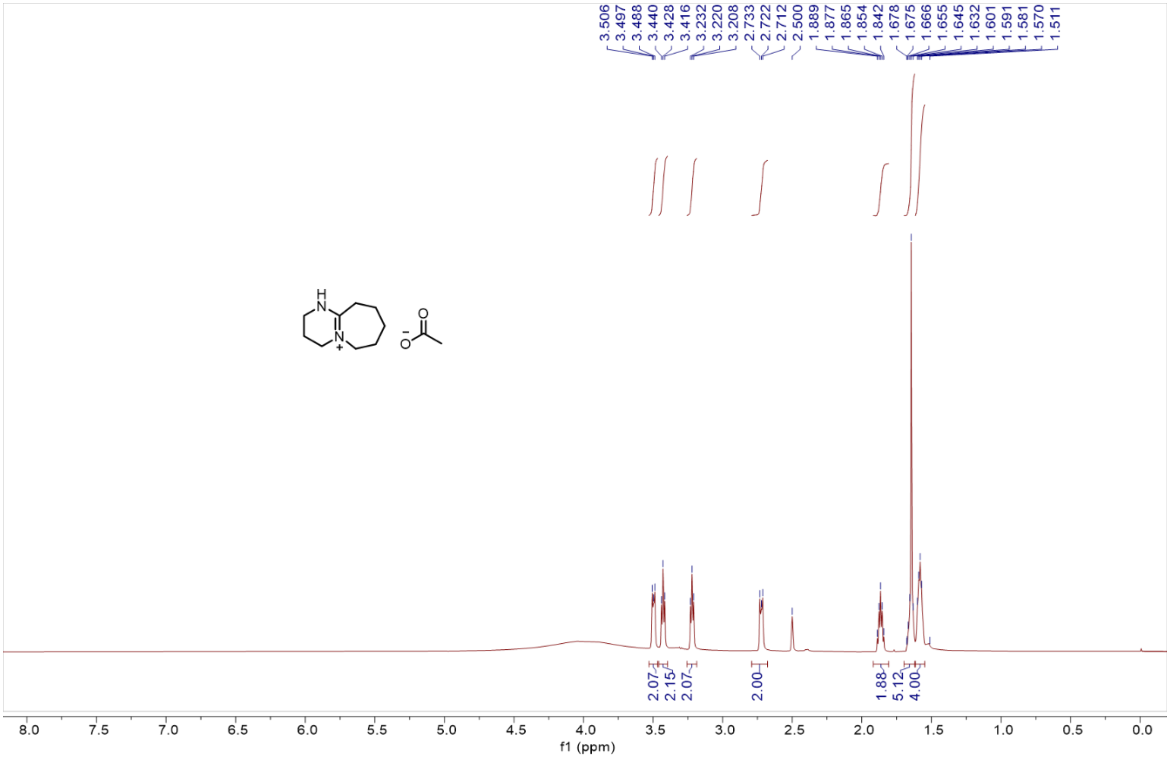

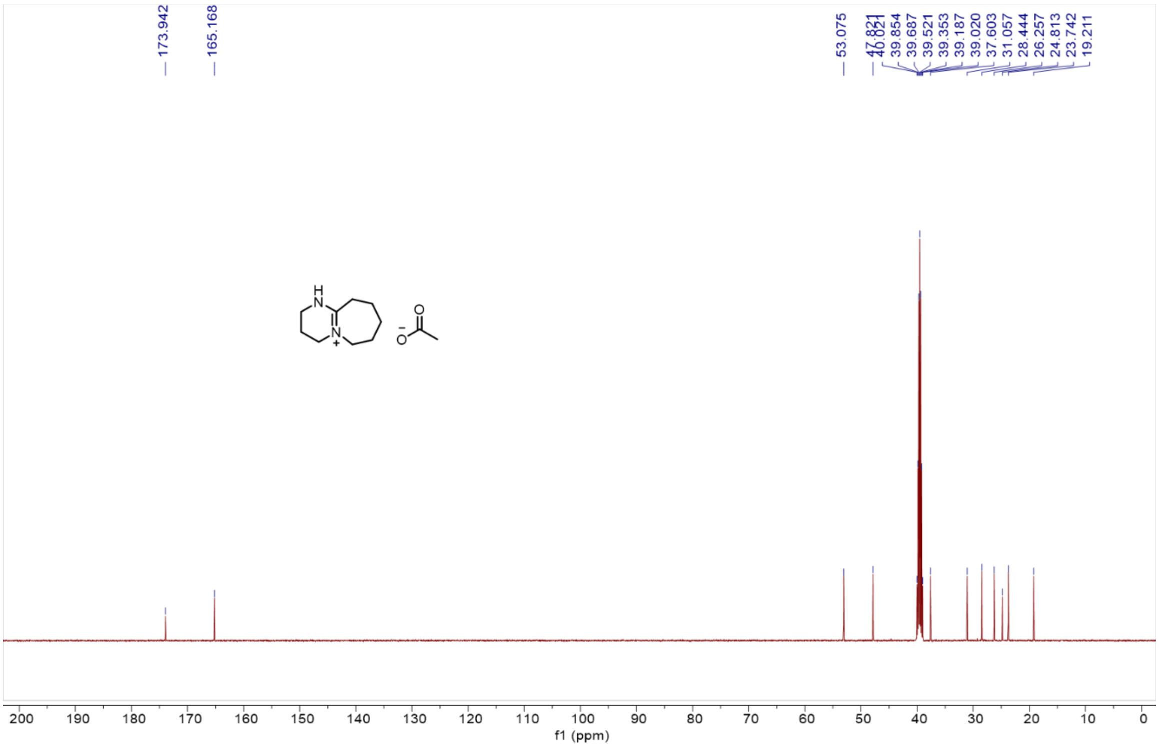

^{1}H NMR of BPA (600 MHz, DMSO-*d*6) and ^{13}C NMR of BPA (151 MHz, DMSO-*d*6)

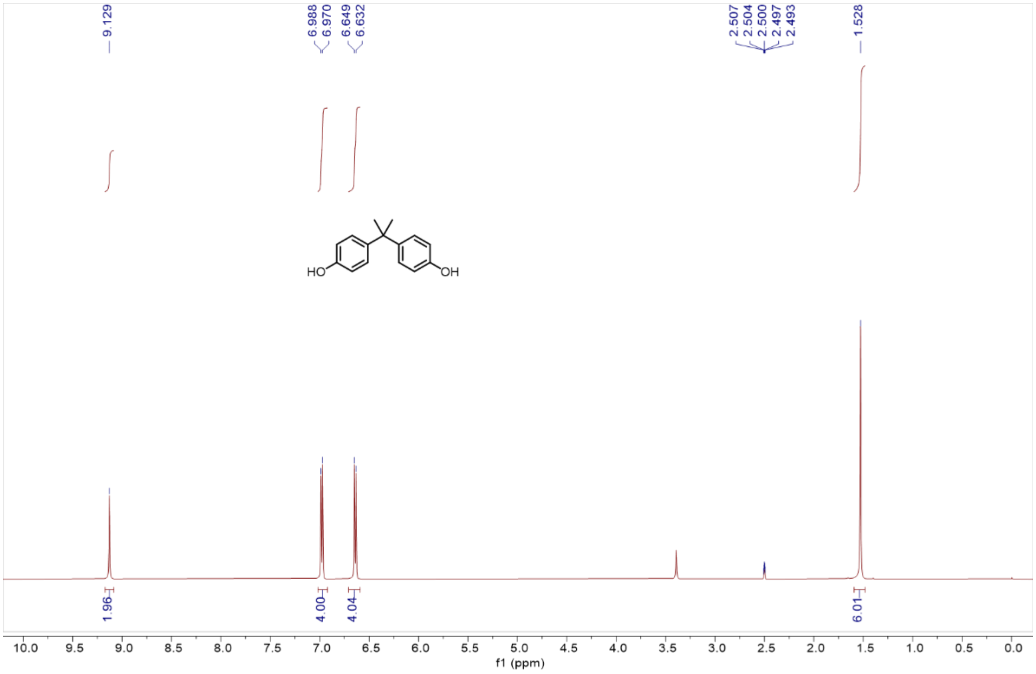

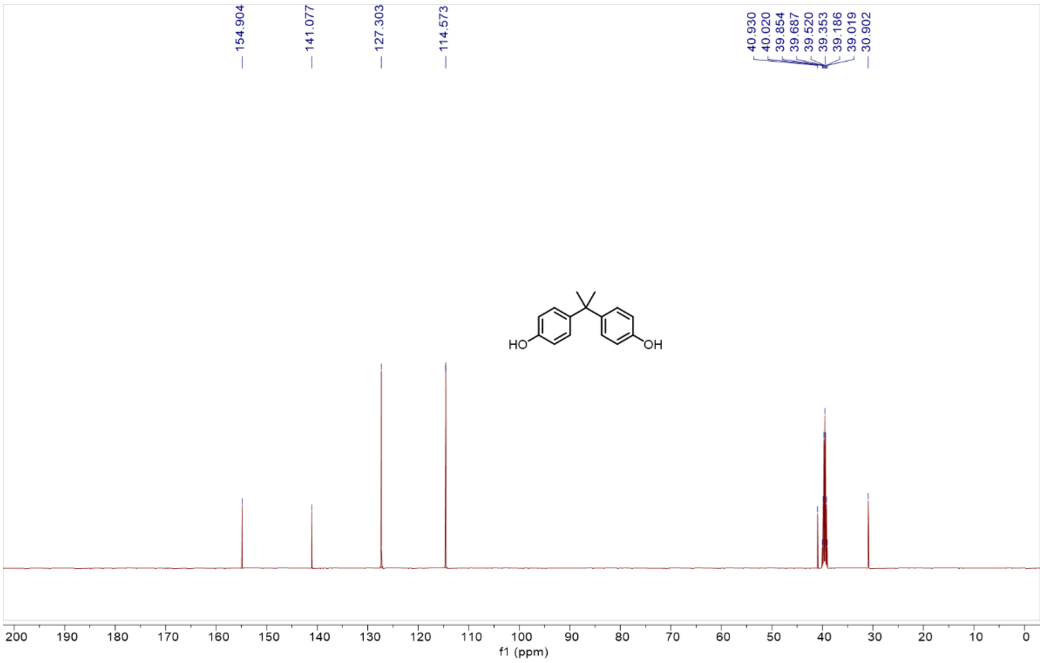

^{1}H NMR of **4a** (600 MHz, DMSO-*d*6) and ^{13}C NMR of **4a** (151 MHz, DMSO-*d*6)

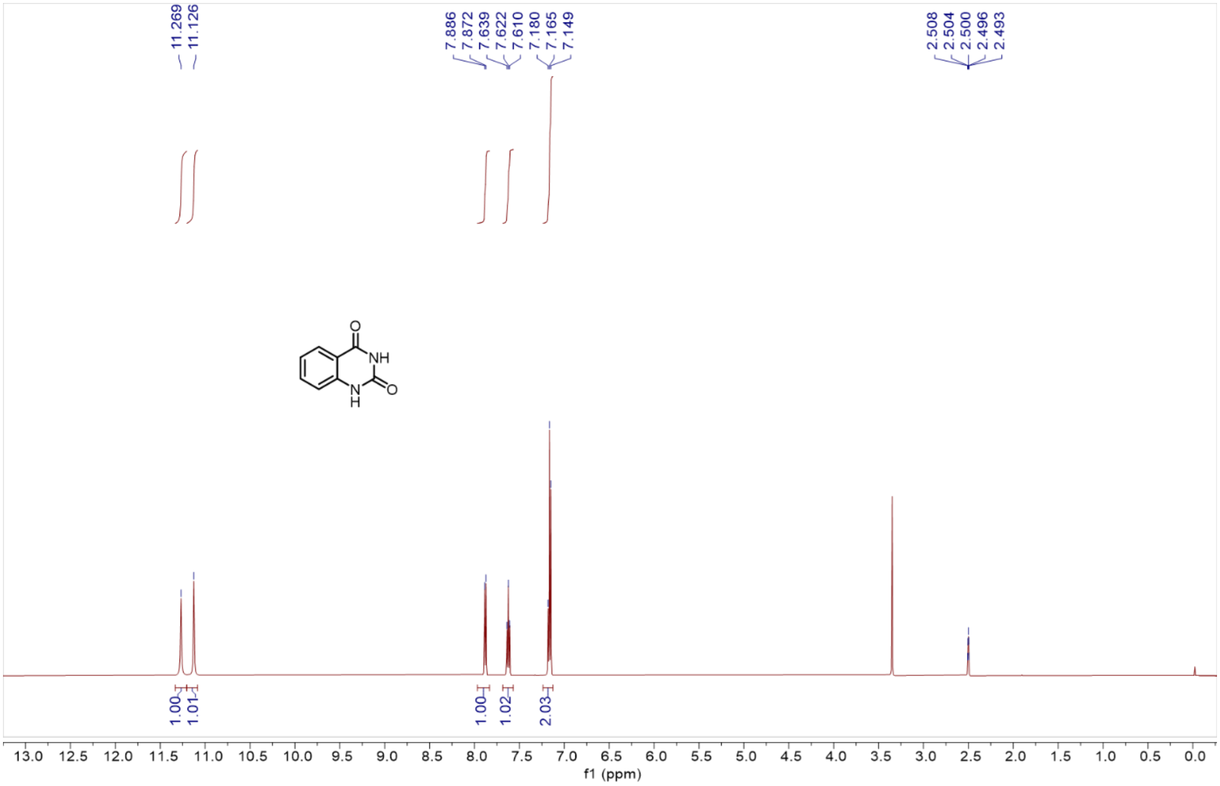

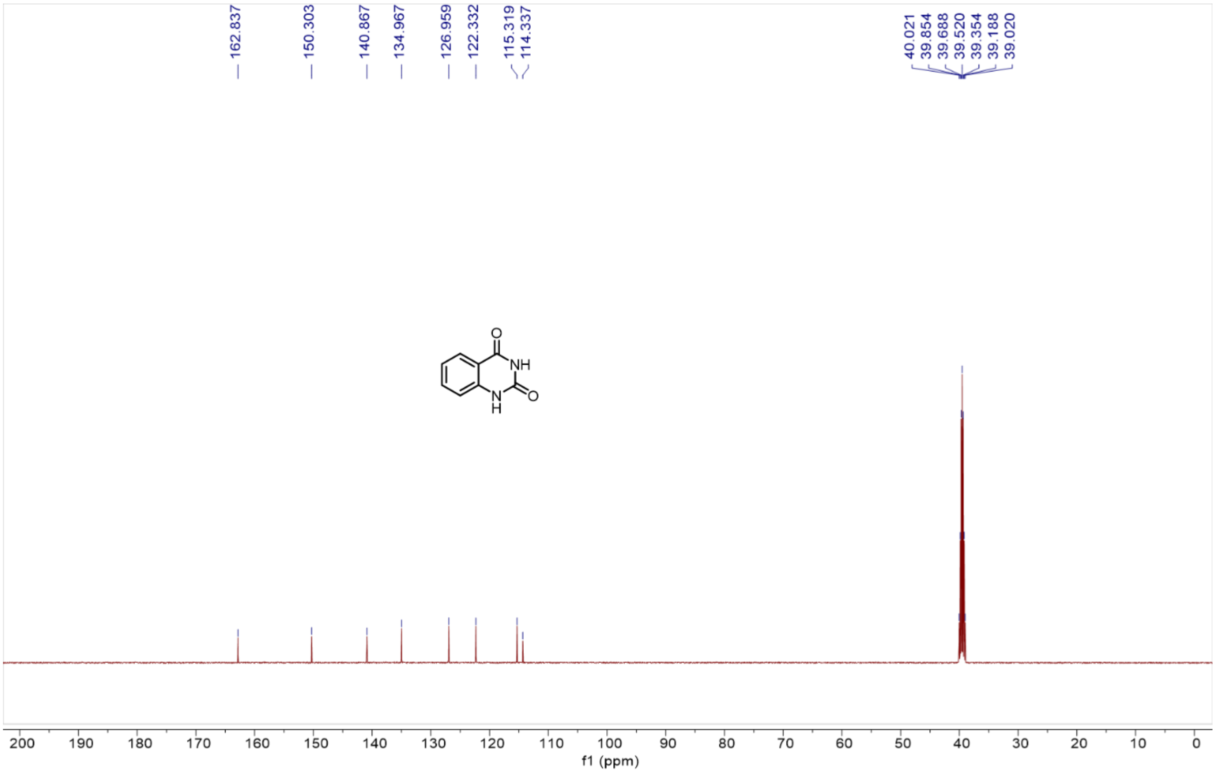

^{1}H NMR of **4b** (600 MHz, DMSO-*d*6) and ^{13}C NMR of **4b** (151 MHz, DMSO-*d*6)

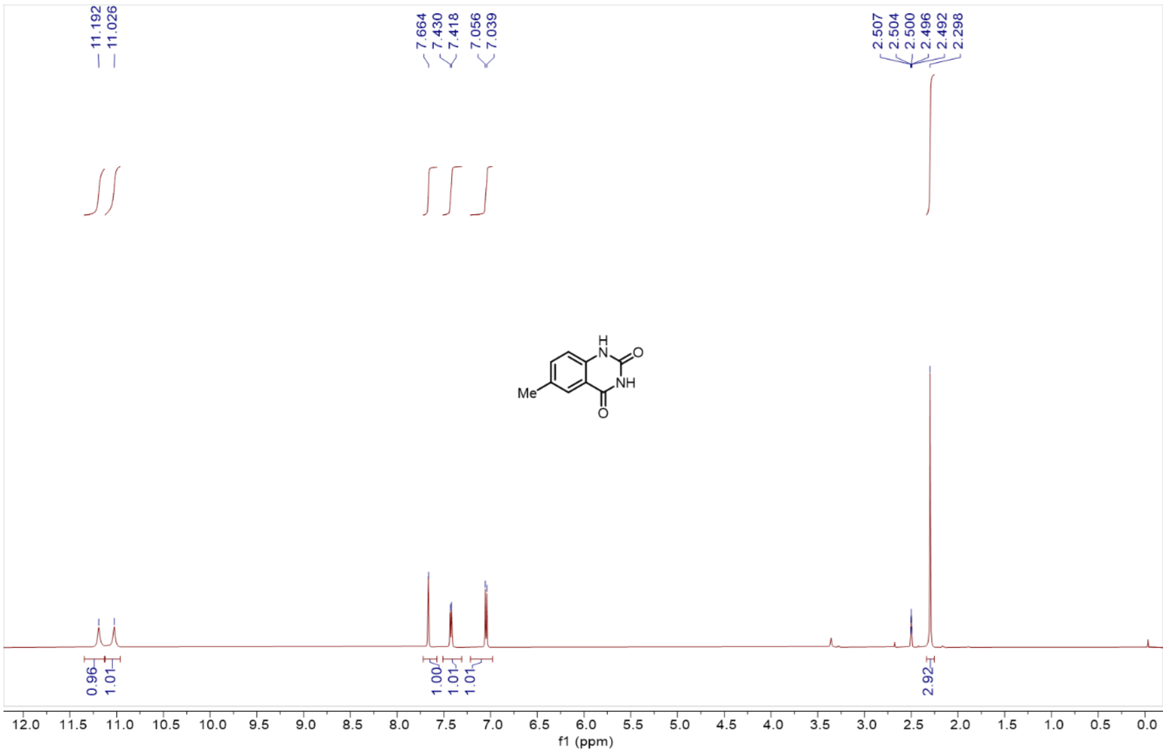

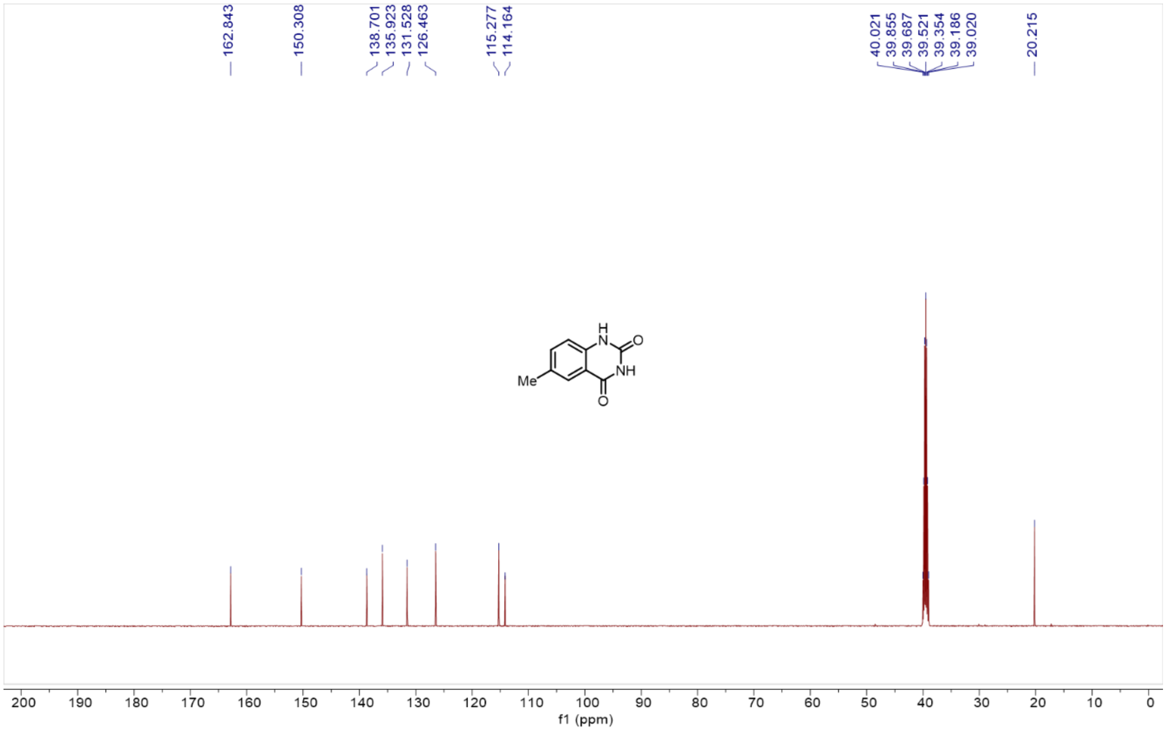

^{1}H NMR of **4c** (600 MHz, DMSO-*d*6) and ^{13}C NMR of **4c** (151 MHz, DMSO-*d*6)

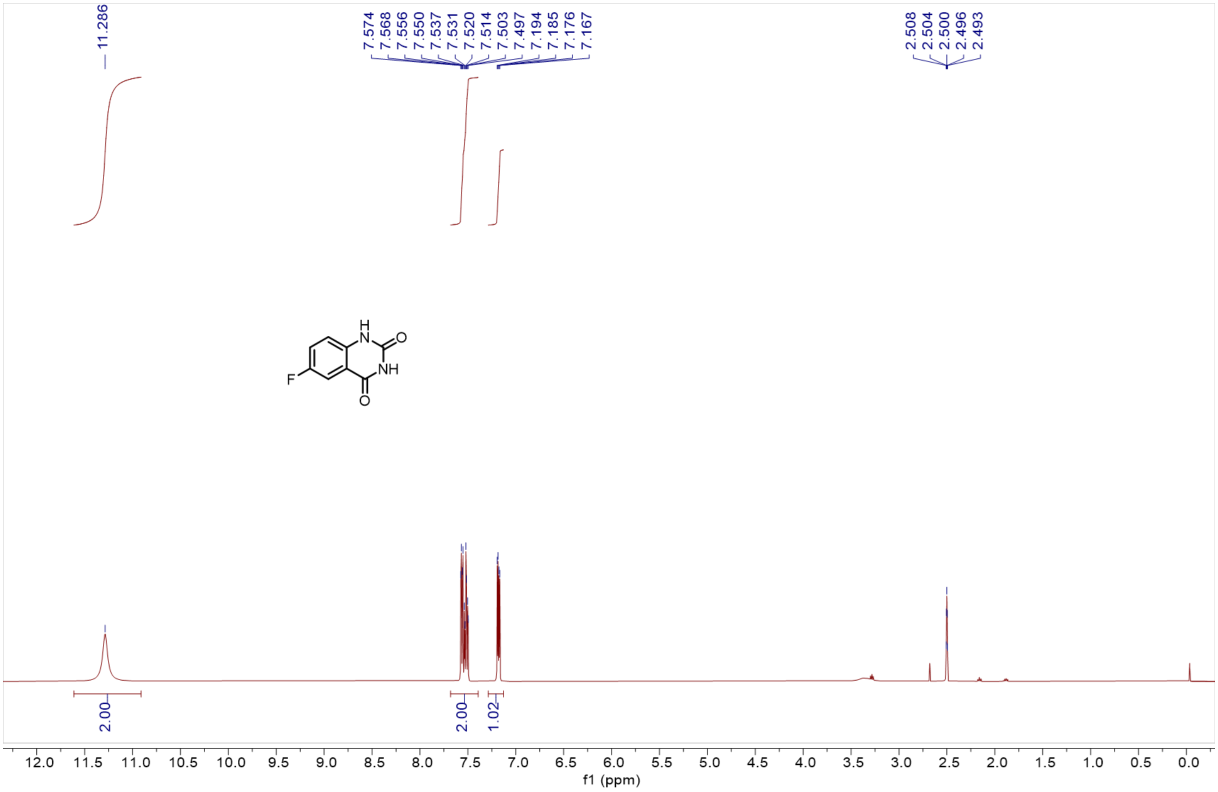

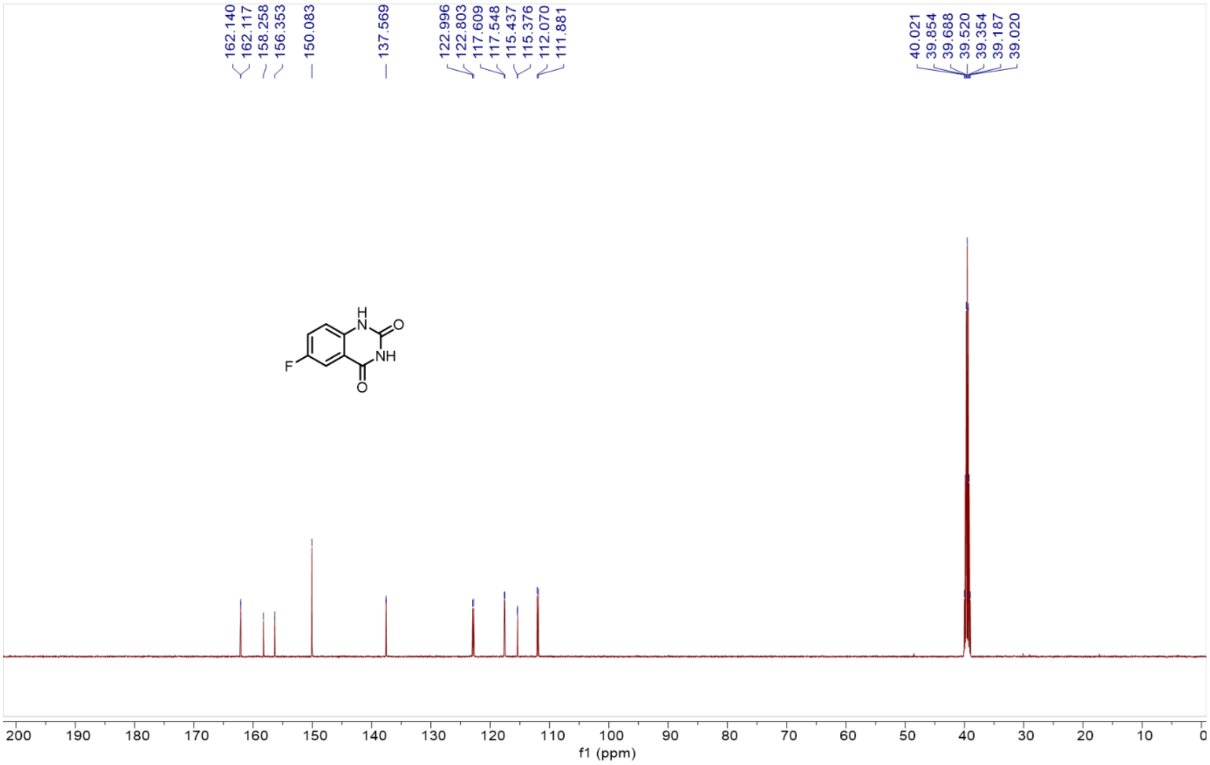

^{1}H NMR of **4d** (600 MHz, DMSO-*d*6) and ^{13}C NMR of **4d** (151 MHz, DMSO-*d*6)

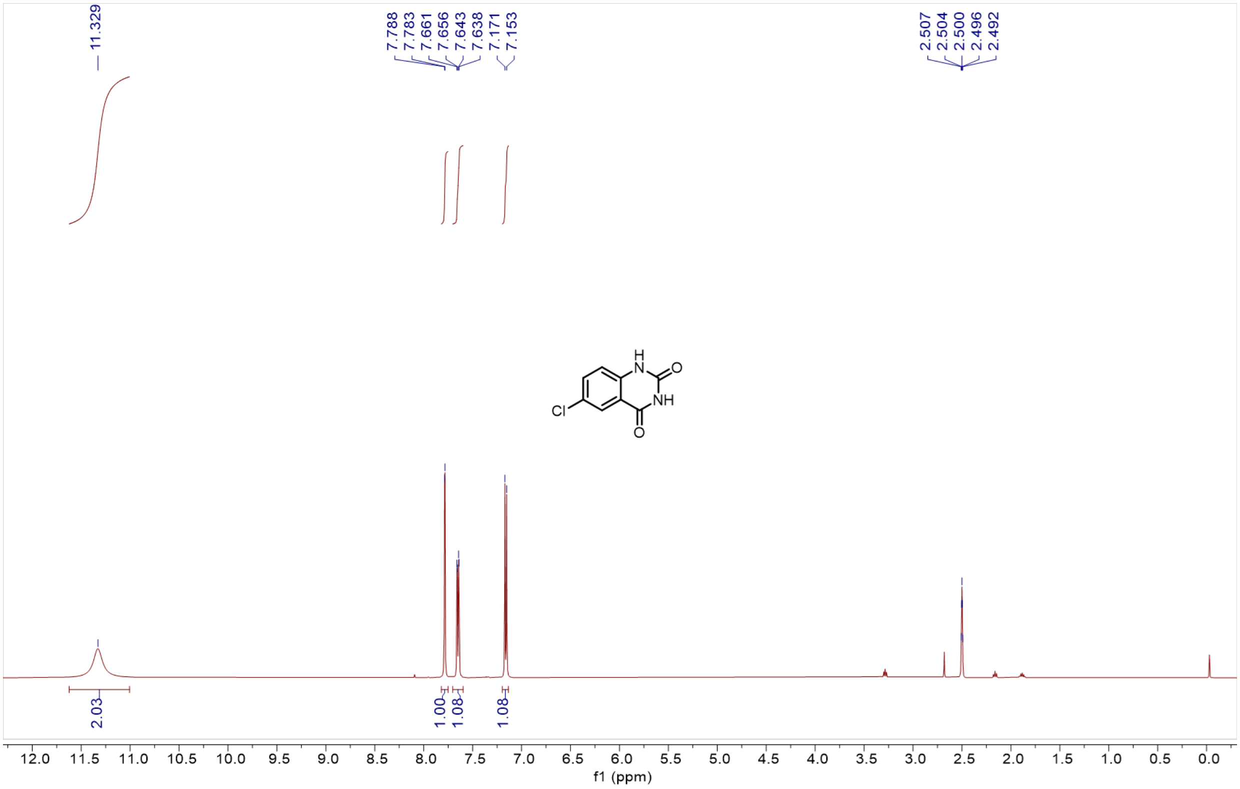

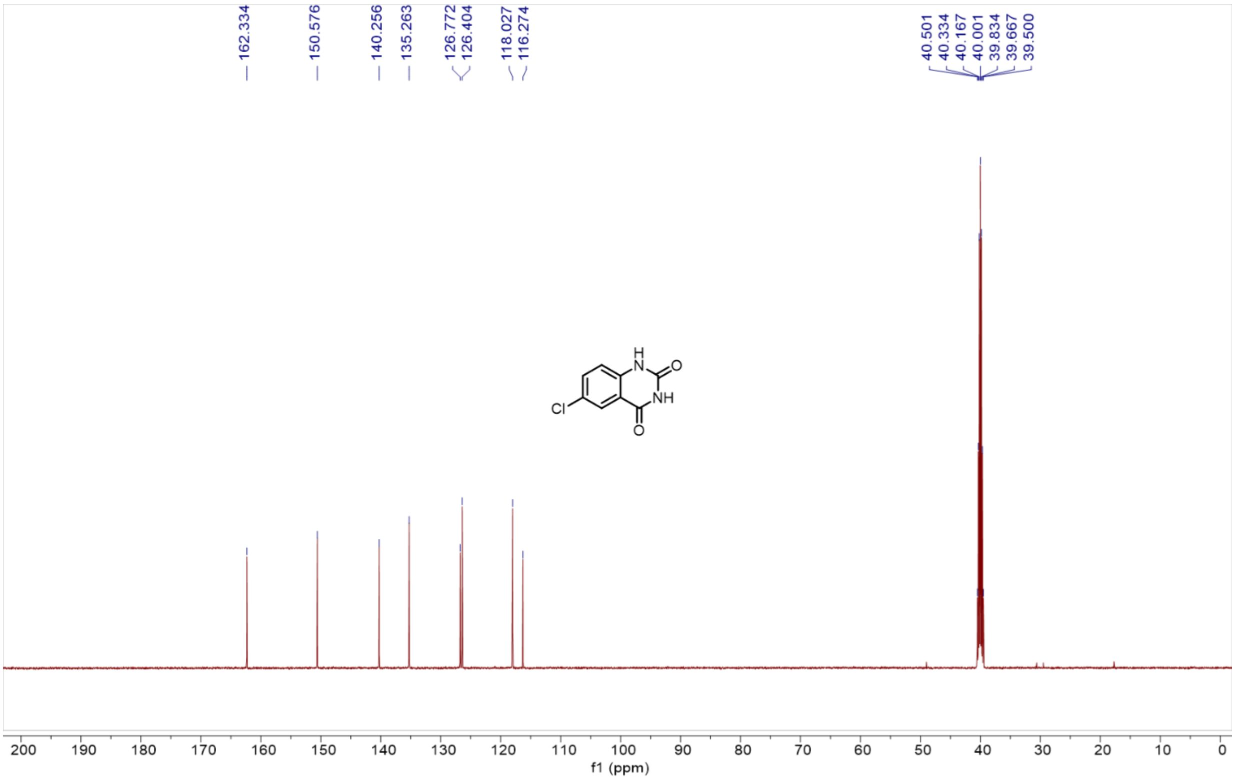

^{1}H NMR of **4e** (600 MHz, DMSO-*d*6) and ^{13}C NMR of **4e** (151 MHz, DMSO-*d*6)

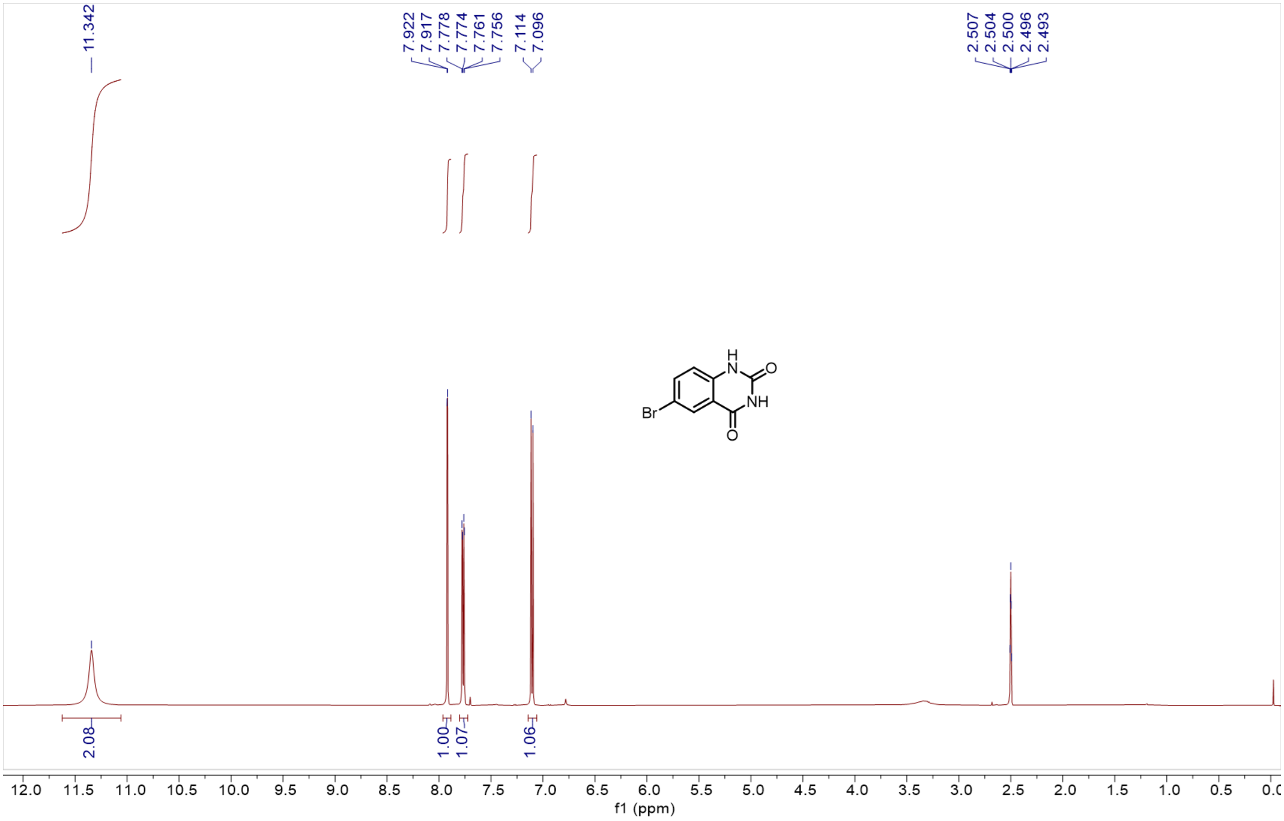

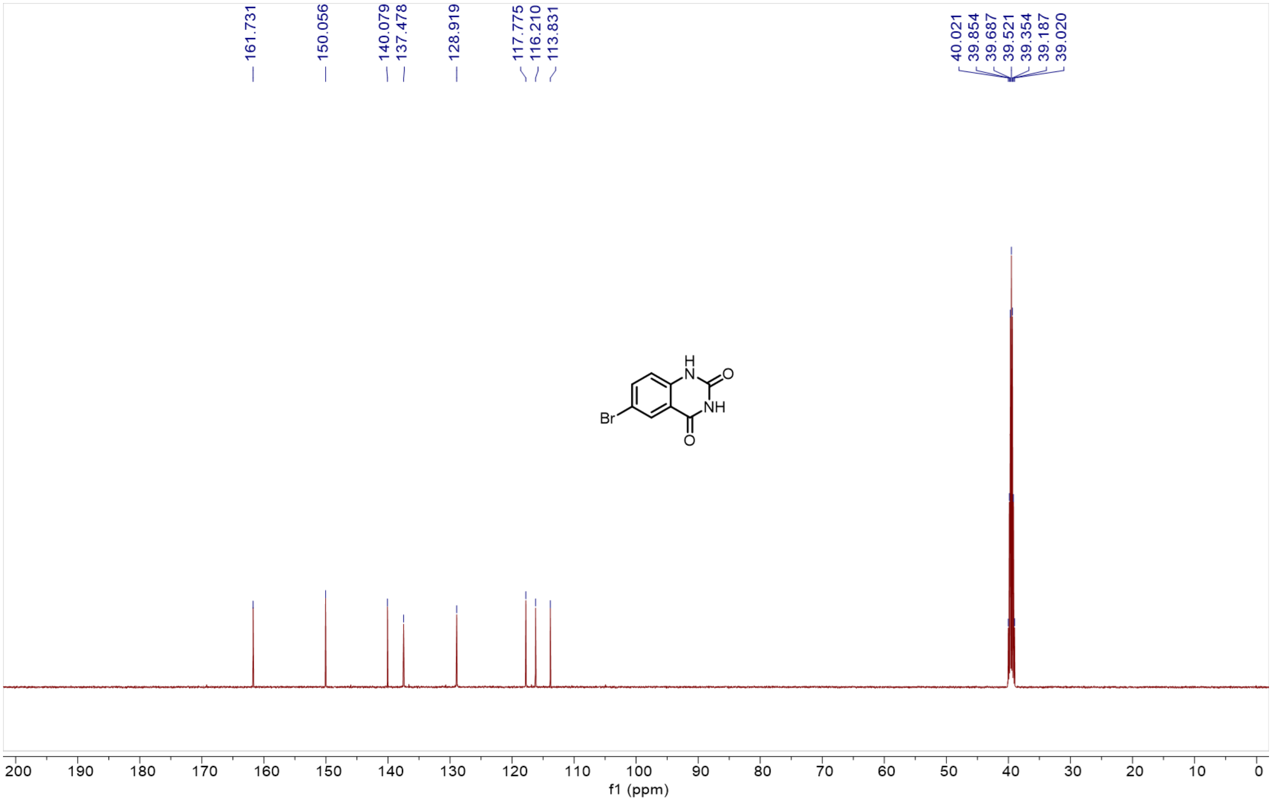

^{1}H NMR of **4f** (600 MHz, DMSO-*d*6) and ^{13}C NMR of **4f** (151 MHz, DMSO-*d*6)

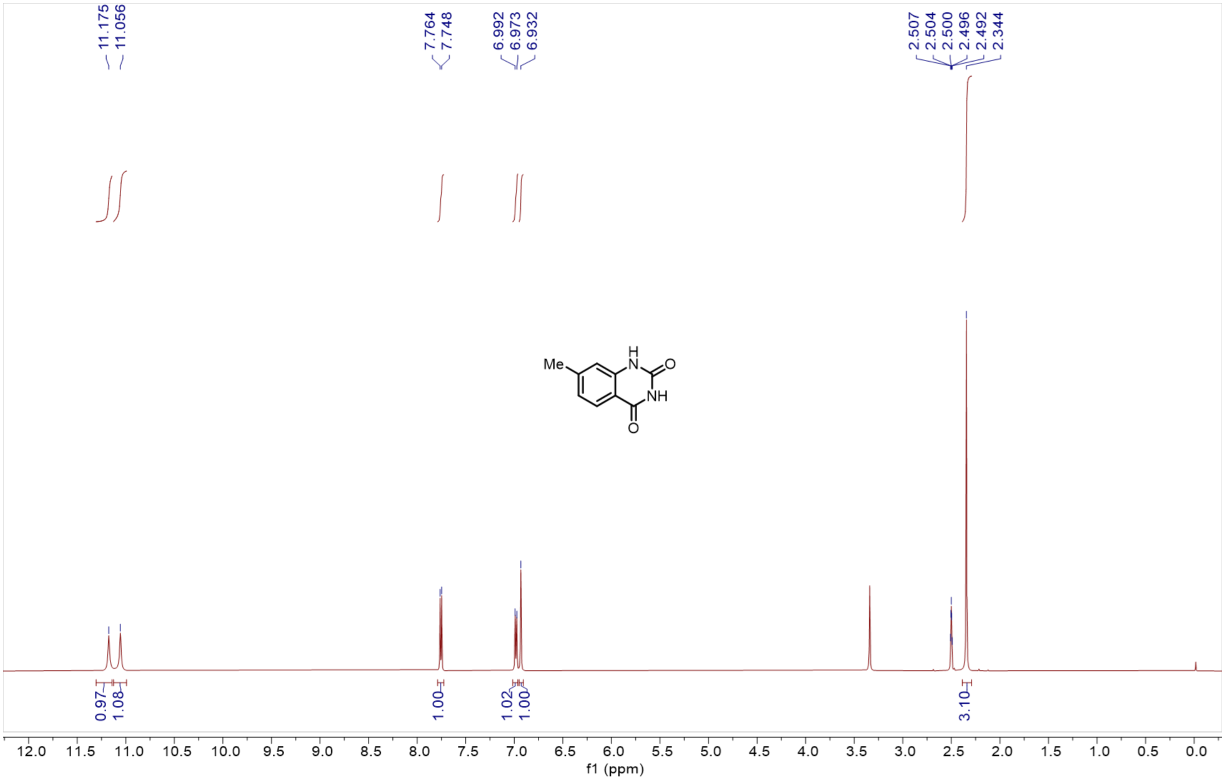

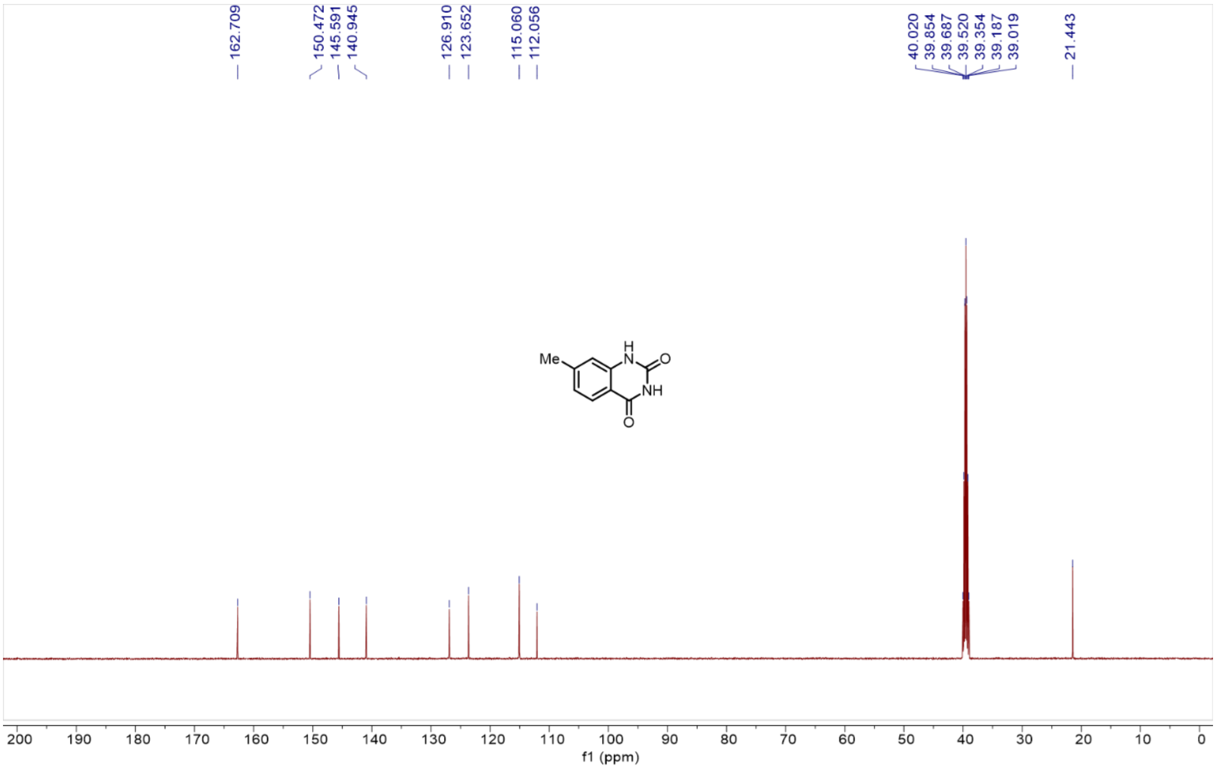

^{1}H NMR of **4g** (600 MHz, DMSO-*d*6) and ^{13}C NMR of **4g** (151 MHz, DMSO-*d*6)

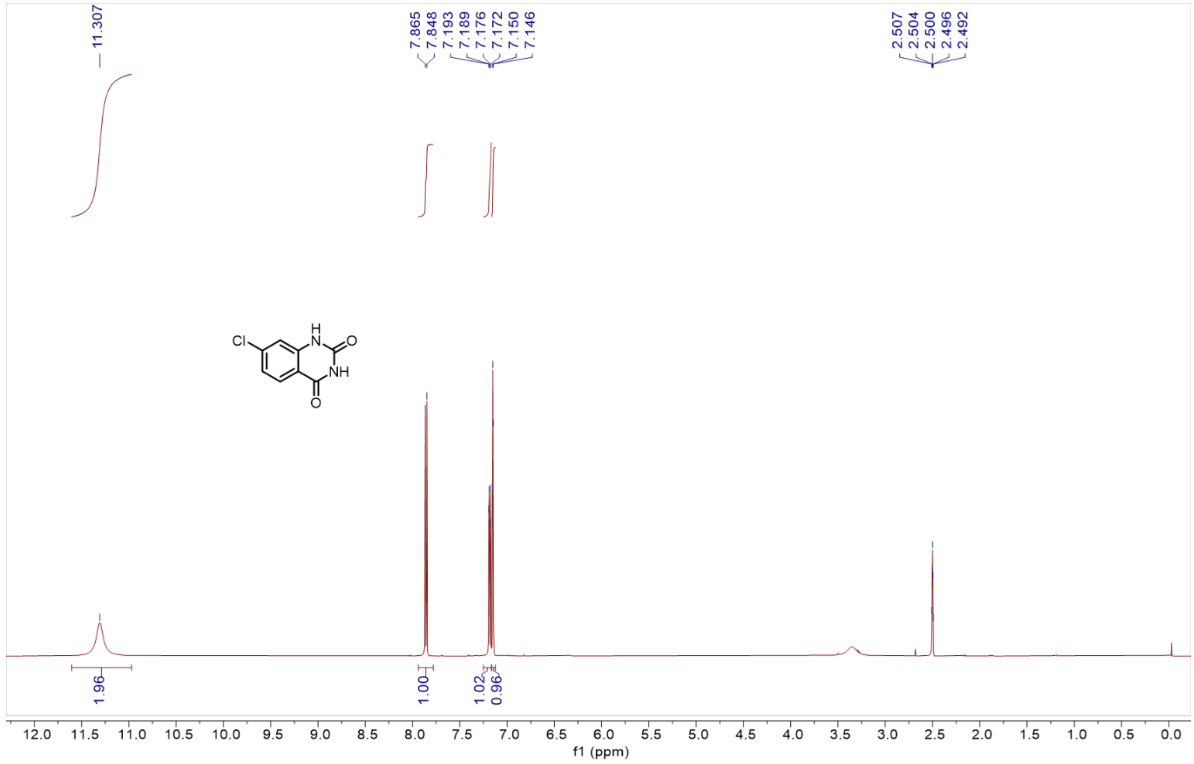

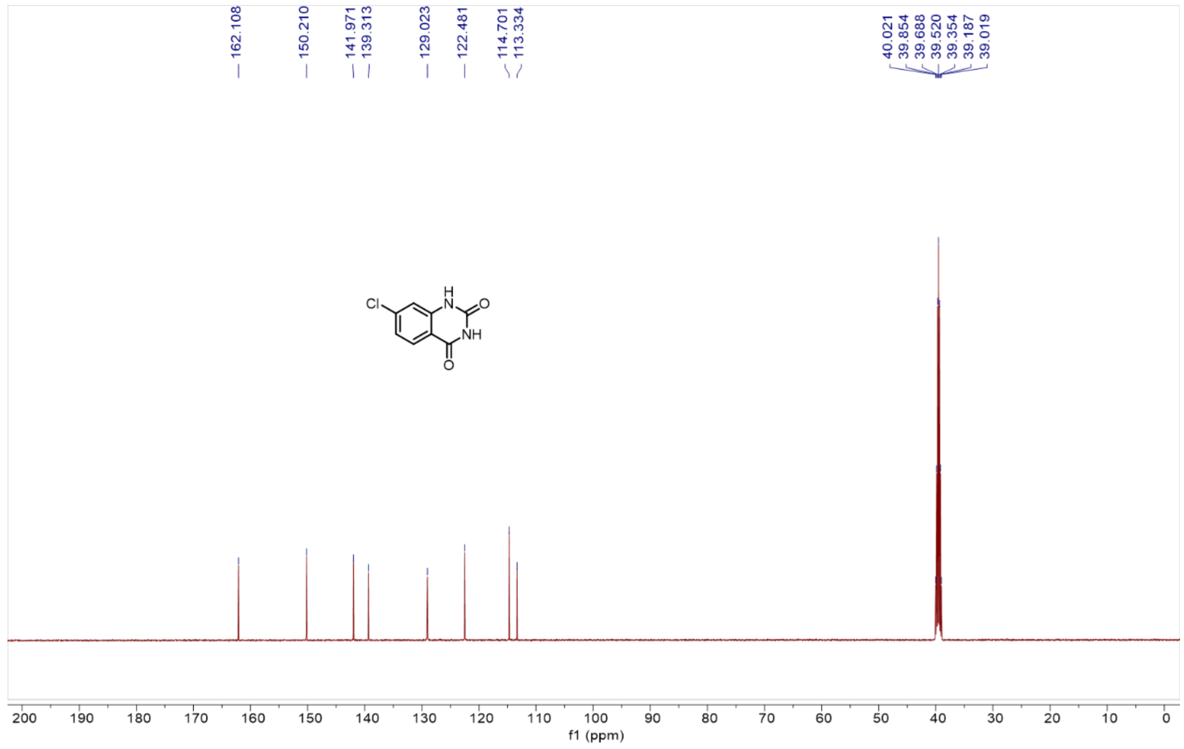

^{1}H NMR of **4h** (600 MHz, DMSO-*d*6) and ^{13}C NMR of **4h** (151 MHz, DMSO-*d*6)

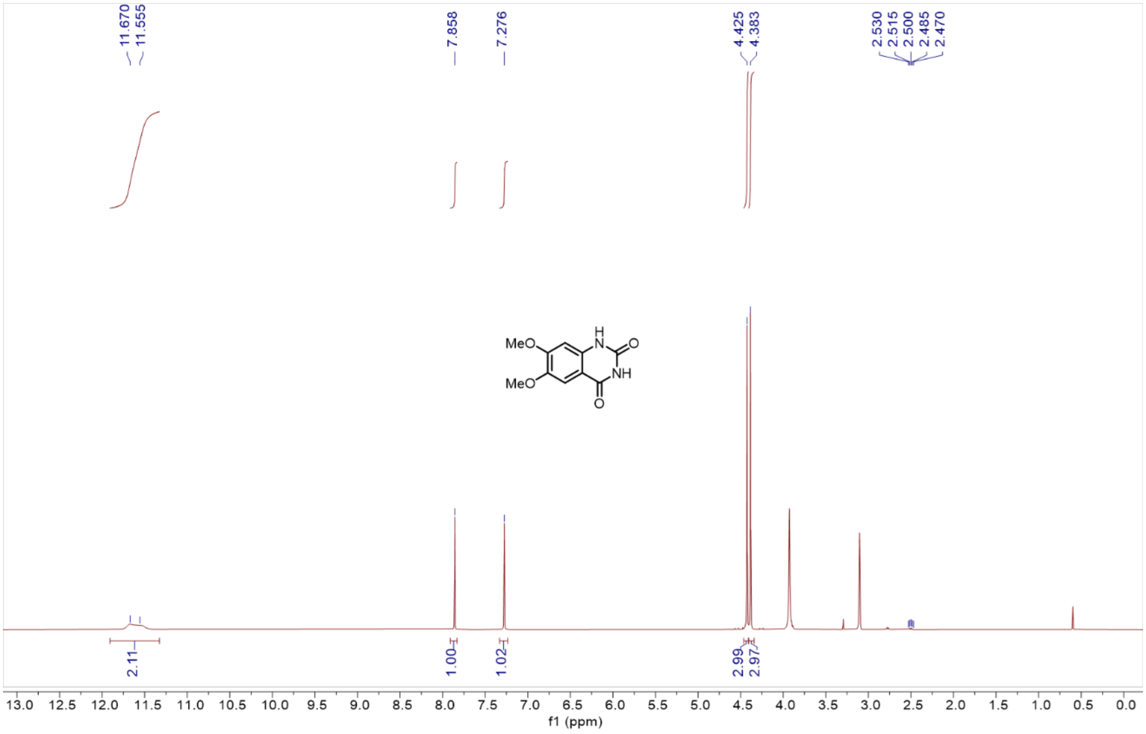

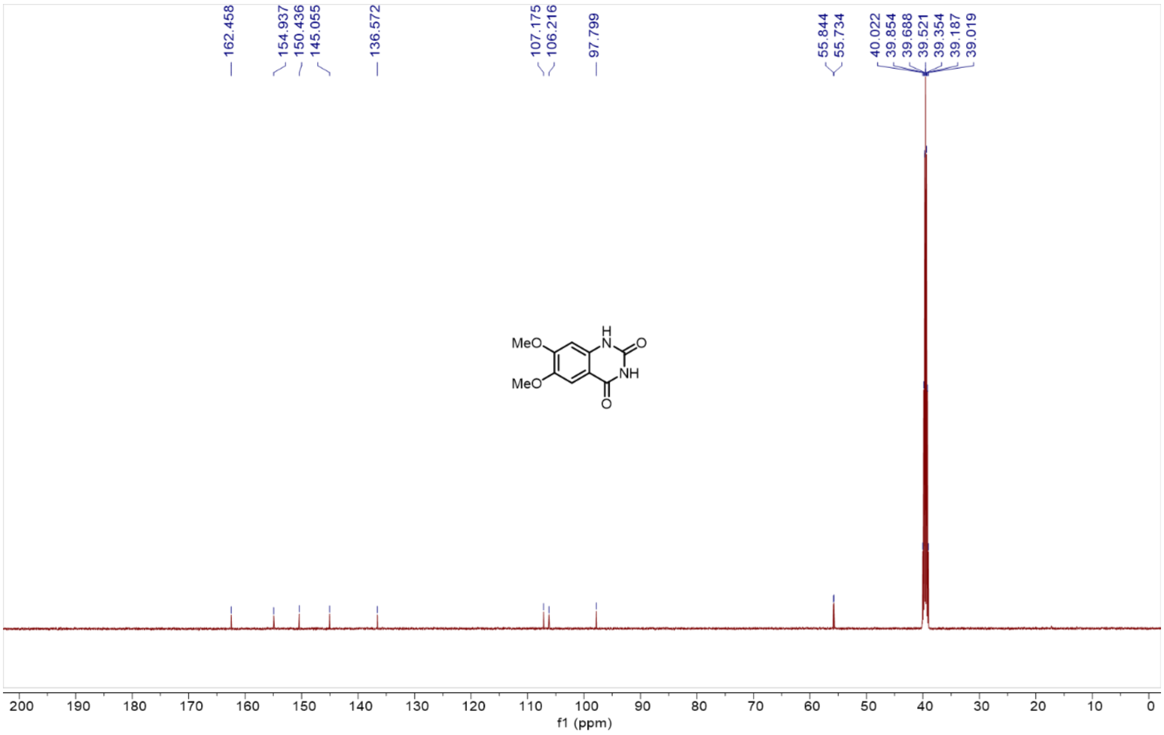

^{1}H NMR of **6a** (600 MHz, DMSO-*d*6) and ^{13}C NMR of **6a** (151 MHz, DMSO-*d*6)

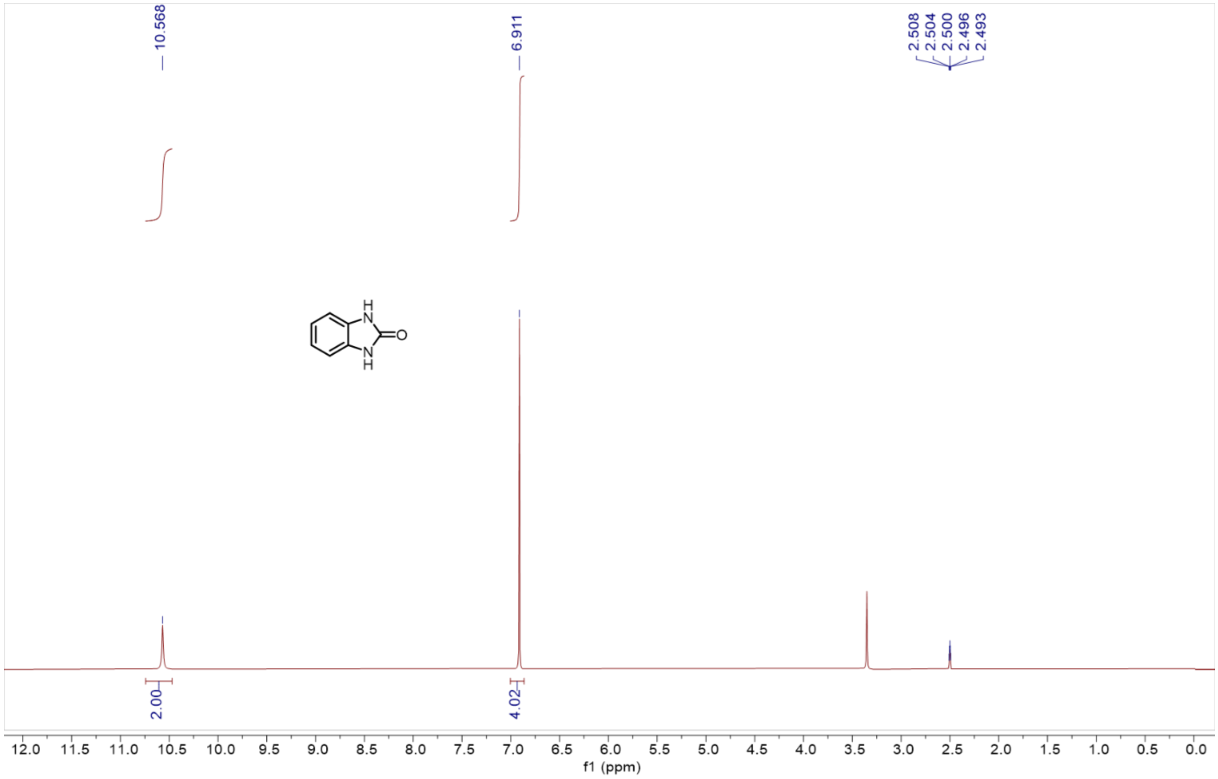

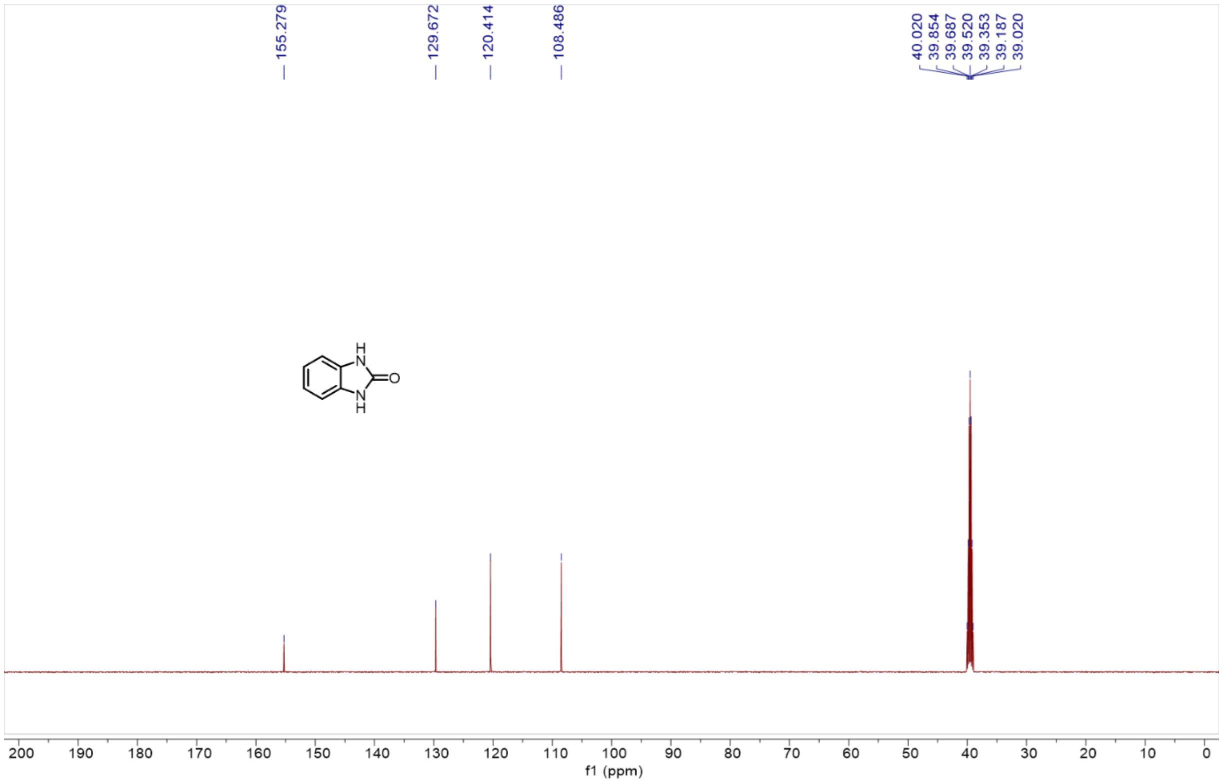

^{1}H NMR of **6b** (600 MHz, $CDCl_3$) and ^{13}C NMR of **6b** (151 MHz, $CDCl_3$)

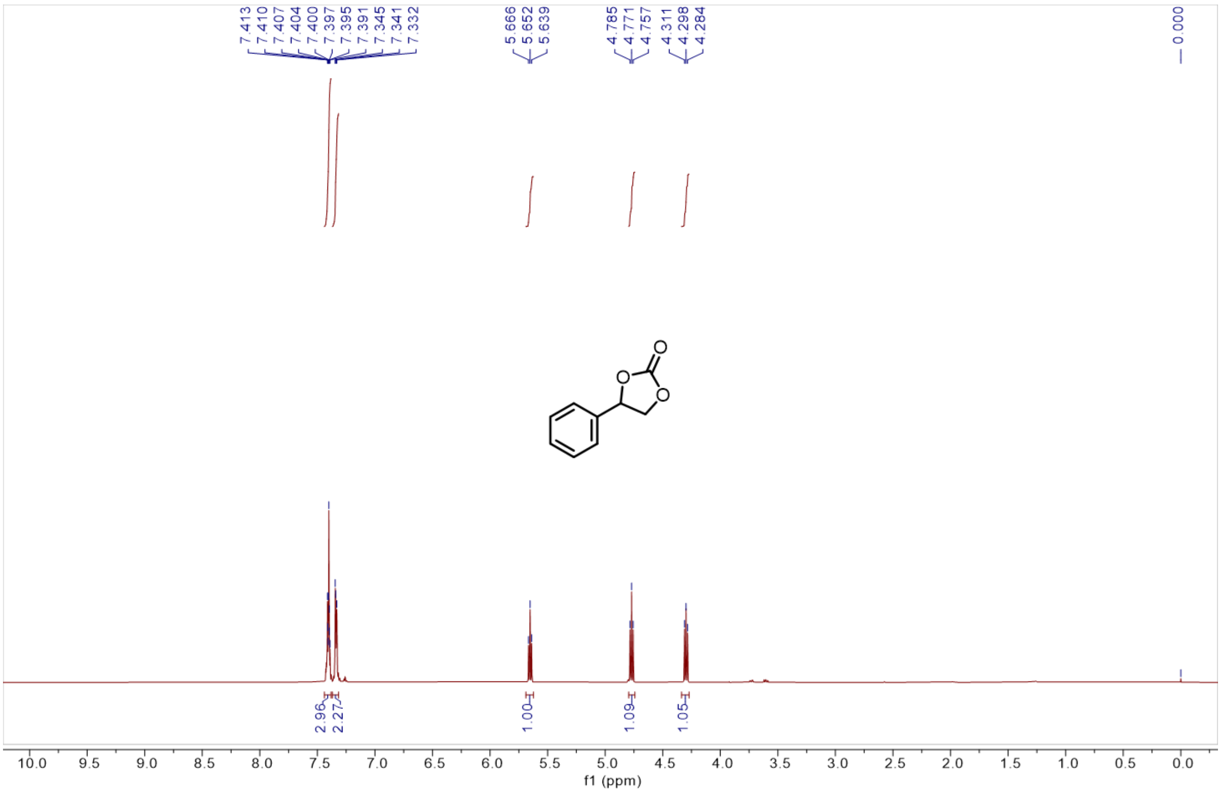

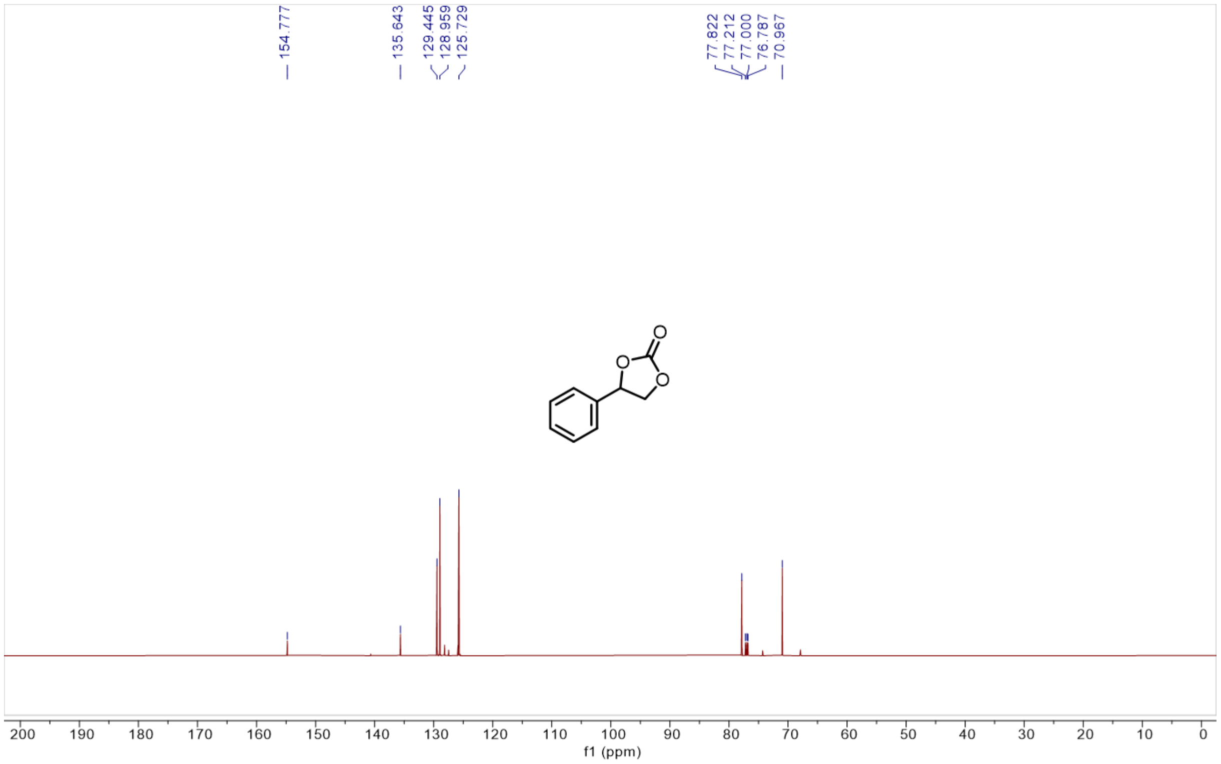

10. DFT calculations

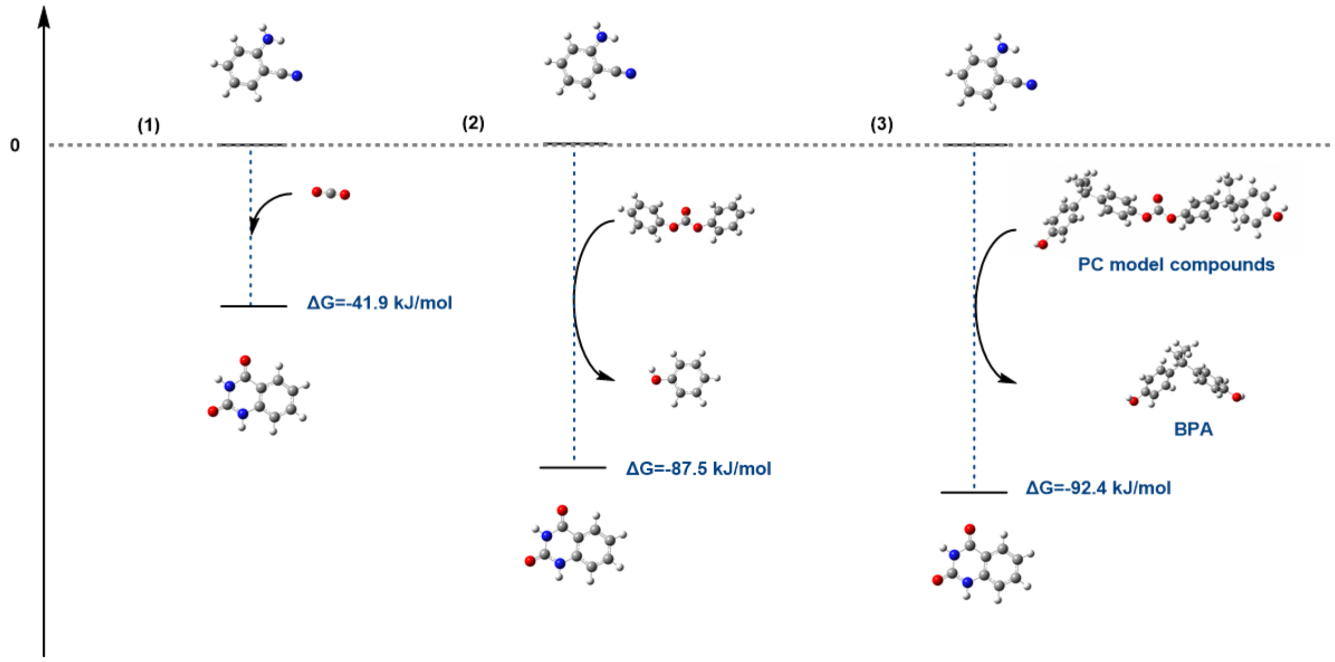

Figure S27. Gibbs free energy change of 2-aminobenzonitrile to quinazolin-2,4(1*H*,3*H*)-dione with different CO_2 sources.

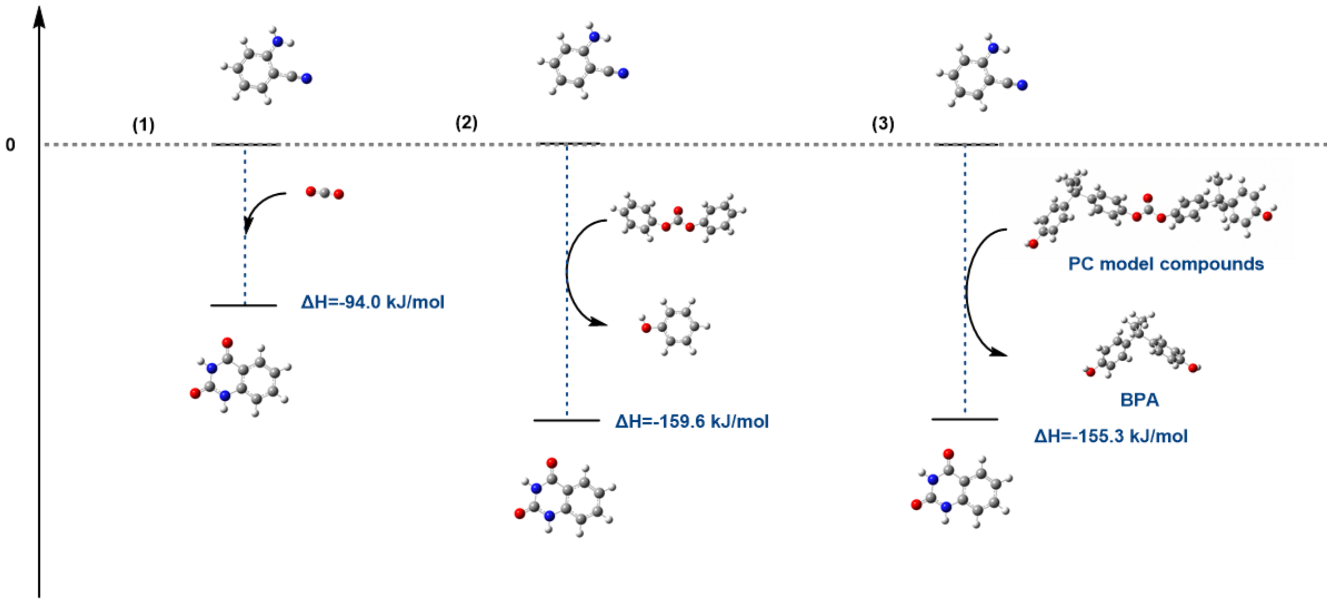

Figure S28. Enthalpy change of reaction from 2-aminobenzonitrile to quinazolin-2,4(1*H*,3*H*)-dione with different CO_2 sources.

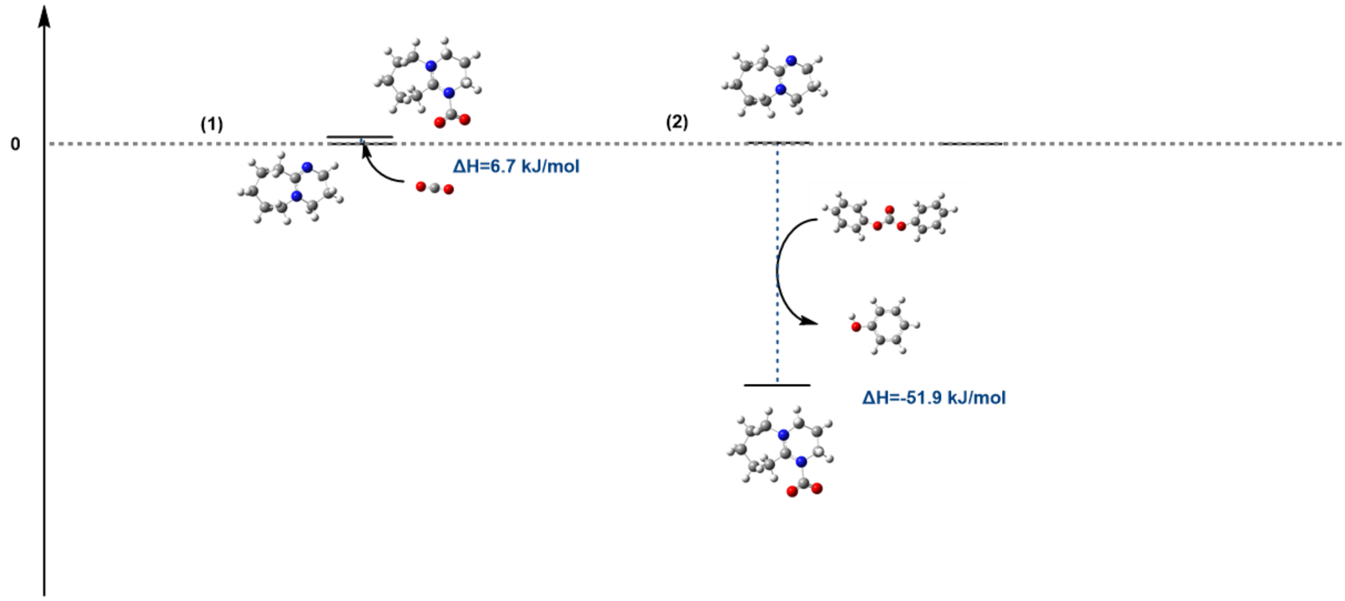

Figure S29. Gibbs free energy change of the transformation from CO_2 or DPC to DBU–CO_2.

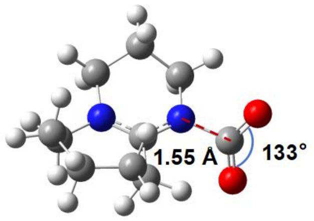

DBU-CO_2

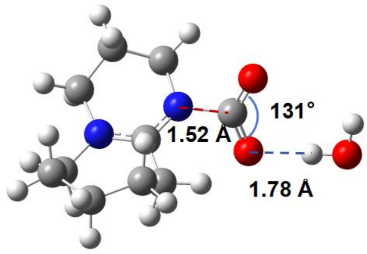

DBU-CO_2---H_2O

Figure S30. The stabilizing effect of water molecules on the DBU-CO_2 intermediate. Optimized geometry of DBU–CO_2 adduct with a water molecule at B3LYP/6-311+G** level of theory.

DFT calculations reveal that, in the absence of water molecules, the C-O bond length between CO_2 and DBU is 1.55 Å, with an O-C-O bond angle of 133°. Upon the addition of H_2O, a hydrogen bond forms between the H atom of H_2O and the O of the DBU-CO_2 complex, resulting in a C-O bond length contraction to 1.52 Å and a reduction in the O-C-O bond angle to 131°. This interaction stabilizes the DBU-CO_2 complex, effectively enhancing the stability of the intermediate.

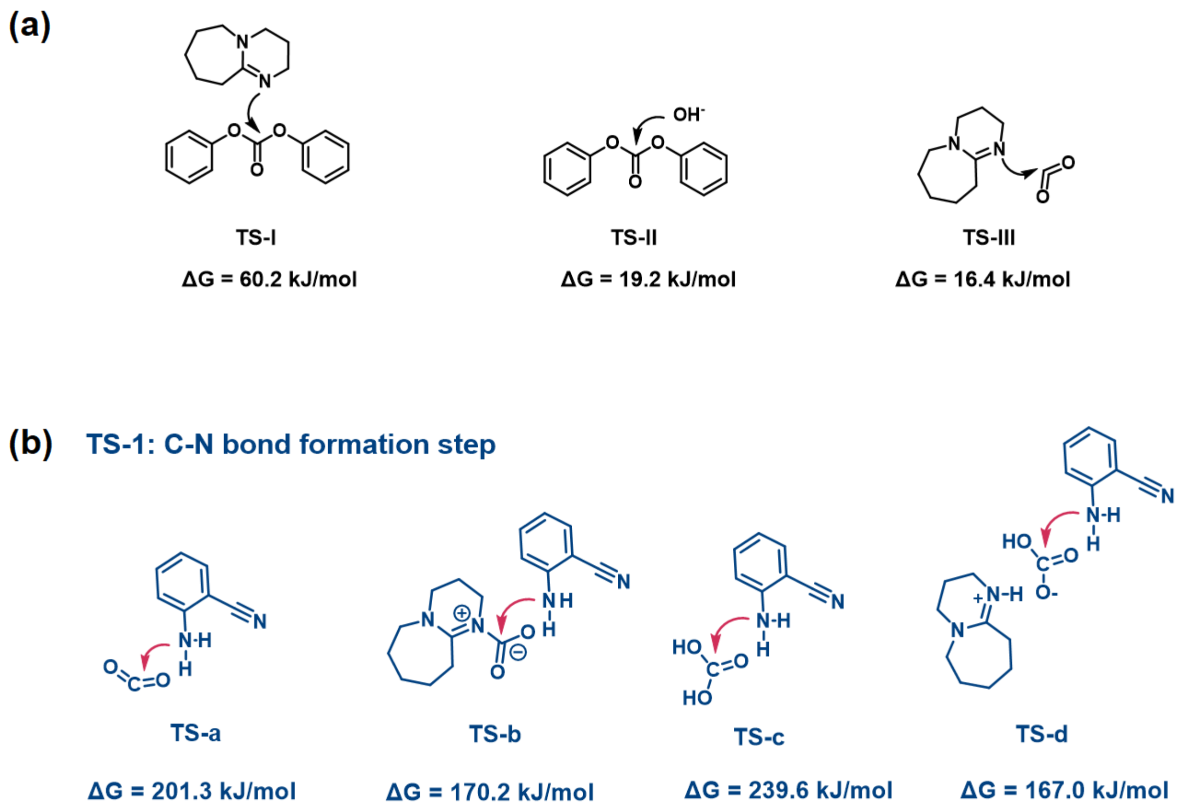

Figure S31. The reaction energy barriers for different key steps. (a) The transition states (TS) for the hydrolysis of the PC model compound and the formation of the DBU-CO_2 intermediate; (b) The energy barriers for the different pathways of C-N bond formation between the amino group of 2-aminobenzonitrile and CO_2 in various forms.

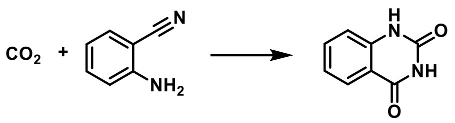

a **b**

System **a**

C	33.84191100	-0.00513200	0.05617700
O	33.24907500	0.69947500	0.76286700
O	34.43474900	-0.70973900	-0.65051100
C	-12.30201600	-2.23845700	0.25711800
C	-12.86783800	-1.21029400	0.99205900
C	-12.58344300	0.13776200	0.69314900
C	-11.69796200	0.39651400	-0.38665000
C	-11.13097600	-0.66183900	-1.12343200
C	-11.42806800	-1.97576900	-0.80829500
H	-12.54104500	-3.26430800	0.51619000
H	-13.54084100	-1.42939000	1.81402000
H	-10.45876300	-0.42639100	-1.94013500
H	-10.99007700	-2.78731300	-1.37618700
C	-11.38119800	1.73813500	-0.73260100
N	-11.13491000	2.83792900	-1.00121000
N	-13.18669800	1.15067600	1.39471700
H	-13.61225400	0.90847300	2.27875300
H	-12.77882600	2.07529900	1.36880800

System **b**

C	2.78515200	-1.03948700	0.00000600
C	1.53108500	-1.63151600	-0.00001200
C	0.38692900	-0.82201800	-0.00001400
C	0.51583800	0.57773000	0.00000000
C	1.79302200	1.15614500	0.00001800
C	2.92432600	0.35625100	0.00002100
H	3.66790200	-1.66926500	0.00000800
H	1.42282700	-2.71041400	-0.00002400
H	1.87318200	2.23640800	0.00003000
H	3.91084900	0.80417500	0.00003700
C	-0.69503100	1.41236600	-0.00000100
O	-0.70739700	2.63510900	-0.00001200
N	-1.88833000	0.69538600	0.00001900
H	-2.74588900	1.23816100	0.00002600
C	-2.05331400	-0.68356600	-0.00000300
N	-0.88004500	-1.39049400	-0.00002900
H	-0.96575900	-2.39983600	-0.00005800
O	-3.15666800	-1.20872200	0.00000800

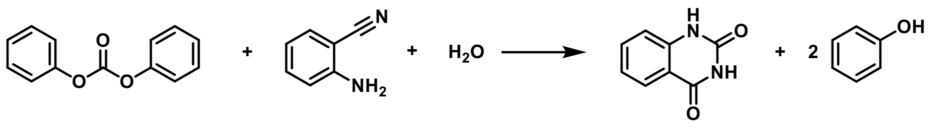

System **c**

O	90.48764700	9.93094700	-1.29186200
H	91.41636000	9.84550700	-1.04263200
H	90.30970200	10.87810500	-1.23573100
C	35.18541100	-1.80691100	1.55969400
C	34.02132400	-1.34787300	0.96704000
C	33.16135400	-2.22706900	0.27782700
C	33.53618700	-3.59557500	0.21563700
C	34.72416200	-4.04533600	0.82508300
C	35.54950400	-3.16016200	1.49528200
H	35.82244700	-1.10267900	2.08400400
H	33.75197500	-0.29874400	1.02604500
H	34.97758600	-5.09684400	0.75856400
H	36.46161600	-3.50954600	1.96318000
C	32.70904200	-4.52495800	-0.47181100
N	32.02525600	-5.27055100	-1.03653500
N	32.03620900	-1.75253300	-0.34676900
H	31.29753600	-2.39870400	-0.58857800
H	31.71326100	-0.83358100	-0.07744700
C	-28.67915100	4.22773800	1.86744700
C	-27.60720400	3.44207500	1.44355200
C	-27.60552400	2.96761000	0.13905200

C	-28.62851900	3.25516200	-0.75414400
C	-29.69488300	4.04158100	-0.31793000
C	-29.72178800	4.52716900	0.98991300
H	-28.69519400	4.60602100	2.88328400
H	-26.78680500	3.20282500	2.11023300
H	-28.58907900	2.87343900	-1.76779200
H	-30.50175000	4.27506300	-1.00331200
H	-30.55184200	5.13963800	1.32352700
O	-26.48888200	2.23885100	-0.31649100
C	-26.53387500	0.89971000	-0.19912600
O	-25.37508700	0.43117100	-0.69576100
O	-27.43349900	0.24948000	0.25916100
C	-25.17862600	-0.96416000	-0.67938000
C	-25.51037800	-1.69761000	-1.81019400
C	-24.58614500	-1.54683700	0.43270100
C	-25.24312700	-3.06678300	-1.82102500
H	-25.96564000	-1.20674000	-2.66255200
C	-24.32474600	-2.91679900	0.40959700
H	-24.33423500	-0.94136600	1.29571000
C	-24.65209900	-3.67659400	-0.71405500
H	-25.49600900	-3.65325900	-2.69707700
H	-23.86234600	-3.38675300	1.27020700
H	-24.44478500	-4.74064700	-0.72782300

System **d**

C	-5.09731400	10.99219200	-0.57504400
C	-4.08192600	11.78138200	-0.05630200
C	-2.79335900	11.24635400	0.07686600
C	-2.53883900	9.92018400	-0.31324400
C	-3.57944400	9.13936000	-0.83542000
C	-4.85322900	9.66795000	-0.96757800
H	-6.09284300	11.40978900	-0.67673400
H	-4.26974400	12.80556300	0.24616600
H	-3.36536700	8.11942500	-1.13097400
H	-5.65663700	9.06353100	-1.37135300
C	-1.18161100	9.37393600	-0.16547000
O	-0.84215600	8.23891200	-0.46804800
N	-0.25638600	10.26775600	0.36594400
H	0.69169500	9.92471100	0.48039200
C	-0.47020600	11.58288900	0.75823300
N	-1.75928300	12.01650200	0.59290100
H	-1.94873200	12.97244400	0.87003600
O	0.42357300	12.28243800	1.21094100
C	-13.62390600	-11.92594800	-0.10496000
C	-14.56907000	-11.42575800	-0.99764200
C	-15.63595800	-10.66270700	-0.51620200
C	-15.75428600	-10.40262400	0.85160500
C	-14.80090900	-10.90958500	1.73464200
C	-13.73207600	-11.67257500	1.26429600
H	-12.79774900	-12.51789100	-0.48416500
H	-14.49021000	-11.61946800	-2.06151800

H	-16.58553500	-9.80881000	1.22029900
H	-14.89890600	-10.70385700	2.79517400
H	-12.99362100	-12.06472200	1.95413500
O	-16.53968600	-10.19262200	-1.42961400
H	-17.22504400	-9.68316700	-0.97550900
C	19.58849300	-6.66752000	1.36153400
C	18.30825200	-7.11997400	1.05077000
C	17.84991300	-7.04079800	-0.26679300
C	18.66994000	-6.51100500	-1.26655100
C	19.94986900	-6.06158400	-0.94169600
C	20.41711600	-6.13639600	0.37052800
H	19.93917700	-6.73147200	2.38604300
H	17.66053300	-7.53316400	1.81583300
H	18.30884800	-6.45180200	-2.28916200
H	20.58131500	-5.65162200	-1.72261400
H	21.41253600	-5.78611800	0.61836700
O	16.58624200	-7.49756800	-0.52463100
H	16.38749000	-7.38909400	-1.46495400

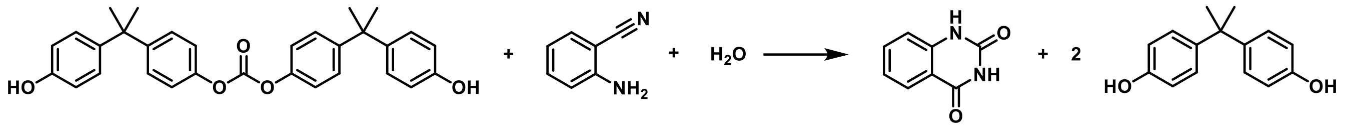

System **e**

O	-93.18913800	12.84559800	9.43334400
H	-93.44029300	11.93738900	9.22358000
H	-92.25016200	12.79136300	9.65083800
C	13.45979600	7.60282500	-1.01794400
C	14.11581300	7.37212300	-2.23836800
C	15.19786000	8.14130000	-2.64975100
C	15.65973100	9.17855600	-1.83546600
C	15.02844400	9.42952200	-0.61968200
C	13.94117400	8.64596100	-0.22246100
H	13.77916000	6.56876100	-2.88504800
H	15.69069700	7.94377500	-3.59539800
H	15.38104500	10.23292600	0.02073400
H	13.47679700	8.87106200	0.72917800
C	12.23915800	6.74159300	-0.63324500
C	11.09214800	7.07468700	-1.61421800
H	10.17790300	6.53265500	-1.35617800
H	10.87096900	8.14388700	-1.56100300
H	11.35172200	6.83780500	-2.64864500
C	11.73087600	7.05051700	0.79381400
H	10.88681600	6.39671900	1.02877500
H	12.50161500	6.88858700	1.55088100

H	11.38321500	8.08425500	0.87265700
C	12.63062400	5.24963500	-0.67613500
C	11.92236500	4.29772700	-1.41695100
C	13.72647400	4.80435900	0.08057000
C	12.28703600	2.94879600	-1.40700000
H	11.07324100	4.58987900	-2.02058700
C	14.10194400	3.46509200	0.10448500
H	14.30557400	5.51572300	0.65855500
C	13.36943800	2.55137300	-0.64195000
H	11.73454900	2.22116600	-1.99057500
H	14.95286700	3.13486800	0.68943600
O	13.79028300	1.20696100	-0.68858800
C	13.27421100	0.36351200	0.22297400
O	13.81811300	-0.84268000	-0.01788400
O	12.48320100	0.63489700	1.08539900
C	13.41808600	-1.91287100	0.80746500
C	12.29978200	-2.65976300	0.45998400
C	14.19820000	-2.24916700	1.89962700
C	11.96249600	-3.76272200	1.23744200
H	11.70722300	-2.38558900	-0.40547800
C	13.84364500	-3.35973500	2.67038100
H	15.07281100	-1.65889600	2.14803500
C	12.72301500	-4.13586000	2.35746700
H	11.09317500	-4.34767700	0.95888600
H	14.46546900	-3.60794900	3.52036300
C	12.27899800	-5.34822400	3.20257500

C	13.29815100	-5.69276600	4.31225500
H	13.40129900	-4.87095400	5.02647000
H	12.94916600	-6.57011900	4.86305600
H	14.28672200	-5.92262700	3.90805600
C	10.95001300	-4.97265600	3.89711000
H	10.60997800	-5.77607100	4.55638200
H	11.09971800	-4.07970800	4.50974600
H	10.15495800	-4.75749100	3.17958400
C	12.13302100	-6.58255800	2.28881900
C	13.22236800	-6.99879200	1.50577800
C	10.96619500	-7.34807800	2.21633400
C	13.15676400	-8.12272700	0.69100500
H	14.14619500	-6.43044200	1.52593500
C	10.88154200	-8.48087200	1.40153300
H	10.09298600	-7.07603000	2.79568400
C	11.97829000	-8.87142200	0.63697600
H	14.00988400	-8.42474600	0.09345200
H	9.95998500	-9.05491800	1.36498700
O	11.95794600	-9.96991000	-0.18083800
H	11.09084500	-10.39501000	-0.12927700
O	16.73041100	9.90904300	-2.27800300
H	16.94924000	10.58883900	-1.62593200
C	-40.95163000	-4.52040900	-3.13047100
C	-39.81699700	-5.31253000	-3.08486400
C	-38.52913000	-4.74104400	-3.13740400
C	-38.44397300	-3.32693300	-3.23978500

C	-39.60926300	-2.53633200	-3.28495000
C	-40.86042900	-3.12408000	-3.23134300
H	-41.92649000	-4.99389800	-3.08511700
H	-39.90328500	-6.39099700	-3.00686500
H	-39.50526500	-1.46039900	-3.36209300
H	-41.75416800	-2.51331800	-3.26527000
C	-37.17132800	-2.69711300	-3.30134300
N	-36.12649700	-2.19887700	-3.35129300
N	-37.41402600	-5.53935100	-3.14002200
H	-36.51704300	-5.13567900	-2.90810100
H	-37.53143900	-6.49633800	-2.83752600

System **e**

C	22.28447000	-4.72130000	-0.11558100
C	23.47710900	-4.51922600	0.58432300
C	24.42388400	-3.57935700	0.16581500
C	24.19024400	-2.81732200	-0.97597400
C	23.00540400	-2.99909800	-1.69393100
C	22.07481300	-3.93654900	-1.26184300
H	23.69542400	-5.09212700	1.47664300
H	25.34070700	-3.44324100	0.73230000
H	22.82129500	-2.40349200	-2.58136200
H	21.15927200	-4.05420100	-1.83169900
C	21.22963900	-5.77129400	0.29235000
C	21.58120200	-6.46266000	1.62944500

H	20.79643200	-7.18010600	1.88388300
H	22.52370000	-7.01278300	1.55571400
H	21.66368900	-5.75082100	2.45405500
C	21.20273100	-6.86766200	-0.79696900
H	20.50495100	-7.66861700	-0.53624500
H	20.91995700	-6.47176100	-1.77512100
H	22.19793200	-7.31055300	-0.89044800
C	19.85879600	-5.08228100	0.45856500
C	19.72712300	-4.00539700	1.35105600
C	18.70723100	-5.49100400	-0.21962000
C	18.51049700	-3.36788700	1.56287300
H	20.59657100	-3.64890900	1.89305400
C	17.47523000	-4.86032900	-0.02105700
H	18.74591100	-6.31390100	-0.92209000
C	17.37405600	-3.79700300	0.87239200
H	18.43298000	-2.53759100	2.25629300
H	16.59799500	-5.19920300	-0.56490900
O	16.19643500	-3.13872300	1.11016100
H	15.49429200	-3.52872900	0.57159200
O	25.07832800	-1.88121300	-1.43604200
H	25.84854900	-1.85277200	-0.85199600
C	-19.98921400	-4.70969700	1.39626600
C	-20.88415100	-4.41829900	2.42936300
C	-21.11793700	-3.10560700	2.85032600
C	-20.44970200	-2.04898100	2.23694100
C	-19.55021200	-2.31269500	1.20074900

C	-19.33213100	-3.62367500	0.79425700
H	-21.42286500	-5.21224400	2.93082300
H	-21.82186500	-2.91111300	3.65458500
H	-19.03230400	-1.48984500	0.72031100
H	-18.63466200	-3.80205900	-0.01720700
C	-19.67704900	-6.14562400	0.92512700
C	-20.56754500	-7.19621800	1.62709200
H	-20.32272700	-8.19154000	1.24669300
H	-20.39790500	-7.20194800	2.70764200
H	-21.63101800	-7.02192400	1.44781300
C	-18.21132000	-6.46199100	1.30072400
H	-17.94740300	-7.49037500	1.03790700
H	-17.50426700	-5.79029200	0.80834800
H	-18.08161500	-6.35115600	2.38064800
C	-19.92484100	-6.25249300	-0.59419300
C	-21.18006300	-5.90231600	-1.11890000
C	-18.96481300	-6.71699000	-1.49731200
C	-21.46858200	-6.00987000	-2.47419800
H	-21.95330100	-5.52986500	-0.45539000
C	-19.23639600	-6.83038400	-2.86412000
H	-17.97837400	-7.00135500	-1.15355800
C	-20.49045000	-6.47714900	-3.35590000
H	-22.44442700	-5.73163600	-2.85702500
H	-18.46847400	-7.19297100	-3.54161600
O	-20.81693900	-6.56422400	-4.68355500
H	-20.05746700	-6.89669800	-5.18137600

O	-20.63515000	-0.74208400	2.60354400
H	-21.27875600	-0.69722600	3.32390900
C	-4.11382900	14.02220200	-0.71445300
C	-3.08044500	14.85758500	-0.31799800
C	-1.78727000	14.33528400	-0.17925700
C	-1.54558300	12.97586700	-0.44264300
C	-2.60433100	12.14860000	-0.84239000
C	-3.88307100	12.66413800	-0.97865500
H	-5.11307600	14.42975400	-0.82032300
H	-3.25774600	15.90769400	-0.11396800
H	-2.40009800	11.10341900	-1.04077200
H	-4.70063400	12.02373700	-1.28736000
C	-0.18233300	12.44454100	-0.29363300
O	0.14730300	11.28451100	-0.49519400
N	0.76088200	13.38496700	0.11120600
H	1.71377100	13.05430200	0.22251100
C	0.55885000	14.73265700	0.37926600
N	-0.73559800	15.15088500	0.21678600
H	-0.91560700	16.13009500	0.40462500
O	1.46678400	15.47148400	0.72950600

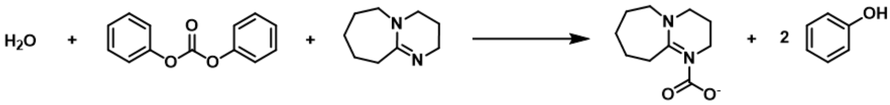

f g

System **f**

C	16.51275900	-5.04455400	-0.41297600
C	14.13088400	-4.55543200	-1.20890800
H	13.38528100	-5.02036000	-1.86149700
H	13.85235200	-4.82012300	-0.18212900
C	14.06055000	-3.03783500	-1.39787000
H	13.04756900	-2.69546900	-1.16050800
H	14.22547900	-2.81057400	-2.45896500
C	15.07041200	-2.23725200	-0.57229400
H	14.88298700	-2.36967100	0.49965800
H	14.93421700	-1.17223900	-0.78917800
N	16.80006700	-6.11911400	0.25437900
N	17.02167300	-3.79457300	-0.17735400
C	17.72923100	-6.01151200	1.38006500
H	18.26481200	-6.96130100	1.47369900
H	17.15476100	-5.88861600	2.30891500
C	18.71488200	-4.85653500	1.23548700
H	19.40351900	-5.06087300	0.40869200
H	19.31108600	-4.73289700	2.14334300
C	17.94114000	-3.58016500	0.94423000
H	18.62071200	-2.76908600	0.67352700
H	17.38432600	-3.25981500	1.83485500
C	16.53195100	-2.59751600	-0.86779100

H	17.17831300	-1.78039700	-0.54711400
H	16.67488400	-2.70420300	-1.94848900
C	15.50338000	-5.17842100	-1.53296800
H	15.89531400	-4.74085000	-2.45743500
H	15.38439200	-6.24830100	-1.70332000
O	35.54632500	22.68154700	0.03405000
H	36.21213500	22.78687900	0.72508300
H	34.74549900	22.42586700	0.50863000
C	-18.67346200	2.26906900	-2.60531600
C	-17.65930600	1.46109700	-2.09291700
C	-17.64628200	1.19595200	-0.73149100
C	-18.60651100	1.70237200	0.13148600
C	-19.61600300	2.50879100	-0.39301400
C	-19.65023600	2.79262900	-1.75816300
H	-18.69860500	2.48625900	-3.66674600
H	-16.89230100	1.04443700	-2.73482600
H	-18.56289200	1.46936100	1.18863700
H	-20.37445400	2.91261200	0.26739900
H	-20.43728000	3.41917200	-2.16133200
O	-16.66420300	0.32863400	-0.21674100
C	-15.50402900	0.88752600	0.16749100
O	-14.72157800	-0.11118500	0.61064400
O	-15.22183600	2.05389300	0.12494600
C	-13.43646300	0.24305900	1.06298700
C	-13.25174700	0.49806500	2.41359600
C	-12.38808400	0.26024300	0.15498000

C	-11.96492600	0.78362900	2.86855600
H	-14.09710400	0.47355900	3.09085200
C	-11.10617200	0.54751600	0.62197200
H	-12.57346000	0.05390800	-0.89241300
C	-10.89368200	0.80863100	1.97567400
H	-11.80270300	0.98479600	3.92102500
H	-10.27533300	0.56474100	-0.07366800
H	-9.89506900	1.02973600	2.33403300

System **g**

O	13.30015700	-3.38551400	2.34598200
C	12.97669100	-3.33349200	1.15711400
O	12.19503500	-3.99202400	0.46613600
C	13.21460700	-1.25767700	-0.28579100
C	11.26812600	0.05949800	0.65480200
H	10.22881200	-0.13280200	0.93442500
H	11.85121000	0.02744800	1.58144000
C	11.35763000	1.44557000	0.01378300
H	11.00313000	2.19433200	0.72928800
H	10.67001600	1.47814500	-0.84039600
C	12.75394900	1.83715700	-0.47465600
H	13.44987800	1.91816600	0.36784200
H	12.70052000	2.82715100	-0.93825700
N	13.77025600	-2.25463600	0.38794300
N	13.94874600	-0.33744600	-0.92000300
C	15.22921900	-2.34385000	0.55656100

H	15.47990400	-3.39146400	0.71680200
H	15.52330500	-1.78593000	1.44972500
C	15.91732300	-1.79592400	-0.67964000
H	15.70013700	-2.43862600	-1.53748000
H	16.99857200	-1.78142800	-0.53265100
C	15.42108000	-0.38842800	-0.94657500
H	15.74070100	-0.04459300	-1.93085500
H	15.81059900	0.31284900	-0.20144200
C	13.34343300	0.87412100	-1.50855400
H	14.14676300	1.36593700	-2.05383100
H	12.58851500	0.58441300	-2.24381200
C	11.71868700	-1.09239100	-0.27016700
H	11.36616300	-0.91282400	-1.28888400
H	11.27987200	-2.02796400	0.05787400
C	-10.65662900	10.65715900	-0.05345900
C	-10.17909200	11.88765000	-0.50269400
C	-10.88176000	13.05458000	-0.19381800
C	-12.05634200	12.99051600	0.55877600
C	-12.52233200	11.75517400	1.00093100
C	-11.82799300	10.58210600	0.69923700
H	-10.10625800	9.75501300	-0.29638300
H	-9.26701600	11.94300700	-1.08902100
H	-12.59075400	13.90471100	0.78978100
H	-13.43465200	11.71130900	1.58537900
H	-12.19527000	9.62360300	1.04630900
C	-17.51987500	-10.79307200	0.59660600

C	-16.51553800	-10.66846600	-0.36562800
C	-16.29673200	-9.44364100	-1.00027100
C	-17.08684900	-8.34442200	-0.66658200
C	-18.09222300	-8.45802900	0.29290300
C	-18.30186500	-9.68747200	0.92039500
H	-17.67743300	-11.75019600	1.08007500
H	-15.51465100	-9.35261400	-1.74781200
H	-16.91161600	-7.39635700	-1.16281700
H	-18.70385600	-7.60104700	0.54904800
H	-19.08027400	-9.78966300	1.66841300
O	-15.77232100	-11.78064900	-0.65052000
H	-15.11581600	-11.56560700	-1.32725300
O	-10.46171500	14.28943300	-0.60503400
H	-9.64385900	14.19964300	-1.11322500

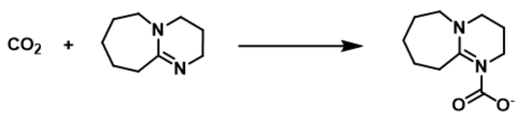

System **h**

C	3.45464600	-0.74619000	-0.31010200
C	1.06460000	-0.58508900	0.58940000
H	0.12681700	-1.14186600	0.49705100
H	1.51851600	-0.89704200	1.53731600
C	0.73836900	0.91004800	0.62991100
H	0.06872500	1.10658500	1.47410400
H	0.17895600	1.16977500	-0.27803400
C	1.95508600	1.83337800	0.72550900
H	2.49482200	1.66345600	1.66442400
H	1.60609900	2.87150200	0.74338900
N	4.20313000	-1.77765400	-0.06833800
N	3.86923900	0.55946500	-0.32453900
C	5.61485100	-1.55325100	0.24566700
H	6.18416700	-2.42297500	-0.09653700
H	5.73822400	-1.51392300	1.33709300
C	6.17017600	-0.27544400	-0.37502600
H	6.20409500	-0.38007800	-1.46474100
H	7.18774600	-0.07933400	-0.02713000
C	5.26249600	0.88802400	-0.00742600
H	5.53506900	1.78334500	-0.57079100
H	5.36179200	1.12743900	1.05964600
C	2.93693200	1.68475000	-0.44376500

H	3.55461200	2.58026100	-0.51247800
H	2.38252100	1.61372100	-1.38579000
C	1.98674500	-1.00702200	-0.57169800
H	1.66639600	-0.50857000	-1.49292600
H	1.89747700	-2.08104700	-0.73358100
C	-12.87799500	-0.14815300	-0.08617300
O	-12.82146100	0.48009000	-1.06055700
O	-12.93454600	-0.77636900	0.88822400

System **i**

O	-1.72926700	-2.15469000	-0.73491400
C	-2.20925800	-1.23372100	-0.07028400
O	-3.27515800	-1.09573400	0.53476300
C	-0.04426900	0.02308600	0.30127200
C	1.56305600	-1.89920700	-0.17262700
H	1.64572200	-2.95595600	0.09560400
H	1.13262400	-1.86514600	-1.17694500
C	2.95933900	-1.27494900	-0.16201400
H	3.59334400	-1.80370200	-0.88094900
H	3.40734700	-1.43350300	0.82680600
C	2.98984700	0.22304800	-0.47226600
H	2.63865200	0.41380900	-1.49250300
H	4.02592800	0.57245300	-0.42271500
N	-1.32938800	0.03992200	-0.02236600
N	0.72483300	1.11234000	0.20239300
C	-2.02693600	1.27270100	-0.42234600

H	-3.02964300	1.21462900	-0.00343300
H	-2.11125900	1.28381600	-1.51399100
C	-1.28687200	2.49899800	0.07334600
H	-1.38355400	2.58209700	1.15972800
H	-1.71585400	3.39690300	-0.37453400
C	0.17936700	2.38506800	-0.29823200
H	0.75893500	3.18933600	0.15257900
H	0.31557700	2.43451300	-1.38365600
C	2.17107100	1.07998600	0.49776500
H	2.50351000	2.11430000	0.44039600
H	2.32615700	0.75145700	1.52914100
C	0.58173700	-1.24384000	0.81987600
H	1.10586200	-1.00120700	1.74901300
H	-0.20855700	-1.94325200	1.06954300

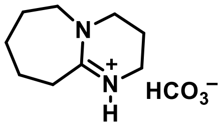

[DBUH+][HCO3-]

O	4.63819000	-0.93188500	-0.01496100
H	3.51501400	-0.61249800	1.89464500
C	3.55973300	-0.32168900	0.00497500
O	2.93802400	-0.17074800	1.25735500
O	2.91251100	0.19915000	-0.93731200
C	-0.60483200	0.24626700	-0.56163300
C	-2.99072800	-0.32164300	-0.46965200
H	-2.95518600	-0.75422200	-1.47269800
H	-3.88820700	0.29298400	-0.41866100
C	-3.04205500	-1.42236800	0.59168500
H	-4.06284100	-1.81667300	0.61281700
H	-2.85293400	-0.97422100	1.57356400
C	-2.07166400	-2.57615900	0.32826000
H	-2.18240400	-3.32415100	1.11975000
H	-2.36087200	-3.06727800	-0.60908800
C	-0.59798400	-2.17382700	0.23184700
H	-0.00135200	-3.07241000	0.05283400
H	-0.25472100	-1.75586900	1.18401800
C	-0.27872900	-1.18057800	-0.90367200
H	0.78365000	-1.22013900	-1.14157200
H	-0.82434900	-1.46231500	-1.80977800
N	0.39681600	1.10155900	-0.49573100
N	-1.85826900	0.60732200	-0.30265500
C	-2.18234100	1.96804000	0.16683300
H	-2.54688900	2.55402800	-0.68277100
H	-2.99564800	1.87593500	0.88755700
C	-0.97237100	2.62926000	0.80801700
H	-0.74988000	2.14445600	1.76279900
H	-1.19384400	3.67935100	1.00402100
C	0.22841900	2.50280000	-0.11722000
H	0.08942200	3.11049200	-1.01707700

H	1.14816700	2.82669400	0.37068500
H	1.36855100	0.74091000	-0.63964000

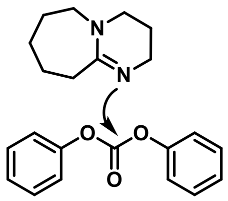

TS-I

C	-0.19354700	1.11119000	0.05381000
C	0.34977600	1.27106200	-1.34268100
H	0.36587000	0.29372900	-1.80694900
H	-0.32922900	1.90170300	-1.92279900
C	1.76878900	1.87806100	-1.36251900
H	2.24161400	1.59572200	-2.30736300
H	2.36770700	1.42121700	-0.56846900
C	1.79026000	3.40209000	-1.23500700
H	2.82794800	3.75067900	-1.26148200
H	1.29287900	3.83386100	-2.11266500
C	1.10894900	3.94754000	0.02222500
H	1.17621700	5.04021800	0.01111300
H	1.63207400	3.60622800	0.92291500
C	-0.37249500	3.57764900	0.14621100
H	-0.85578300	4.26260400	0.84136200
H	-0.87852000	3.69585900	-0.81723900
N	-0.20639600	-0.07372000	0.61775800
N	-0.61875400	2.22879000	0.68358000
C	-1.31722000	2.17935500	1.97803300
H	-0.64215000	2.53629200	2.76357300
H	-2.15958700	2.87293900	1.92267900
C	-1.79457900	0.77094300	2.27713500
H	-2.64489900	0.51437900	1.63919100
H	-2.11900400	0.70510900	3.31769800
C	-0.64808800	-0.19053100	2.00238800
H	-0.93353800	-1.22772400	2.16653800
H	0.19299000	0.03028300	2.67163800
C	3.85237500	-1.38791300	1.56644700
C	2.55762500	-1.51514700	1.06012200

C	2.36764600	-1.53945400	-0.32364400
C	3.46807600	-1.45104300	-1.18288700
C	4.75159900	-1.32830600	-0.66305600
C	4.95324400	-1.29231100	0.71823800
H	3.99331000	-1.36638700	2.64169600
H	1.71872400	-1.60374400	1.73046600
H	3.29852100	-1.46612300	-2.25305300
H	5.59549300	-1.25670400	-1.34027300
H	5.95309000	-1.19392600	1.12474100
O	1.15502500	-1.60542800	-0.95659200
C	-0.04475900	-1.74805800	-0.22964700
O	-1.00623600	-1.59419100	-1.26680500
C	-2.31409200	-1.34942800	-0.90375200
C	-2.94742300	-0.28292600	-1.54260100
C	-3.01193400	-2.13522200	0.01531000
C	-4.27749700	0.01047200	-1.25174500
H	-2.38743900	0.31171400	-2.25373200
C	-4.33967400	-1.82445400	0.30774700
H	-2.51434500	-2.96265000	0.49850000
C	-4.97788500	-0.75432300	-0.31856700
H	-4.76255700	0.84279700	-1.74949200
H	-4.87848700	-2.43057500	1.02783200
H	-6.01083900	-0.52211700	-0.08613600
O	-0.16614900	-2.59617300	0.64102100

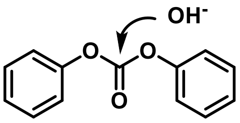

TS-II

C	-4.01293600	1.22925400	-0.40294400
C	-2.71039400	0.96665800	0.01650100
C	-2.34589400	-0.35350300	0.26975600
C	-3.24858300	-1.40115500	0.12280600
C	-4.55195400	-1.12117600	-0.29085400
C	-4.93666700	0.19223100	-0.55774900
H	-4.31231000	2.25251900	-0.60402900
H	-1.94872900	1.72730800	0.18233300
H	-2.93213000	-2.41679900	0.33015000
H	-5.26196400	-1.93242900	-0.40685300
H	-5.94879800	0.40719500	-0.88172800
O	-1.08293500	-0.66501700	0.75581600
C	0.00000000	-0.22051300	0.05117400
O	1.08293500	-0.66501800	0.75581600
C	2.34589400	-0.35350300	0.26975700
C	3.24858100	-1.40115600	0.12280200
C	2.71039500	0.96665800	0.01650700
C	4.55195300	-1.12117600	-0.29085900
H	2.93212800	-2.41680000	0.33014100
C	4.01293700	1.22925500	-0.40293900
H	1.94873000	1.72730800	0.18234200
C	4.93666700	0.19223000	-0.55774900
H	5.26196100	-1.93243000	-0.40686200
H	4.31231200	2.25252000	-0.60402000
H	5.94879800	0.40719500	-0.88172800
O	0.00000100	0.06992300	-1.11811900
O	0.00000000	1.91806200	0.93930000
H	0.00000300	1.83619600	1.90050300

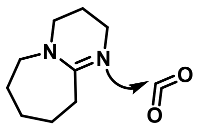

TS-III

C	-0.02499100	0.09659700	0.35433400
C	-0.46730700	-1.25924200	0.85212200
H	0.43042400	-1.81773000	1.10003600
H	-1.04024500	-1.13795100	1.77681500
C	-1.30259600	-2.05145200	-0.17275400
H	-1.23443400	-3.11253500	0.08543900
H	-0.85036500	-1.94363600	-1.16423100
C	-2.77951600	-1.65323300	-0.21970800
H	-3.29469500	-2.27552000	-0.95910200
H	-3.23460900	-1.88234900	0.75232500
C	-3.03631000	-0.17819700	-0.53668000
H	-4.11726400	-0.00256200	-0.53698700
H	-2.67421400	0.06854800	-1.54142200
C	-2.40510900	0.79345500	0.46762000
H	-2.90373100	1.75940700	0.39634600
H	-2.56281400	0.43247200	1.48915300
N	1.22997100	0.26335200	0.04923000
N	-0.97771700	1.06222600	0.24588900
C	-0.62696600	2.39370600	-0.26299700
H	-0.80488700	2.43907400	-1.34461900
H	-1.28975600	3.11903000	0.21209300
C	0.82618700	2.70699000	0.05256100
H	0.94583700	2.83104700	1.13368700
H	1.11826000	3.64436600	-0.42661700
C	1.70040400	1.55875000	-0.43289200
H	2.73074600	1.68504600	-0.09417100
H	1.72627600	1.54540700	-1.53057800
C	2.76415200	-1.19695700	-0.07364600
O	3.69839900	-0.57379400	0.29407400
O	2.22685200	-2.16291300	-0.49179400

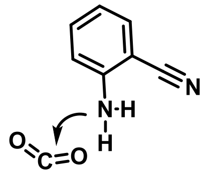

TS-a

C	0.08520400	-1.50562600	-0.17811800
C	0.09772000	-0.11527100	-0.11758300
C	1.32754100	0.55884400	0.01028300
C	2.52474500	-0.16892900	0.07698100
C	2.49845000	-1.55457500	0.01829900
C	1.27848100	-2.21785700	-0.11018700
H	-0.85279600	-2.03735200	-0.28006500
H	3.46063800	0.36604100	0.17504400
H	3.42344600	-2.11465800	0.07203800
H	1.25090000	-3.29976200	-0.15767200
C	1.36936700	1.98220600	0.06190600
N	1.38769800	3.13738800	0.10197600
N	-1.11952800	0.61875700	-0.23835200
H	-1.05730900	1.56332900	0.14743300
H	-1.93346000	0.57417200	-1.25513900
C	-2.46279500	-0.02847400	0.18117100
O	-2.72502600	-0.46707900	1.26312300
O	-3.08508500	0.08624300	-0.93856200

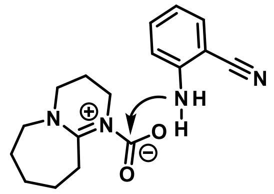

TS-b

C	-3.33366500	0.96837400	-0.62232900
C	-2.47310800	0.44622600	0.38281100
C	-2.83990000	-0.83526800	0.91388000
C	-4.00145500	-1.50870900	0.48819000
C	-4.81352000	-0.95819000	-0.48550600
C	-4.46004400	0.28669000	-1.03824000
H	-3.08304300	1.92758400	-1.05556100
H	-4.24249700	-2.46722800	0.93350700
H	-5.70507900	-1.47669700	-0.81573200
H	-5.08906600	0.72681400	-1.80523400
C	-2.00548200	-1.43375200	1.88960400
N	-1.29598300	-1.90451900	2.67782900
N	-1.38169900	1.14334700	0.77223900
H	-0.85101300	3.16431800	0.53978300
C	-0.19638200	1.85209300	-0.64837500
O	-0.47551200	1.44785400	-1.76541800
O	-0.42566300	3.18204400	-0.33118100
H	-0.82104000	0.62683900	1.44476500
C	1.46475300	0.09899700	-0.01333900
C	2.99078400	-1.71917400	0.63138700
H	2.17553700	-2.26874900	1.10517900
H	3.83405700	-1.74592900	1.31944800
C	3.37874600	-2.35350600	-0.70512000
H	3.98093900	-3.24144100	-0.48968200
H	4.02379400	-1.65563000	-1.25058800
C	2.17642300	-2.76019600	-1.55953000
H	2.53193800	-3.19117500	-2.50084300
H	1.63137800	-3.55534900	-1.03626100

C	1.19918200	-1.62339700	-1.86484600
H	0.37248500	-2.02054000	-2.45978100
H	1.68356100	-0.85708400	-2.47759900
C	0.58318500	-0.96520200	-0.61045700
H	-0.37009500	-0.53201600	-0.86902400
H	0.40449300	-1.72251200	0.15687900
N	1.09963800	1.38809600	-0.01756400
N	2.61941000	-0.29211500	0.52055200
C	3.65099600	0.64256800	1.00555200
H	3.63544900	0.63247400	2.09943600
H	4.61644600	0.25493400	0.67725200
C	3.40919800	2.03285000	0.46416300
H	3.66271000	2.07539600	-0.59866700
H	4.03962100	2.74968100	0.99257500
C	1.94905200	2.39572800	0.65064800
H	1.68772200	2.43663100	1.71300700
H	1.72896000	3.36005100	0.20757000

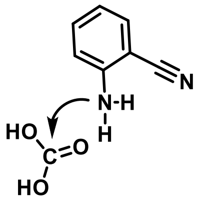

TS-c

C	-0.84471900	-1.57018300	-0.42471300
C	-0.46164800	-0.22964900	-0.45276200
C	-1.40249100	0.75326900	-0.07965200
C	-2.70286100	0.38645200	0.30000400
C	-3.06312000	-0.95270800	0.32389700
C	-2.13102800	-1.92749000	-0.03703900
H	-0.13040100	-2.32926000	-0.71633600
H	-3.41113000	1.15799100	0.57395100
H	-4.06599600	-1.23626600	0.61813600
H	-2.41041600	-2.97431700	-0.02413400
C	-1.03947100	2.13174100	-0.08827500
N	-0.73567600	3.24730700	-0.09922300
N	0.86075600	0.11184500	-0.82189100
H	1.70876000	-0.65086300	-1.40416300
C	2.17760600	-0.29282000	0.16571300
O	1.74040300	-1.02965700	1.22722600
O	2.78158000	-0.96940100	-0.79913500
O	2.77879400	0.86226500	0.61905500
H	1.50343700	-0.43415500	1.95473700
H	3.38264300	1.16798000	-0.07376000
H	0.94772900	1.08749700	-1.10082800

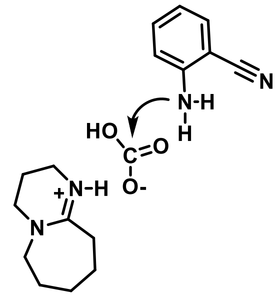

TS-d

C	-3.77009200	0.10563000	0.04219700
C	-2.41349300	0.26678000	0.47809700
C	-2.03459100	1.62395100	0.79689500
C	-2.94302700	2.69812900	0.67750800
C	-4.23631200	2.48905500	0.24407700
C	-4.63415500	1.17104700	-0.06994600
H	-4.09884700	-0.89547300	-0.20143400
H	-2.60232900	3.69488200	0.93570400
H	-4.92995400	3.31550700	0.15148400
H	-5.65005600	0.98962300	-0.40742500
C	-0.72944800	1.88915200	1.26814800
N	0.34461200	2.09830400	1.65883200
N	-1.60835300	-0.79577800	0.57652300
H	-1.26792600	-1.61703300	-2.17846400
C	-1.68726600	-2.47266500	-0.55222500
O	-2.07213300	-1.94290300	-1.75248800
O	-0.55266300	-2.94925900	-0.37985000
H	-0.64457300	-0.55772200	0.79468700
C	2.29370300	-0.74583300	-0.34234800
C	3.83241600	1.10768800	-0.77411000
H	4.23098100	0.61575200	-1.66479600
H	4.68197700	1.50288300	-0.21964700
C	2.87592000	2.23979700	-1.15627000
H	3.48372300	3.09248600	-1.47447900
H	2.32713300	2.55181700	-0.26213400
C	1.90577900	1.88012200	-2.28557600
H	1.27345900	2.74721800	-2.50052600
H	2.48705000	1.68293700	-3.19480200

C	1.00682600	0.67075100	-2.01412800
H	0.37149900	0.50364400	-2.88834500
H	0.33652600	0.86999500	-1.17387200
C	1.77601200	-0.63771200	-1.74758400
H	1.13069700	-1.49761900	-1.92142900
H	2.61580500	-0.72809500	-2.44299100
N	1.76431400	-1.67812800	0.43299300
N	3.21645000	0.09376900	0.10360200
C	3.64683400	0.08779900	1.51655500
H	4.60035000	-0.44473400	1.58379200
H	3.81697500	1.12561900	1.80379500
C	2.59591400	-0.54516000	2.41560400
H	1.73837600	0.12505700	2.50127500
H	3.01674200	-0.69271700	3.41129800
C	2.14554600	-1.87507800	1.83127200
H	2.94689700	-2.61824700	1.88583000
H	1.27748400	-2.27142300	2.35786800
H	0.95436200	-2.19796200	0.06836900
O	-2.79779700	-3.05900700	-0.01368700
H	-2.51815600	-3.43494500	0.83178500

References

[1] Tsintzou GP, Antonakou EV, Achilias DS. Environmentally friendly chemical recycling of poly(bisphenol-A carbonate) through phase transfer-catalysed alkaline hydrolysis under microwave irradiation. J Hazard Mater 2012; 241: 137-145.

[2] Liu FS, Li Z, Yu ST, et al. Methanolysis and hydrolysis of polycarbonate under moderate conditions. J Polym Environ 2009; 17: 208-211.

[3] Emami S, Nikje MMA. Magnetic $Fe_3O_4/SiO_2/NH_2$ as the recyclable heterogeneous nanocatalyst on bisphenol-A recovery from polycarbonate wastes. Russ J Appl Chem 2018; 91: 159-166.

[4] Iannone F, Casiello M, Monopoli A, et al. Ionic liquids/ZnO nanoparticles as recyclable catalyst for polycarbonate depolymerization. J Mol Catal A-Chem 2017; 426: 107-116.

[5] Li L, Liu FS, Li Z, et al. Hydrolysis of polycarbonate using ionic liquid [Bmim][Cl] as solvent and catalyst. Fiber Polym 2013; 14: 365-368.

[6] Song XY, Liu FS, Li L, et al. Hydrolysis of polycarbonate catalyzed by ionic liquid [Bmim][Ac]. J Hazard Mater 2013; 244: 204-208.

[7] Taguchi M, Ishikawa Y, Kataoka S, et al. CeO_2 nanocatalysts for the chemical recycling of polycarbonate. Catal Commun 2016; 84: 93-97.

[8] Quaranta E. Rare Earth metal triflates $M(O_3SCF_3)_3$<(M = Sc, Yb, La) as Lewis acid catalysts of depolymerization of poly-(bisphenol A carbonate) via hydrolytic cleavage of carbonate moiety: Catalytic activity of $La(O_3SCF_3)_3$. Appl Catal B Environ Energy 2017; 206: 233-241.

[9] Liu MS, Guo J, Gu YQ, et al. Degradation of waste polycarbonate via hydrolytic strategy to recover monomer (bisphenol A) catalyzed by DBU-based ionic liquids under metal- and solvent-free conditions. Polym Degrad Stabil 2018; 157: 9-14.

[10] Quaranta E, Mesto E, Lacalamita M, et al. Using a natural chlorite as catalyst in chemical recycling of waste plastics: Hydrolytic depolymerization of poly- bisphenol A carbonate promoted by clinochlore. Waste Manage 2021; 120: 642-649.

[11] Abedsoltan H. Concentrated sulfuric acid as a catalyst for chemical recycling of polycarbonate in water. Waste Biomass Valorization 2024; 15: 2793-2806.

[12] Emami S, Nikje MMA. Environmentally benign chemical recycling of polycarbonate wastes: comparison of micro-and nano-TiO_2 solid support efficiencies. Green Process Synth 2019; 8: 108-117.

[13] Pan ZY, Chou IM, Burruss RC. Hydrolysis of polycarbonate in sub-critical water in fused silica capillary reactor with *in situ* Raman spectroscopy. Green Chem 2009; 11: 1105-1107.

[14] Ma J, Han BX, Song JL, et al. Efficient synthesis of quinazoline-2,4(1*H*,3*H*)-diones from CO_2 and 2-aminobenzonitriles in water without any catalyst. Green Chem 2013; 15: 1485-1489.

[15] Goto S, Tsuboi H, Kanoda M, et al. The process development of a novel aldose reductase inhibitor, FK366. Part 1. Improvement of discovery process and new syntheses of 1-substituted quinazolinediones. Org Process Res Dev 2003; 7: 700-706.

[16] Mizuno T, Mihara M, Nakai T, et al. Solvent-free synthesis of quinazoline-2,4(1*H*,3*H*)-diones using carbon dioxide and a catalytic amount of DBU. Synthesis 2007; 16: 2524-2528.

[17] Zhu LN, Jin J, Liu C, et al. Synthesis and biological evaluation of novel quinazoline-derived human Pin1 inhibitors. Bioorg Med Chem 2011; 19: 2797-2807.

[18] Shi DQ, Dou GL, Li ZY, et al. An efficient synthesis of quinazoline-2,4-dione derivatives with the aid of a low-valent titanium reagent. Tetrahedron 2007; 63: 9764-9773.

[19] Vorbruggen H, Krolikiewicz K. The introduction of nitrile groups into heterocycles and conversion of carboxylic groups into their corresponding nitriles with chlorosulfonylisocyanate and triethylamine. Tetrahedron 1994; 50: 6549-6558.

[20] Li JR, Chen X, Shi DX, et al. A new and facile synthesis of quinazoline-2,4(1*H*,3*H*)-diones. Org Lett 2009; 11: 1193-1196.

[21] Li ZG, Huang H, Sun HB, et al. Microwave-assisted efficient and convenient synthesis of 2,4(1*H*,3*H*)-quinazolinediones and 2-thioxoquinazolines. J Comb Chem 2008; 10: 484-486.

[22] Akazome M, Yamamoto J, Kondo T, et al. Palladium complex-catalyzed intermolecular reductive N-heterocyclization – novel synthesis of quinazoline derivatives from 2-nitrobenzaldehyde or 2-nitrophenyl ketones with formamide. J Organomet Chem 1995; 494: 229-233.

[23] Patil YP, Tambade PJ, Jagtap SR, et al. Cesium carbonate catalyzed efficient synthesis of quinazoline-2,4(1*H*,3*H*)-diones using carbon dioxide and 2-aminobenzonitriles. Green Chem Lett Rev 2008; 1: 127-132.

[24] Patil YP, Tambade PJ, Parghi KD, et al. Synthesis of quinazoline-2,4(1*H*,3*H*)-diones from carbon dioxide and 2-aminobenzonitriles using MgO/ZrO_2 as a solid base catalyst. Catal Lett 2009; 133: 201-208.

[25] Kimura T, Kamata K, Mizuno N. A bifunctional tungstate catalyst for chemical fixation of CO_2 at atmospheric pressure. Angew Chem Int Ed 2012; 51: 6700-6703.

[26] Zhao YN, Yu B, Yang ZZ, et al. Magnetic base catalysts for the chemical fixation of carbon dioxide to quinazoline-2,4(1*H*,3*H*)-diones. RSC Adv 2014; 4: 28941-28946.

[27] Mizuno T, Okamoto N, Ito T, et al. Synthesis of 2,4-dihydroxyquinazolines using carbon dioxide in the presence of DBU under mild conditions. Tetrahedron Lett 2000; 41: 1051-1053.

[28] Patil YP, Tambade PJ, Deshmukh KM, et al. Synthesis of quinazoline-2,4(1*H*,3*H*)-diones from carbon dioxide and 2-aminobenzonitriles using [Bmim]OH as a homogeneous recyclable catalyst. Catal Today 2009; 148: 355-360.

[29] Gao JA, He LN, Miao CX, et al. Chemical fixation of CO_2: efficient synthesis of quinazoline-2,4(1*H*, 3*H*)-diones catalyzed by guanidines under solvent-free conditions. Tetrahedron 2010; 66: 4063-4067.

[30] Xiao YQ, Kong XQ, Xu ZC, et al. Efficient synthesis of quinazoline-2,4(1*H*,3*H*)-diones from CO_2 catalyzed by *N*-heterocyclic carbene at atmospheric pressure. RSC Adv 2015; 5: 5032-5037.

[31] Sadeghzadeh SM. Spidery catalyst for the synthesis of quinazoline-2,4(1*H*,3*H*)-diones. Catal Sci Technol 2016; 6: 1435-1441.

[32] Rasal KB, Yadav GD. Carbon dioxide mediated novel synthesis of quinazoline-2,4(1*H*,3*H*)-dione in Water. Org Process Res Dev 2016; 20: 2067-2073.

[33] Zhao YF, Yu B, Yang ZZ, et al. A protic ionic liquid catalyzes CO_2 conversion at atmospheric pressure and room temperature: synthesis of quinazoline-2,4-(1*H*,3*H*)-diones. Angew Chem Int Ed 2014; 53: 5922-5925.

[34] Kumar S, Verma S, Shawat E, et al. Amino-functionalized carbon nanofibres as an efficient metal free catalyst for the synthesis of quinazoline-2,4(1*H*,3*H*)-diones from CO_2 and 2-aminobenzonitriles. RSC Adv 2015; 5: 24670-24674.

[35] Lu WJ, Ma J, Hu JY, et al. Choline hydroxide promoted chemical fixation of CO_2 to quinazoline-2,4(1*H*,3*H*)-diones in water. RSC Adv 2014; 4: 50993-50997.

[36] Nale DB, Saigaonkar SD, Bhanage BM. An efficient synthesis of quinazoline-2,4(1*H*,3*H*)-dione from CO_2 and 2-aminobenzonitrile using Hmim OH/SiO_2 as a base functionalized Supported Ionic Liquid Phase Catalyst. J CO2 Util 2014; 8: 67-73.

[37] Gao X, Zhao JJ, Liu J, et al. A bifunctional [Ch][Triz] ionic liquid catalyst for CO_2 transformation into quinazoline-2,4(1H,3H)-diones under mild conditions. New J Chem 2024; 48: 17773-17778.

[38] Pérez ER, da Silva MO, Costa VC, et al. Efficient and clean synthesis of *N*-alkyl carbamates by transcarboxylation and *O*-alkylation coupled reactions using a DBU-CO_2 zwitterionic carbamic complex in aprotic polar media. Tetrahedron Lett 2002; 43: 4091-4093.

[39] Villiers C, Dognon JP, Pollet R, et al. An isolated CO_2 adduct of a nitrogen base: crystal and electronic structures. Angew Chem Int Ed 2010; 49: 3465-3468.

[40] Gomes CD, Blondiaux E, Thuéry P, et al. Metal-free reduction of CO_2 with hydroboranes: two efficient pathways at play for the reduction of CO_2 to methanol. Chem-Eur J 2014; 20: 7098-7106.

[41] Chen XC, Zhao KC, Yao YQ, et al. Synergetic activation of CO_2 by the DBU-organocatalyst and amine substrates towards stable carbamate salts for synthesis of oxazolidinones. Catal Sci Technol 2021; 11: 7072-7082.

[42] Wu PX, Cheng HY, Shi RH, et al. Synthesis of polyurea via the addition of carbon dioxide to a diamine catalyzed by organic and inorganic bases. Adv Synth Catal 2019; 361: 317-325.

[43] Chen TT, Zhang YF, Xu YJ. Efficient synthesis of quinazoline-2,4(1*H*,3*H*)-dione via simultaneous activated CO_2 and 2-aminobenzonitrile by 1-methylhydantoin anion-functionalized ionic liquid through the multiple-site cooperative interactions. ACS Sustain Chem Eng 2022; 10: 10699-10711.

[44] Chen TT, Guo YJ, Xu YJ. Efficient catalytic conversion of CO_2 to quinazoline-2,4(1*H*,3*H*)-diones by a dual-site anion-functionalized ionic liquid: reconsidering the mechanism. Chem Commun 2023; 59: 12282-12285.

[45] Fanjul-Mosteirín N, Jehanno C, Ruipérez F, et al. Rational study of DBU salts for the CO_2 insertion into epoxides for the synthesis of cyclic carbonates. ACS Sustain Chem Eng 2019; 7: 10633-10640.

[46] Yu B, Zhang HY, Zhao YF, et al. DBU-based ionic-liquid-catalyzed carbonylation of *o*-phenylenediamines with CO_2 to 2-benzimidazolones under solvent-free conditions. ACS Catal 2013; 3: 2076-2082.

[47] Ci YH, Chen TY, Li FY, et al. Cellulose dissolution and regeneration behavior via DBU-levulinic acid solvents. Int J Biol Macromol 2023; 252: 126548.
